# Supplementary material for: Development of GS-441524 Derivatives as Potent SARS-CoV-2 Mac1 Inhibitors via a Direct-to-Biology Approach
Source: bioRxiv. 2026 Jun 25:2026.06.24.734322. Preprint. [Version 1] doi: 10.64898/2026.06.24.734322 (PMC13320892; doi:10.64898/2026.06.24.734322)

Development of GS-441524 Derivatives as Potent SARS-CoV-2 Mac1 Inhibitors via a Direct-to-Biology Approach

Kewen Peng[1,2,4], Suryadeep Chakraborty[3], Shamar D. Wallace[5], Jessica Caroline Gomes Noll[6], Jialin Shang[4], Xuan Lu[1], Annette Choi[6], Gary Whittaker[6], J. Christopher Fromme[5], Hening Lin[1,2,3,4,7,*]

[1]Department of Medicine, The University of Chicago, Chicago, IL 60637, USA

[2]Center for Chemical Biology and Therapeutics, The University of Chicago, Chicago, IL 60637, USA

[3]Department of Chemistry, The University of Chicago, Chicago, IL 60637, USA

[4]Department of Chemistry and Chemical Biology, Cornell University, Ithaca, NY 14853, USA

[5]Department of Molecular Biology and Genetics, Weill Institute for Cell and Molecular Biology, Cornell University, Ithaca, NY 14853, USA

[6]Departments of Microbiology & Immunology, Cornell University College of Veterinary Medicine, Ithaca NY, USA

[7]Howard Hughes Medical Institute, Department of Medicine, Department of Chemistry, The University of Chicago, Chicago, IL 60637, USA

*Correspondence: linh1@uchicago.edu

Table 1. Data collection and refinement statistics

(values for the highest-resolution shell are shown in parentheses)

**Wavelength (Å)**	0.9686
**Resolution range (Å)**	29.07 - 1.526 (1.56 - 1.53)
**Space group**	P 1 2_1 1
**Unit cell**	42.91Å 88.90Å 43.04 Å<br>90.00° 94.88° 90.00°
**Total reflections**	289424 (9284)
**Unique reflections**	46501 (2249)
**Multiplicity**	6.2 (4.1)
**Completeness (%)**	95.21 (65.94)
**Mean I/sigma(I)**	11.30 (1.77)
**Wilson B-factor**	17.00
**R-merge**	0.08087 (0.4605)
**R-meas**	0.08792 (0.5264)
**R-pim**	0.03408 (0.2452)
**CC1/2**	0.998 (0.715)
**CC***	0.999 (0.913)
**Reflections used in refinement**	46498 (2284)
**Reflections used for R-free**	2007 (86)
**R-work**	0.1603 (0.2483)
**R-free**	0.1887 (0.2431)
**Number of non-hydrogen atoms**	2940
**Protein residues**	335
**RMS(bonds)**	0.005
**RMS(angles)**	0.75
**Ramachandran favored (%)**	99.40
**Ramachandran allowed (%)**	0.60
**Ramachandran outliers (%)**	0.00
**Rotamer outliers (%)**	0.00
**Clashscore**	1.70
**Average B-factor**	20.63

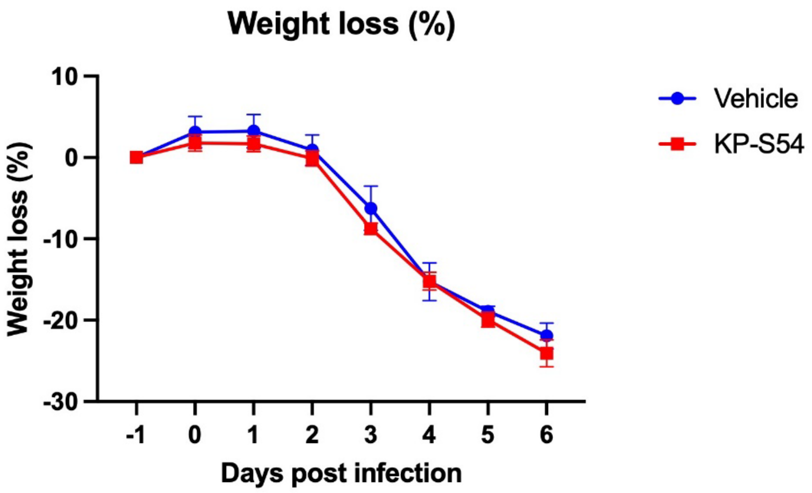

Figure S1. Effects of KP-S54 at 50 mg/kg and the vehicle on mouse body weight after SARS-CoV-2 infection (n = 5).

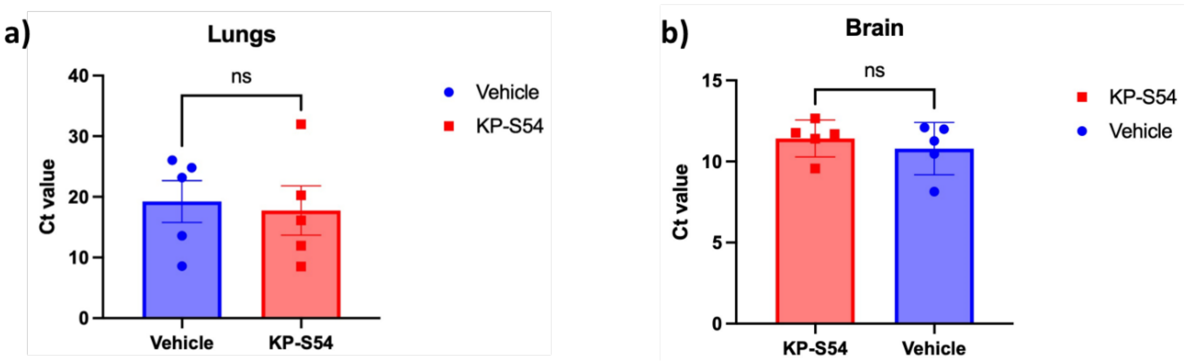

Figure S2. Effects of KP-S54 and the vehicle on SARS-CoV-2 viral loads in mouse **(a)** lungs and **(b)** brains (n = 5).

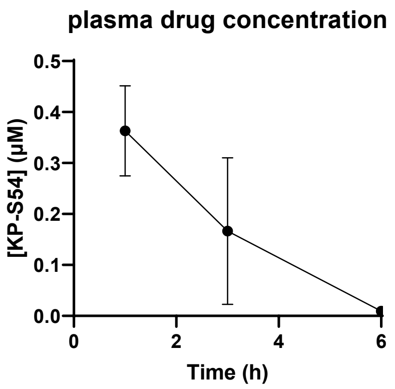

Figure S3. KP-S54 concentration in mouse plasma over time following intraperitoneal injection at 50 mg/kg (n = 2).

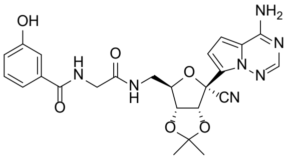

7

KP106CR74.10.fid

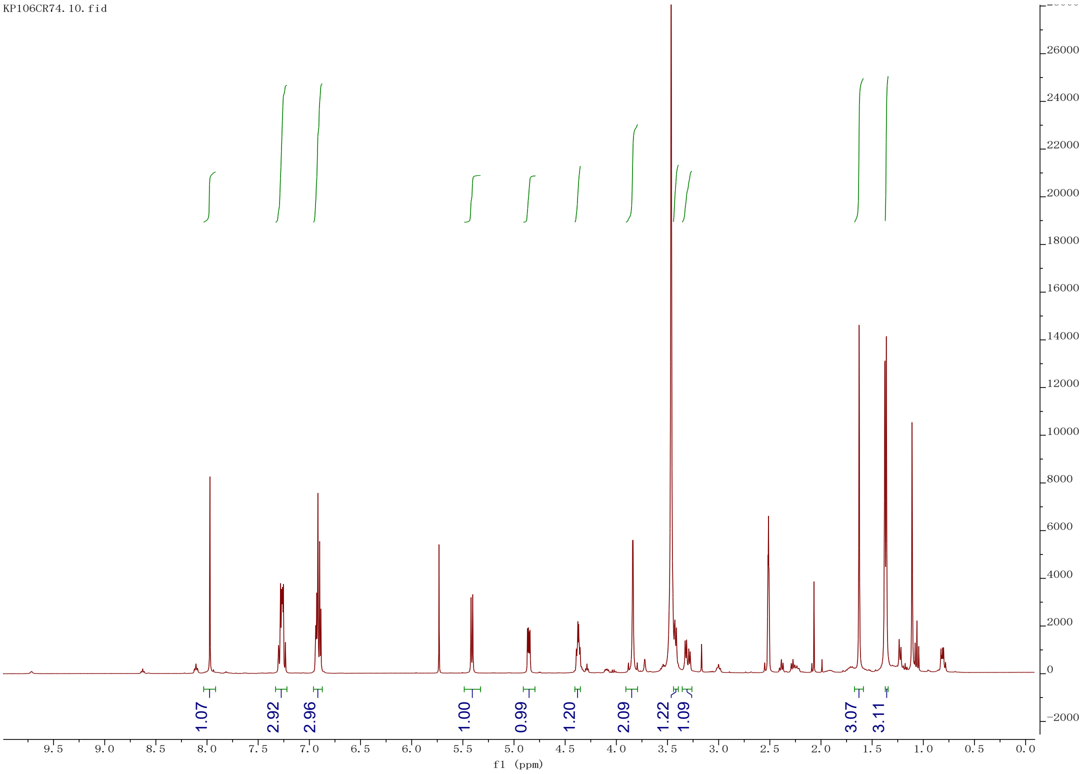

KP106CR74.12.fid

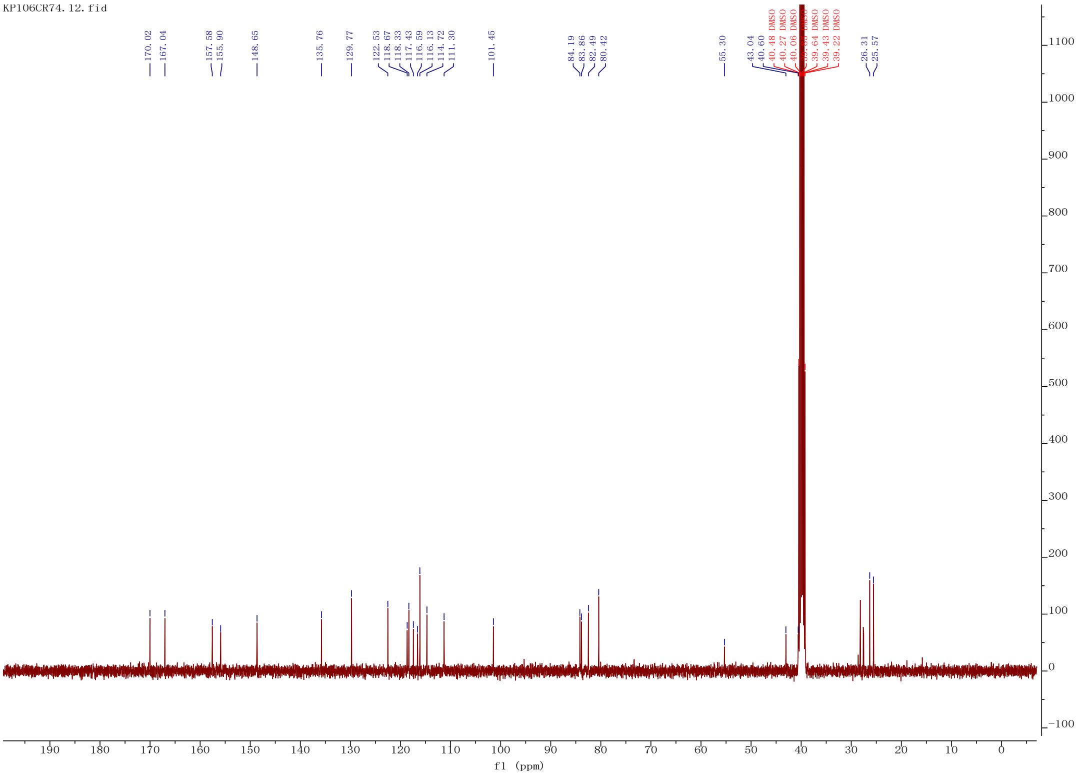

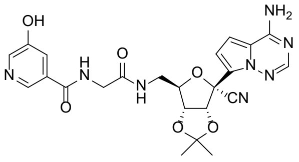
OH
NH2
N
N
N
H
O
O
N
H
N
O
CN
O
O

12a

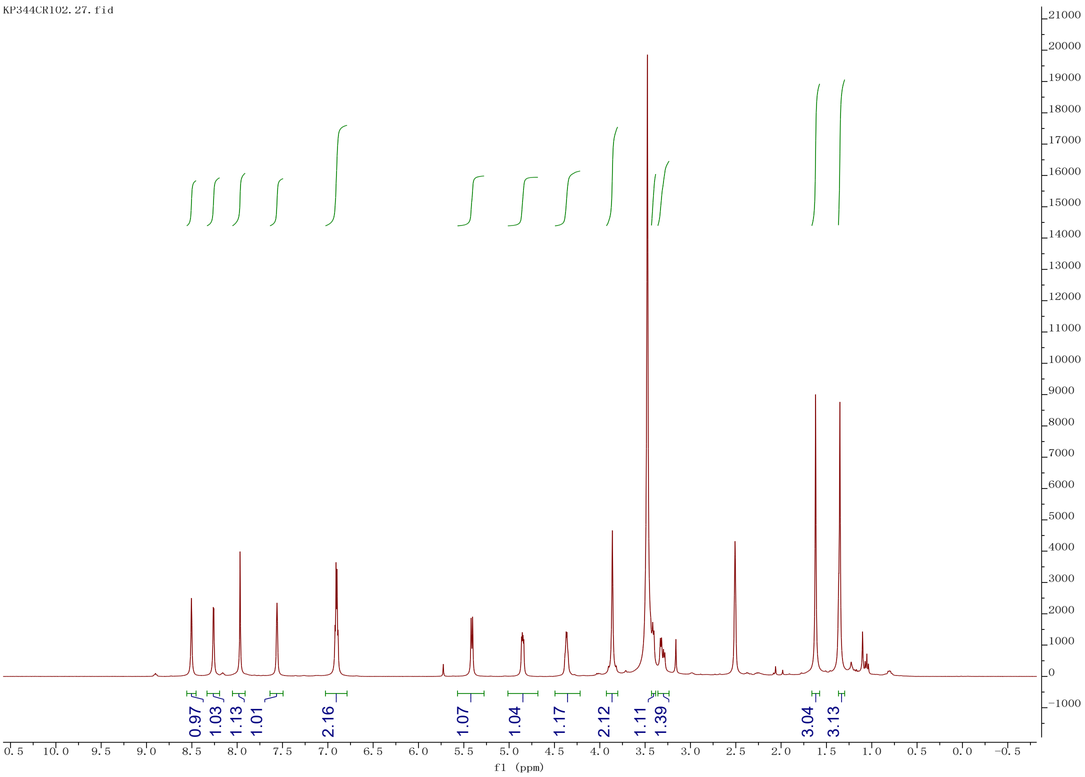
KP344CR102.27.fid
0.97
1.03
1.13
1.01
2.16
1.07
1.04
1.17
2.12
1.11
1.39
3.04
3.13
f1 (ppm)

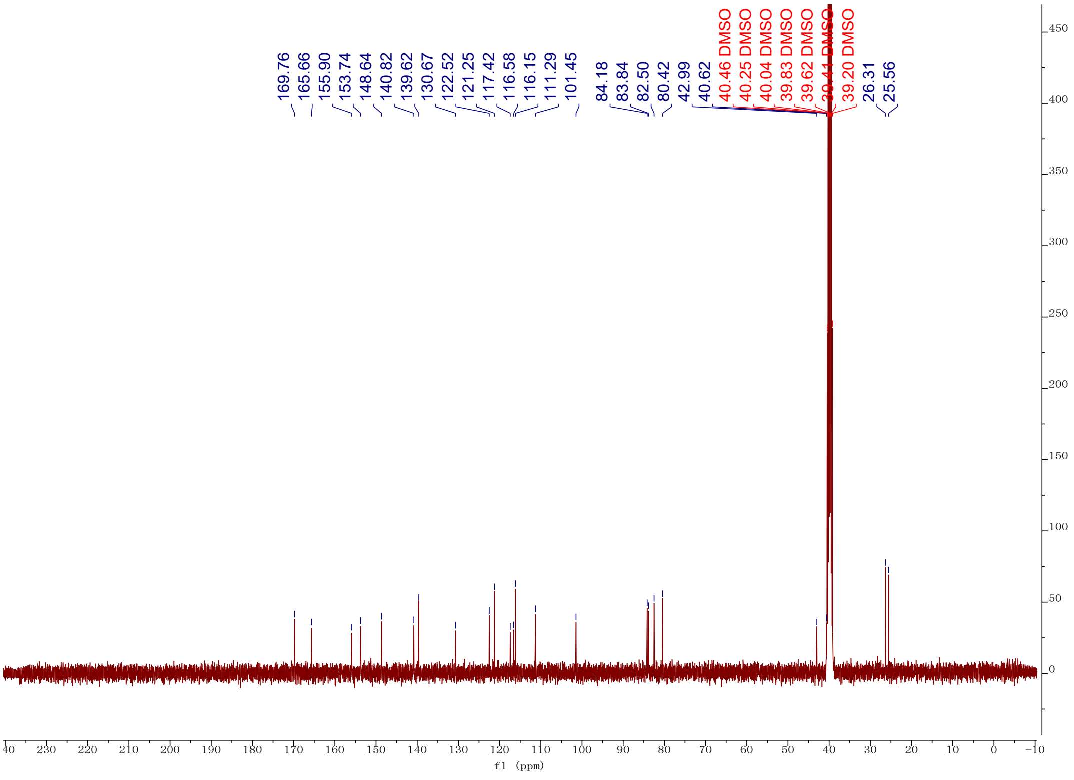
169.76
165.66
155.90
153.74
148.64
140.82
139.62
130.67
122.52
121.25
117.42
116.58
116.15
111.29
101.45
84.18
83.84
82.50
80.42
42.99
40.62
40.46 DMSO
40.25 DMSO
40.04 DMSO
39.83 DMSO
39.62 DMSO
39.41 DMSO
39.20 DMSO
26.31
25.56
f1 (ppm)

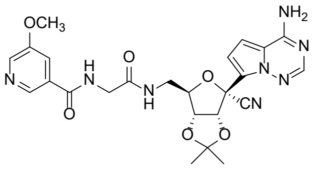
OCH3
NH2
N
H
O
N
N
O
N
N
N
H
O
CN
O
O

12b

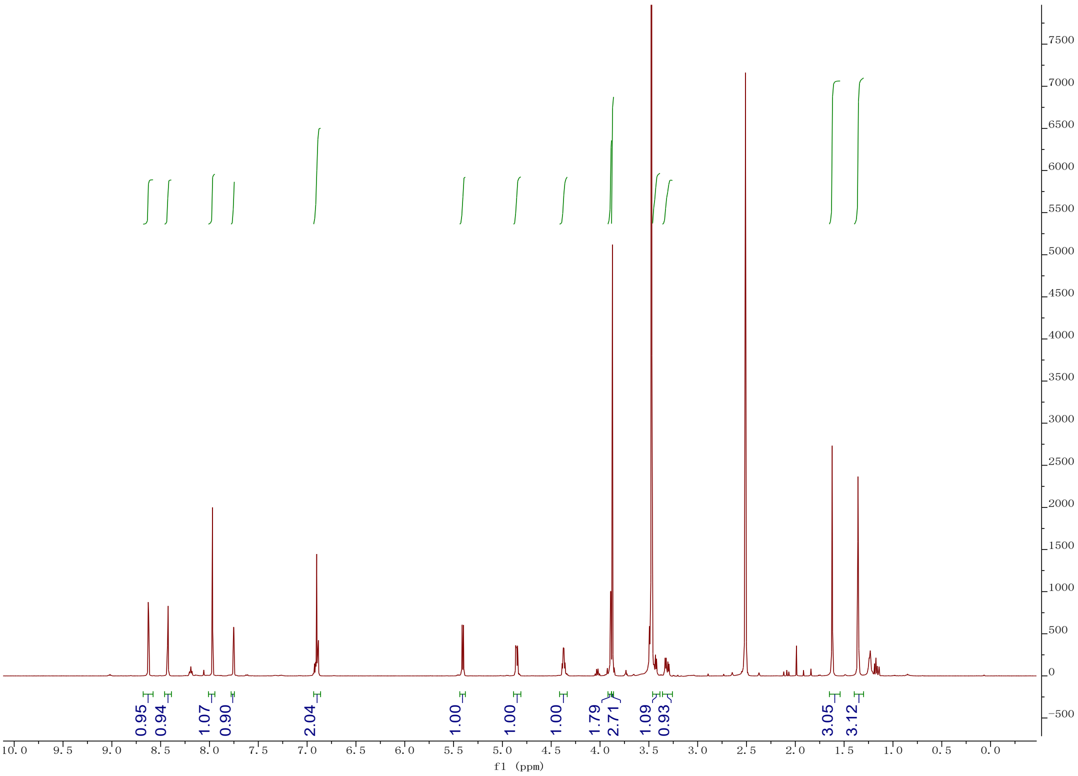
0.95
0.94
1.07
0.90
2.04
1.00
1.00
1.00
1.79
2.71
1.09
0.93
3.05
3.12
f1 (ppm)

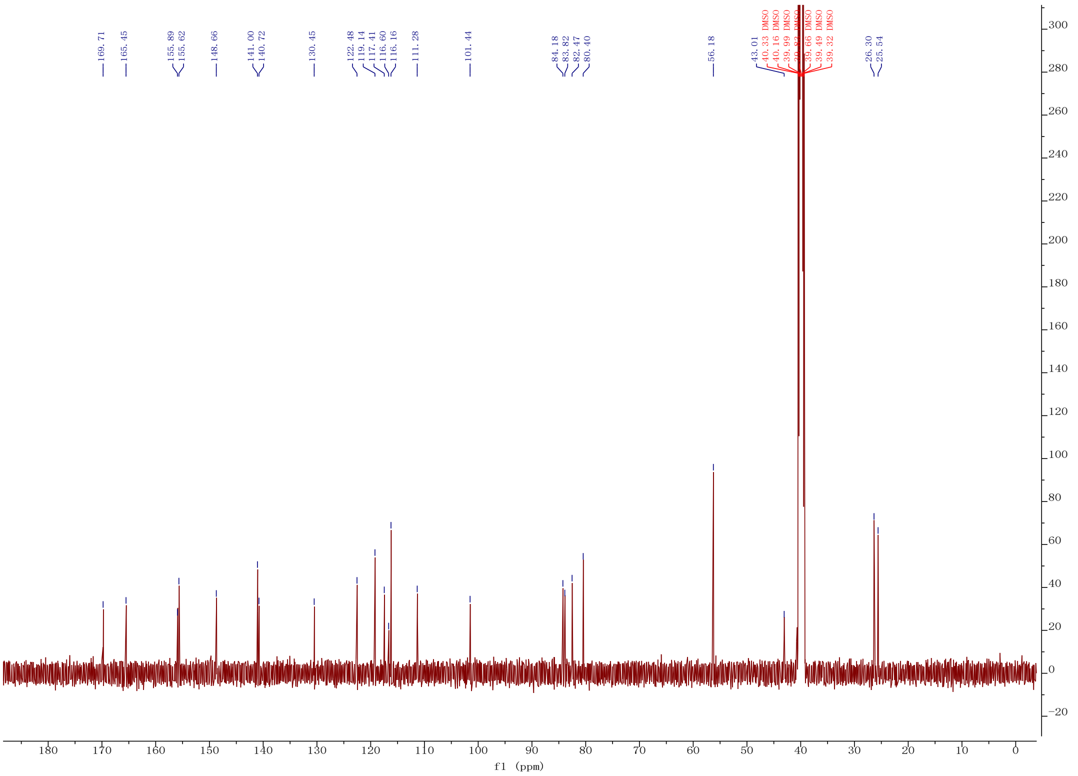
169.71
165.45
155.89
155.62
148.66
141.00
140.72
130.45
122.48
119.14
117.41
116.60
116.16
111.28
101.44
84.18
83.82
82.47
80.40
56.18
43.01
40.33 DMSO
40.16 DMSO
39.99 DMSO
39.83 DMSO
39.66 DMSO
39.49 DMSO
39.32 DMSO
26.30
25.54
f1 (ppm)

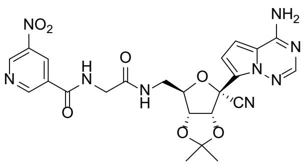
NO2
NH2
N
H
N
O
CN

12c

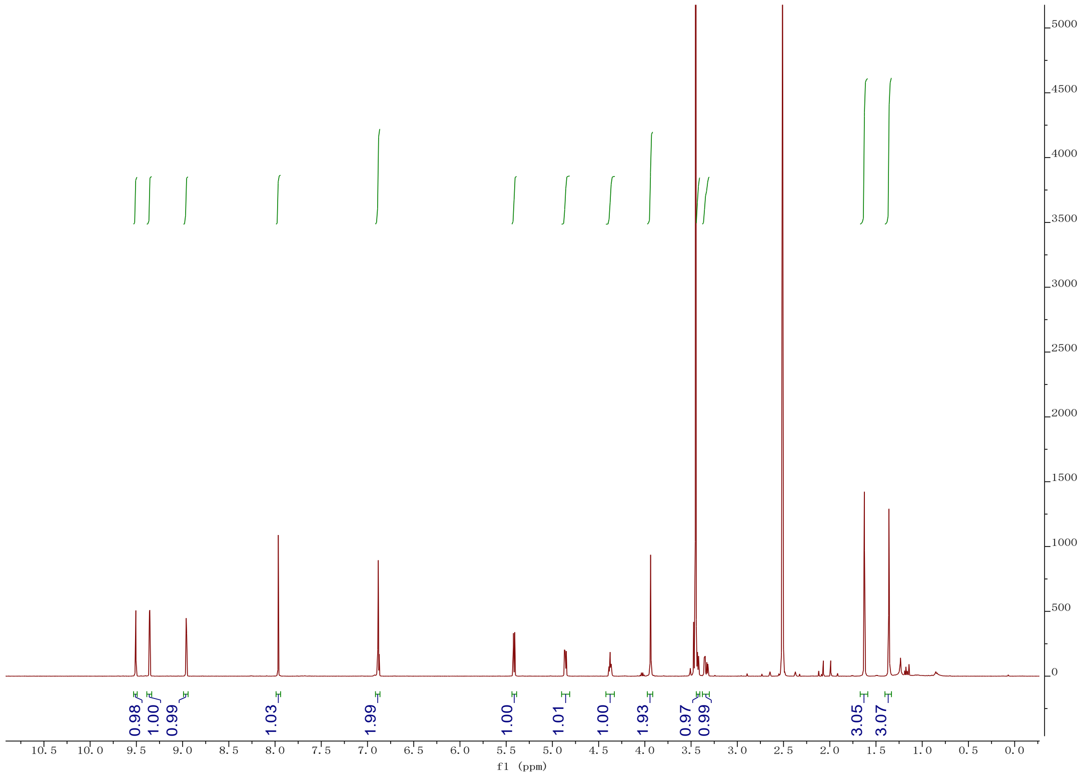
0.98
1.00
0.99
1.03
1.99
1.00
1.01
1.00
1.93
0.97
0.99
3.05
3.07
f1 (ppm)

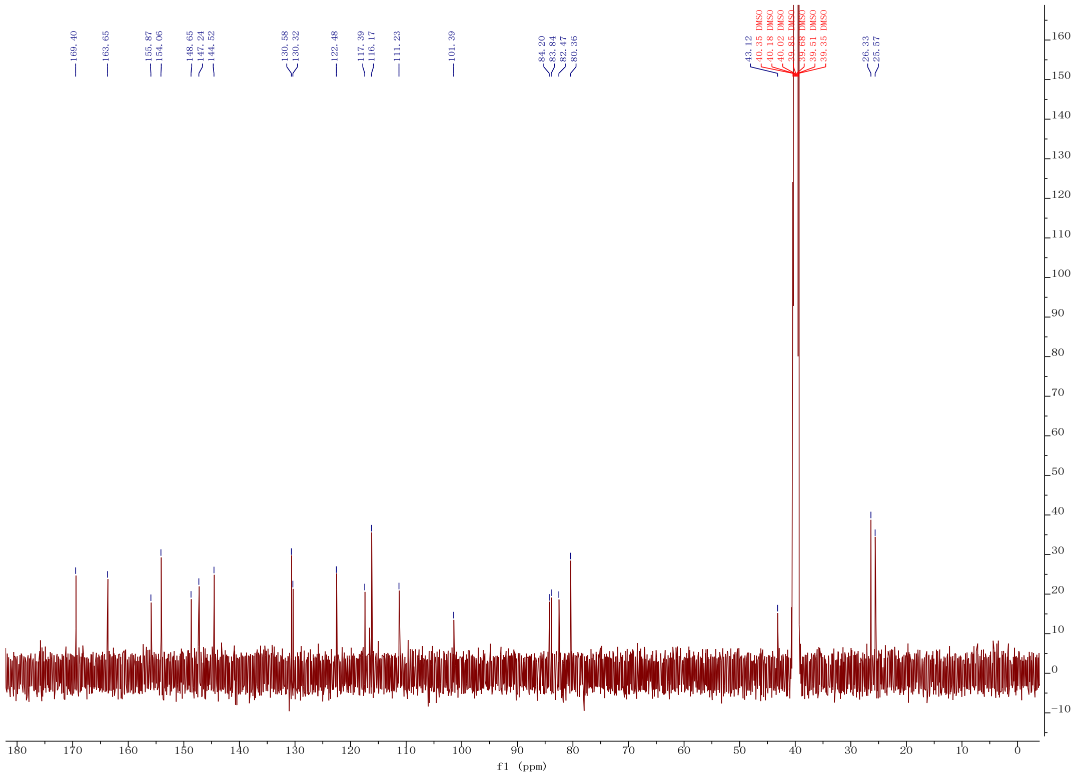
169.40
163.65
155.87
154.06
148.65
147.24
144.52
130.58
130.32
122.48
117.39
116.17
111.23
101.39
84.20
83.84
82.47
80.36
43.12
40.35 DMSO
40.18 DMSO
40.02 DMSO
39.85 DMSO
39.68 DMSO
39.51 DMSO
39.35 DMSO
26.33
25.57
f1 (ppm)

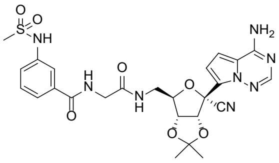

12d

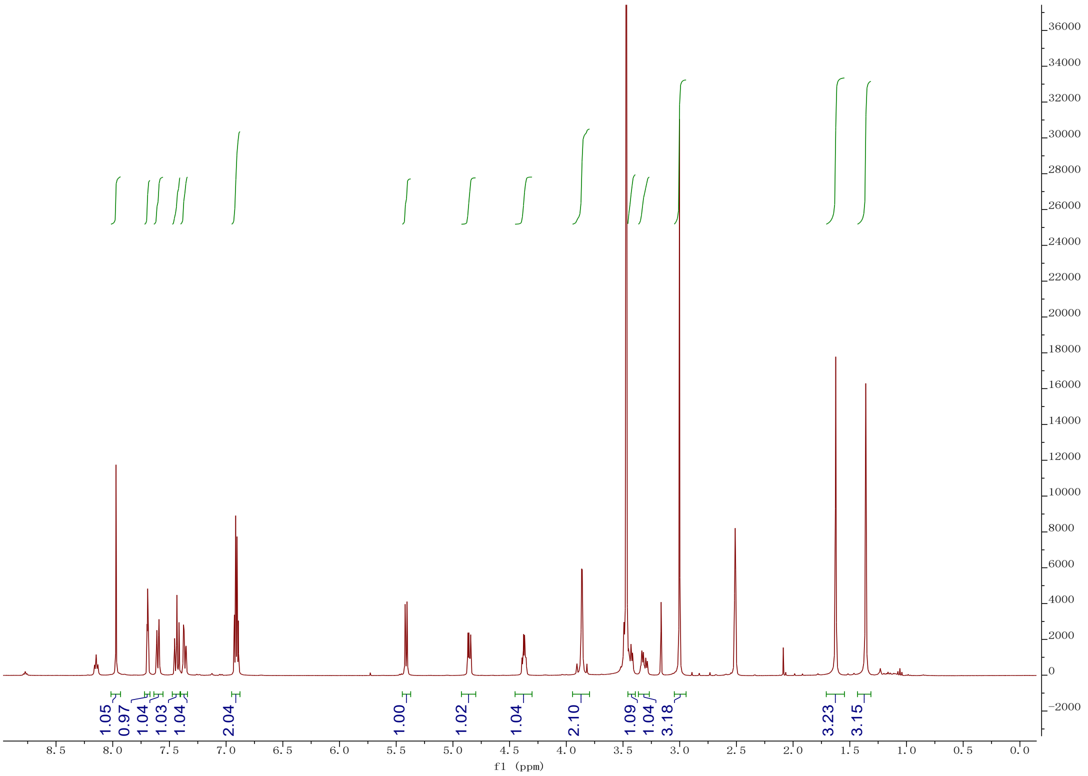

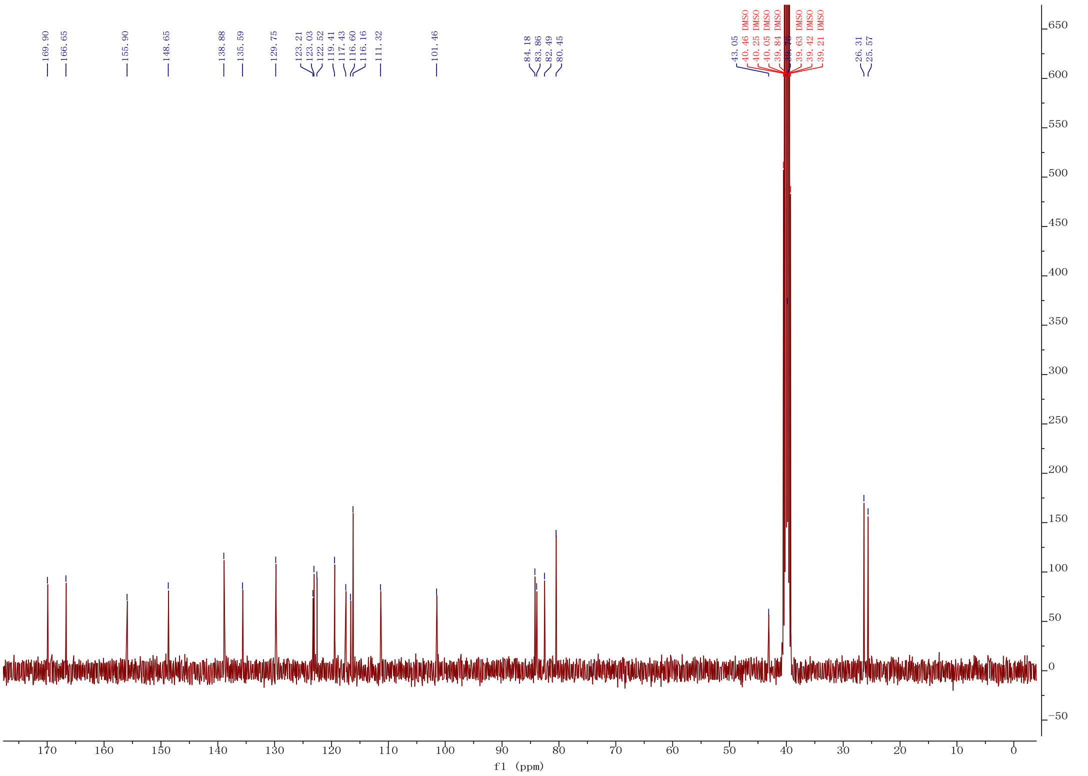

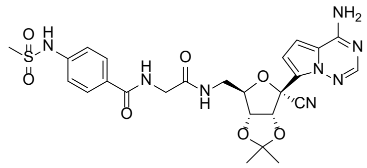
NH2
N
N
N
O
H
N
S
O
O
H
N
O
O
N
H
O
CN
O
O

12e

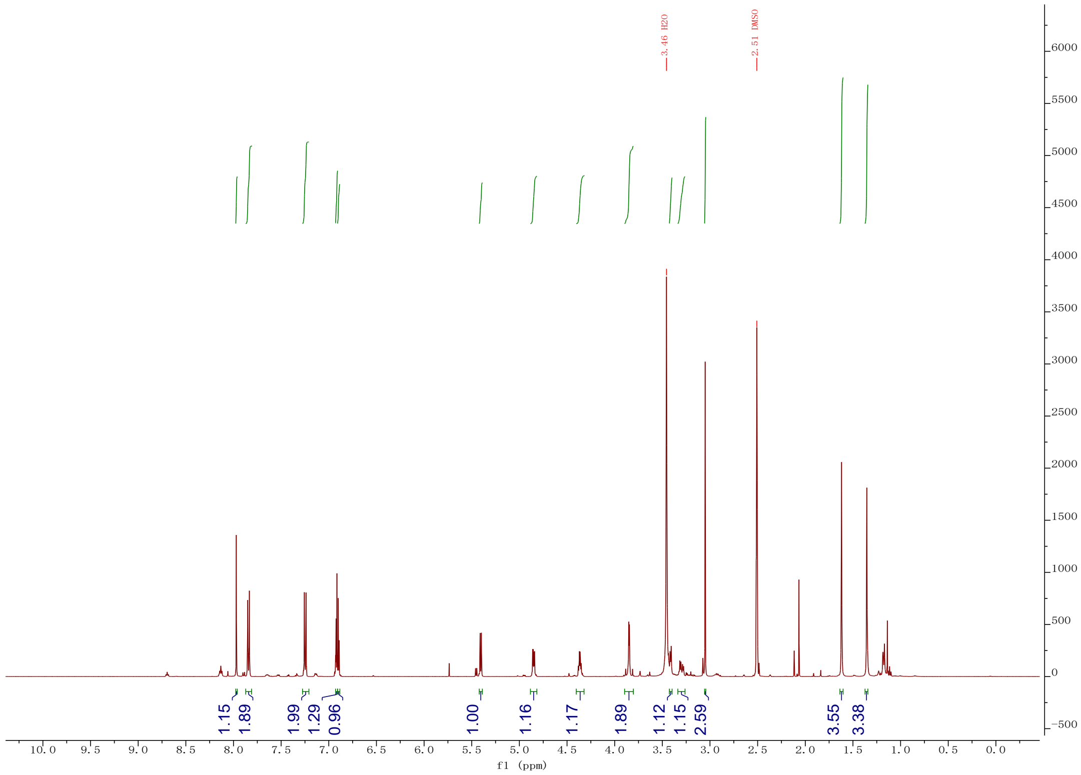
3.46 H2O
2.51 DMSO
1.15
1.89
1.99
1.29
0.96
1.00
1.16
1.17
1.89
1.12
1.15
2.59
3.55
3.38
f1 (ppm)

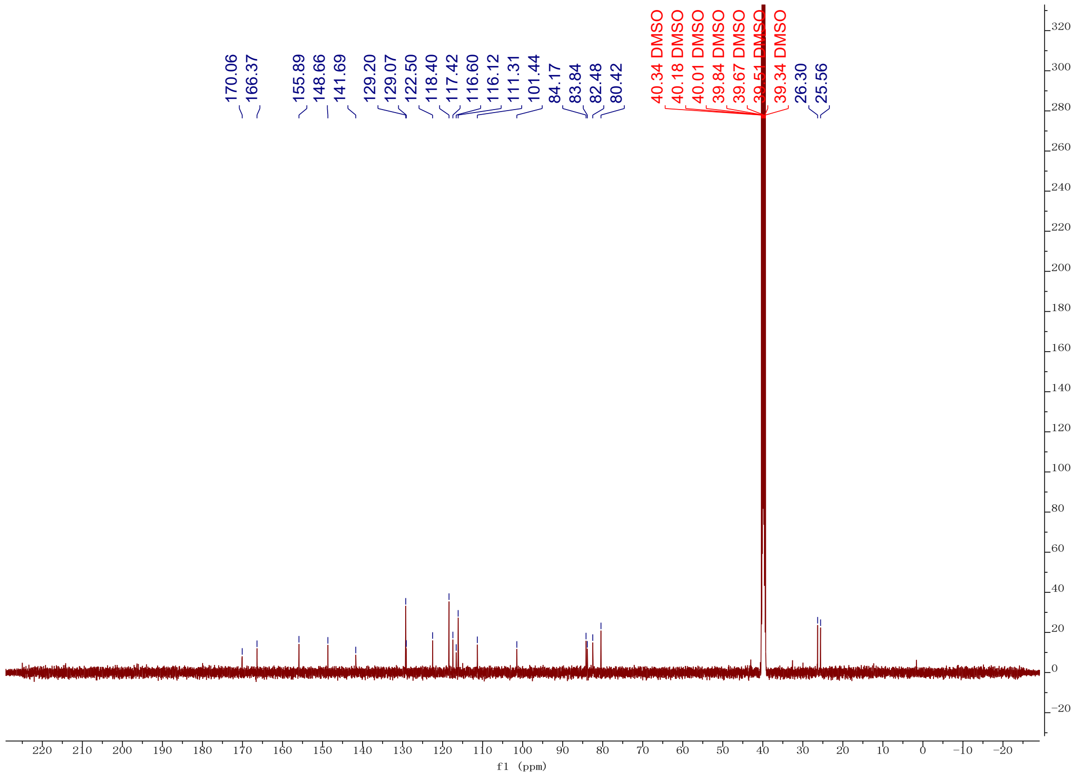
170.06
166.37
155.89
148.66
141.69
129.20
129.07
122.50
118.40
117.42
116.60
116.12
111.31
101.44
84.17
83.84
82.48
80.42
40.34 DMSO
40.18 DMSO
40.01 DMSO
39.84 DMSO
39.67 DMSO
39.51 DMSO
39.34 DMSO
26.30
25.56
f1 (ppm)

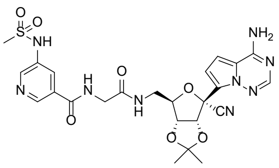

12f

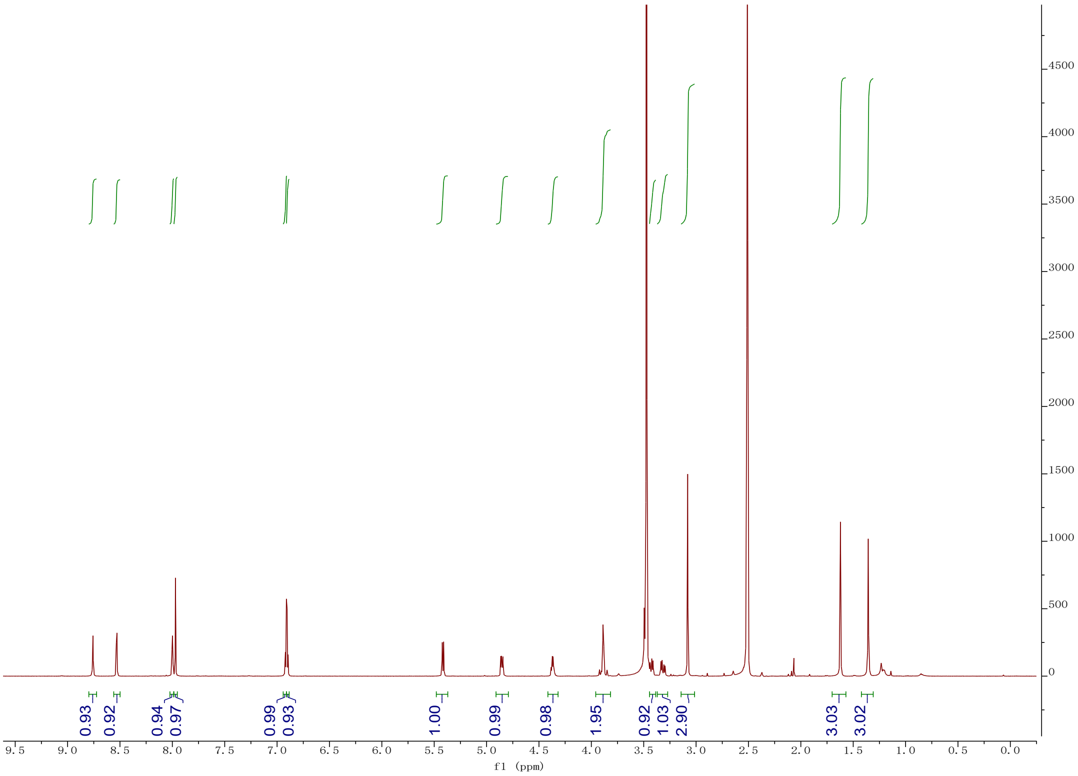
0.93
0.92
0.94
0.97
0.99
0.93
1.00
0.99
0.98
1.95
0.92
1.03
2.90
3.03
3.02
f1 (ppm)

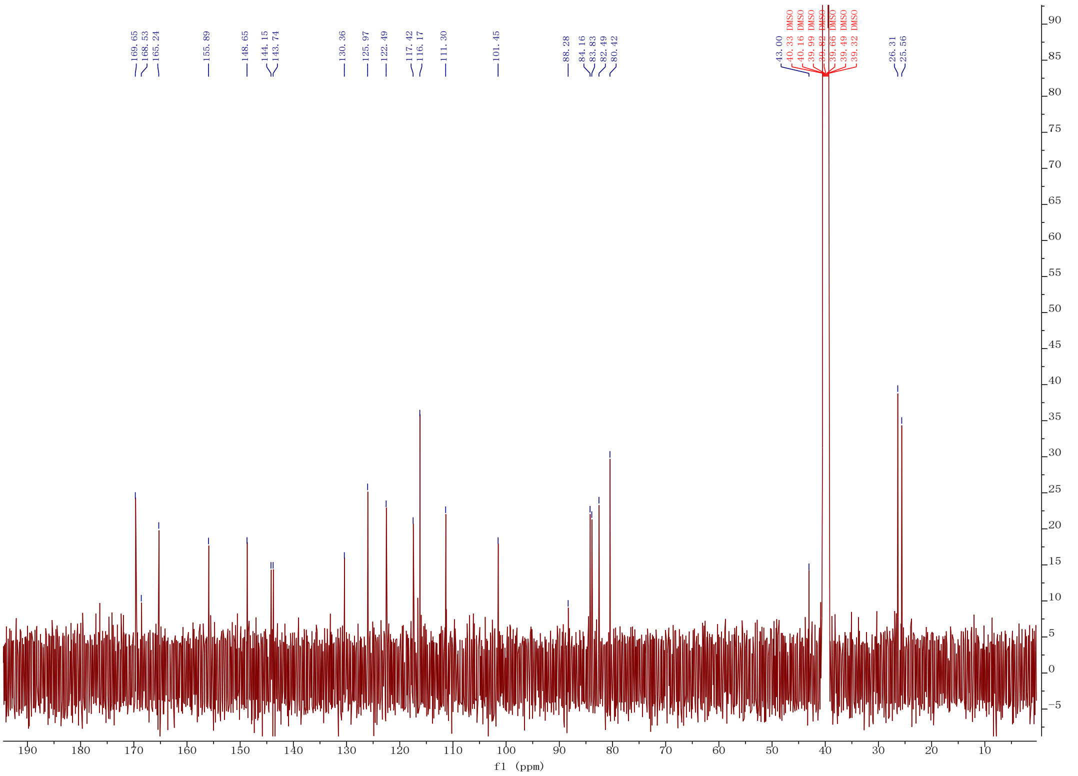
169.65
168.53
165.24
155.89
148.65
144.15
143.74
130.36
125.97
122.49
117.42
116.17
111.30
101.45
88.28
84.16
83.83
82.49
80.42
43.00
40.33 DMSO
40.16 DMSO
39.99 DMSO
39.82 DMSO
39.66 DMSO
39.49 DMSO
39.32 DMSO
26.31
25.56
f1 (ppm)

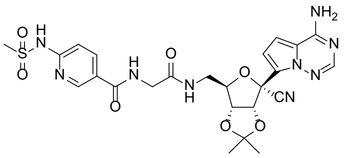

12g

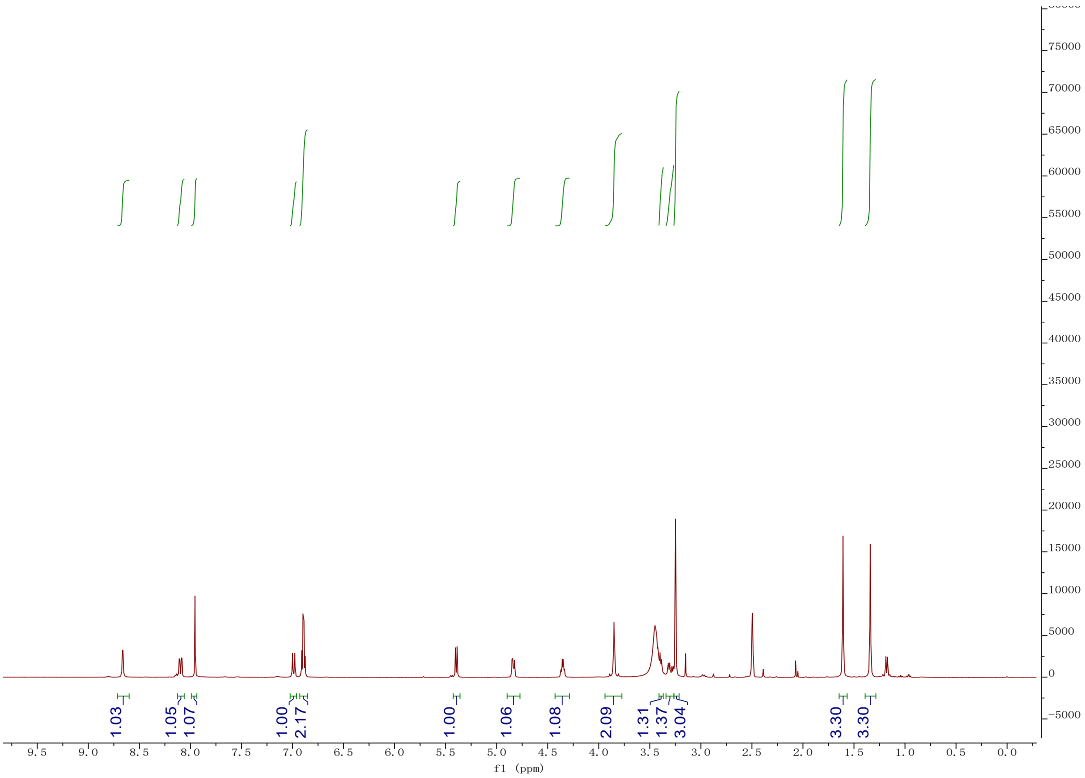

1.03
1.05
1.07
1.00
2.17
1.00
1.06
1.08
2.09
1.31
1.37
3.04
3.30
3.30
f1 (ppm)

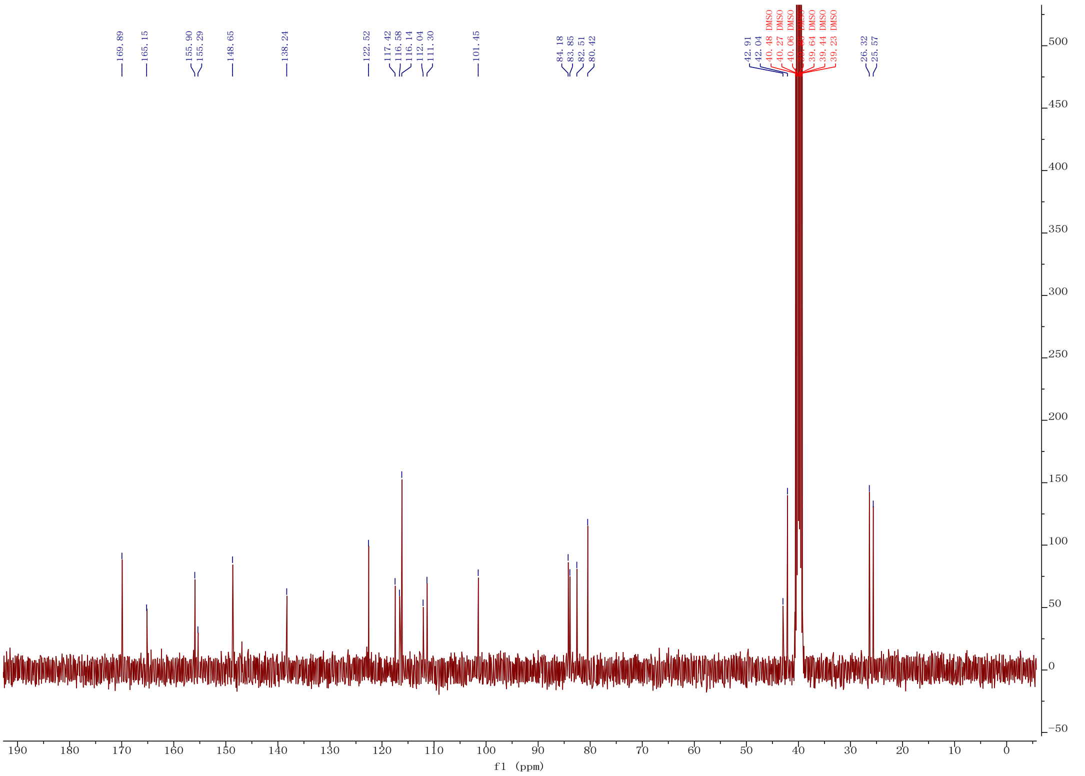

169.89
165.15
155.90
155.29
148.65
138.24
122.52
117.42
116.58
116.14
112.04
111.30
101.45
84.18
83.85
82.51
80.42
42.91
42.04
40.48 DMSO
40.27 DMSO
40.06 DMSO
39.64 DMSO
39.44 DMSO
39.23 DMSO
26.32
25.57
f1 (ppm)

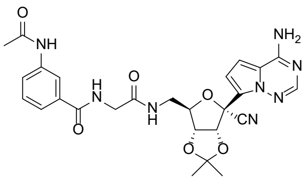

O
NH
H
N
O
N
H
O
O
NH2
N
N
N
CN
O
O
O

12h

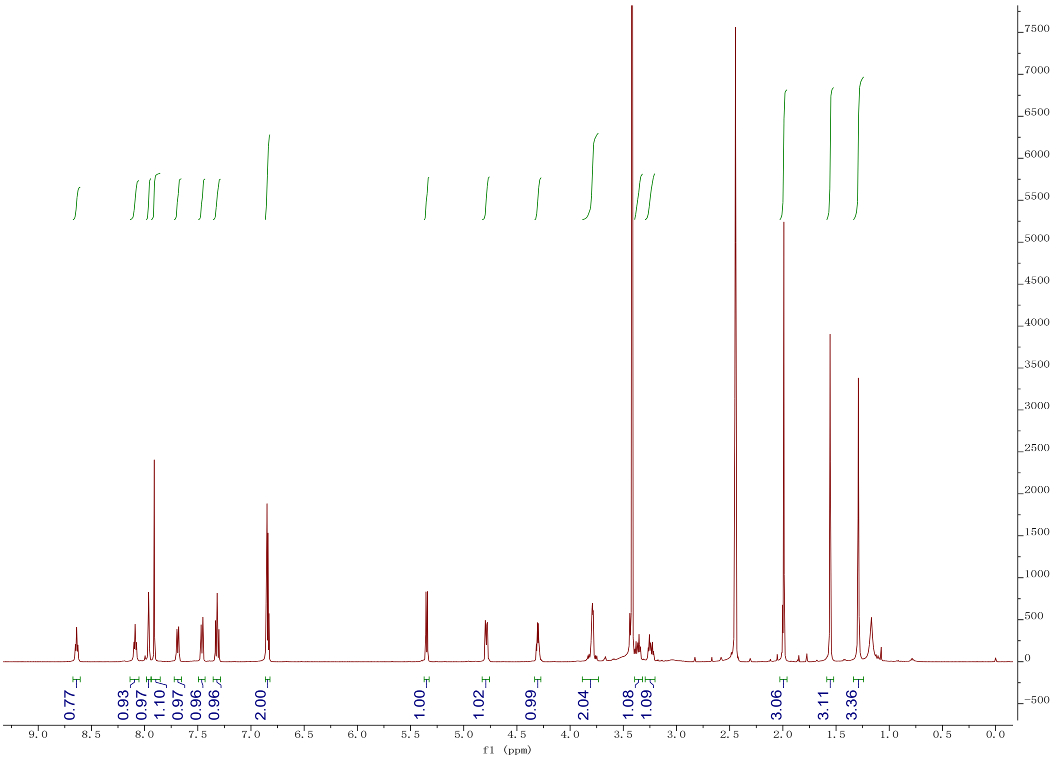

0.77
0.93
0.97
1.10
0.97
0.96
0.96
2.00
1.00
1.02
0.99
2.04
1.08
1.09
3.06
3.11
3.36
f1 (ppm)

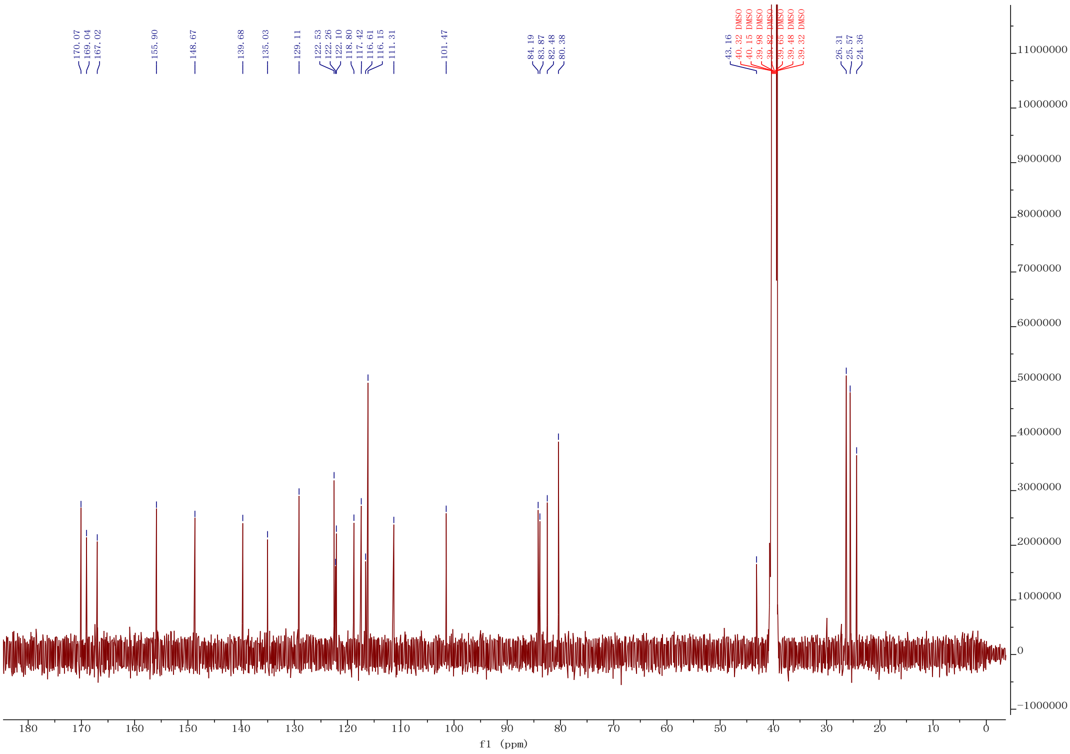

170.07
169.04
167.02
155.90
148.67
139.68
135.03
129.11
122.53
122.26
122.10
118.80
117.42
116.61
116.15
111.31
101.47
84.19
83.87
82.48
80.38
43.16
40.32 DMSO
40.15 DMSO
39.98 DMSO
39.82 DMSO
39.65 DMSO
39.48 DMSO
39.32 DMSO
26.31
25.57
24.36
f1 (ppm)

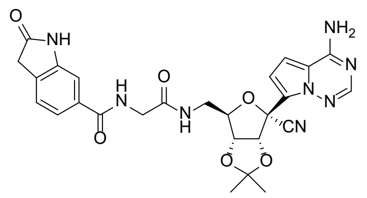
O
NH
H
N
O
N
H
O
NH2
N
N
N
CN
O
O
O

12i

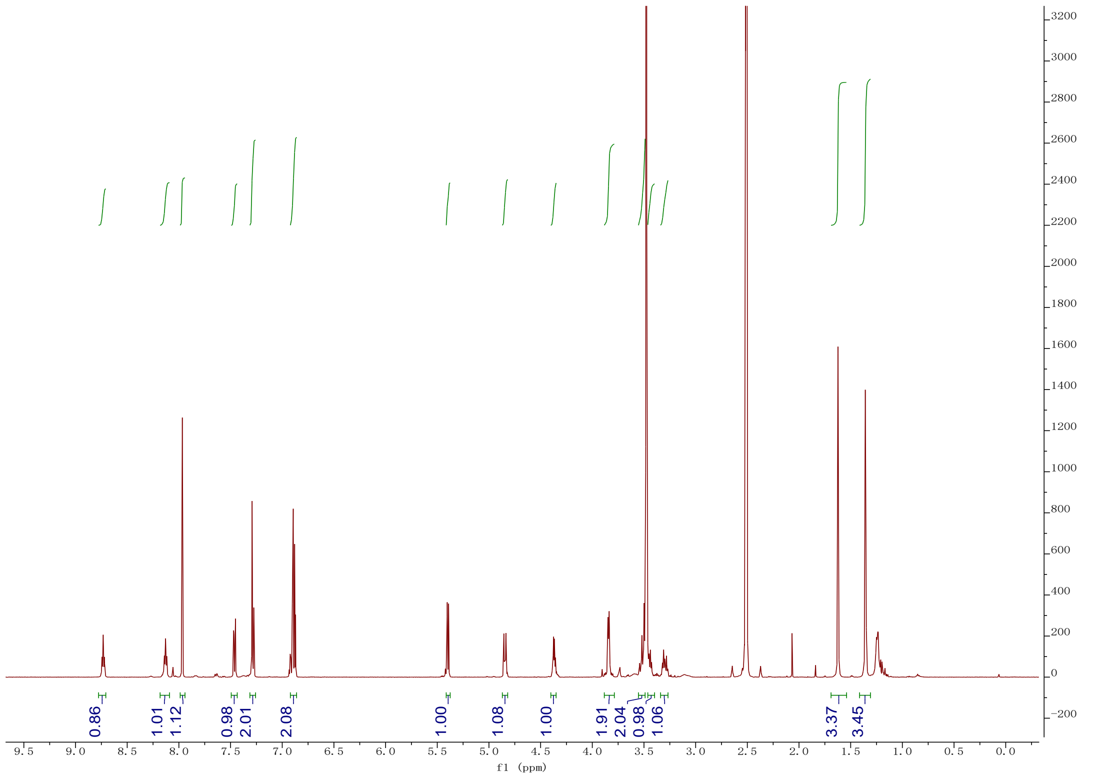
0.86
1.01
1.12
0.98
2.01
2.08
1.00
1.08
1.00
1.91
2.04
0.98
1.06
3.37
3.45
f1 (ppm)

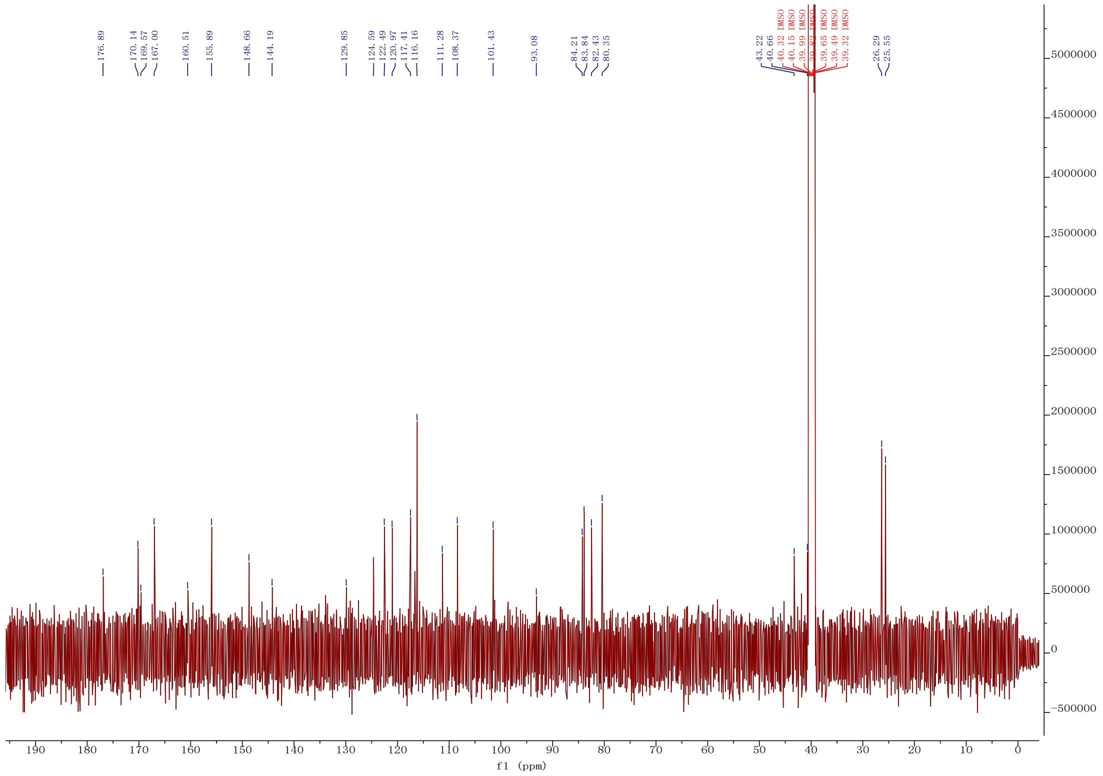
176.89
170.14
169.57
167.00
160.51
155.89
148.66
144.19
129.85
124.59
122.49
120.97
117.41
116.16
111.28
108.37
101.43
93.08
84.21
83.84
82.43
80.35
43.22
40.66
40.32 DMSO
40.15 DMSO
39.99 DMSO
39.82 DMSO
39.65 DMSO
39.49 DMSO
39.32 DMSO
26.29
25.55
f1 (ppm)

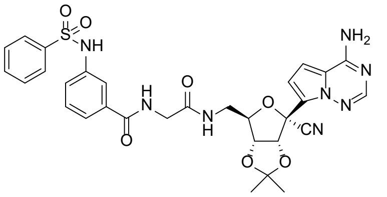

NH2
N
N
N
O
CN
O
O
H
N
O
N
H
O
NH
S
O
O

12j

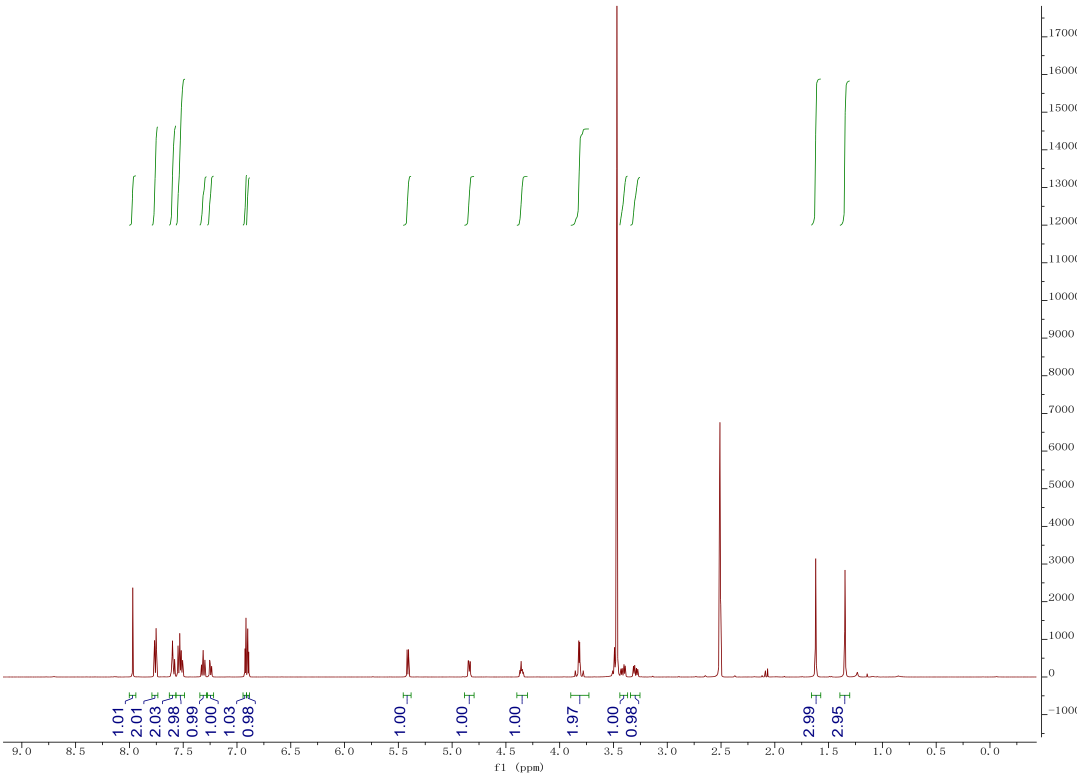

1.01
2.01
2.03
2.98
0.99
1.00
1.03
0.98
1.00
1.00
1.00
1.97
1.00
0.98
2.99
2.95
f1 (ppm)

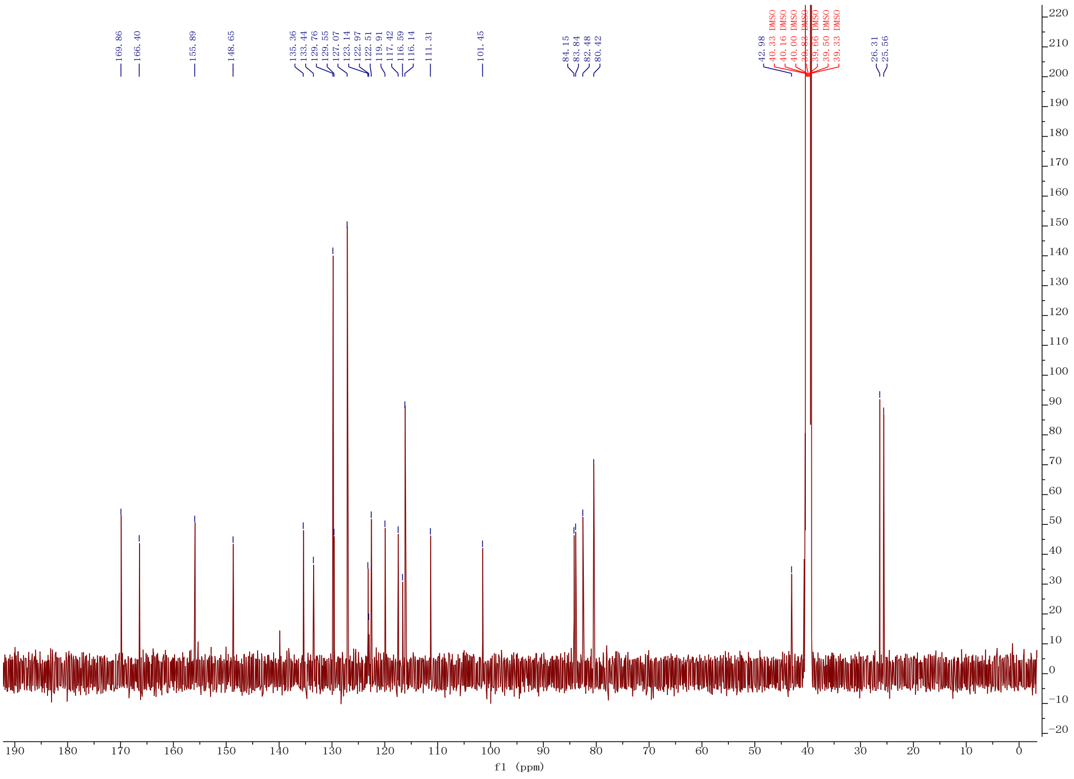

169.86
166.40
155.89
148.65
135.36
133.44
129.76
129.55
127.07
123.14
122.97
122.51
119.91
117.42
116.59
116.14
111.31
101.45
84.15
83.84
82.48
80.42
42.98
40.33 DMSO
40.16 DMSO
40.00 DMSO
39.50 DMSO
39.33 DMSO
26.31
25.56
f1 (ppm)

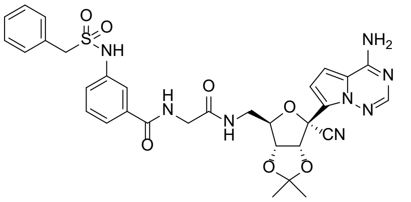

12k

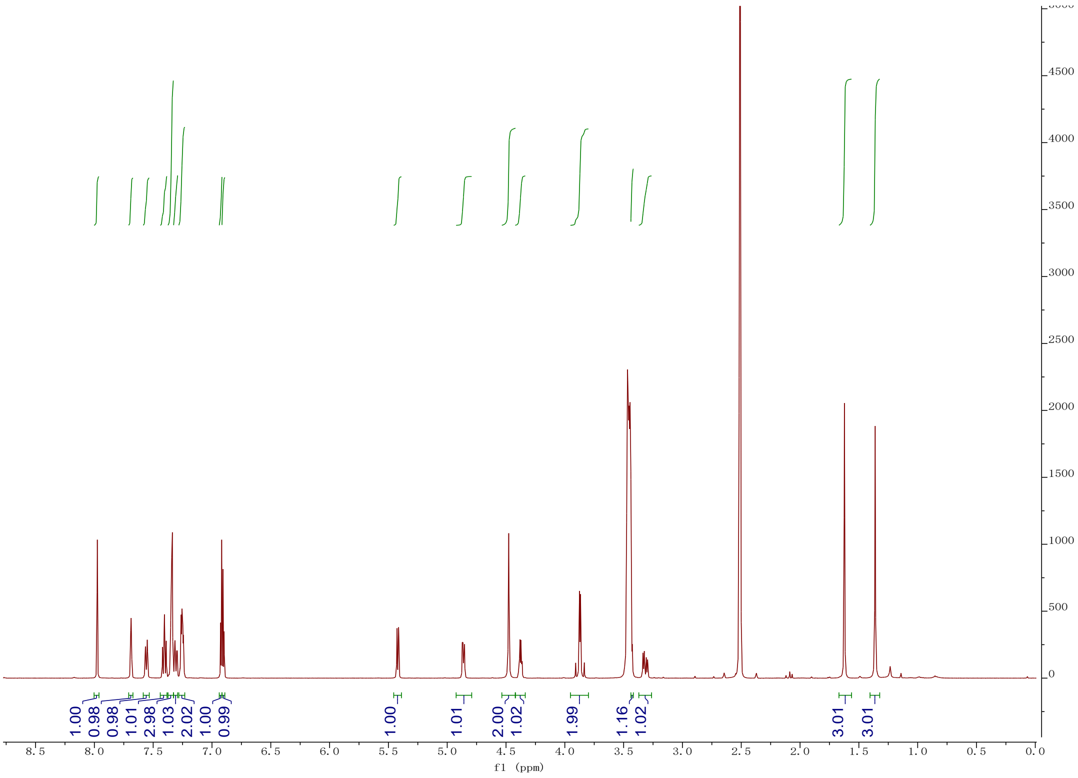

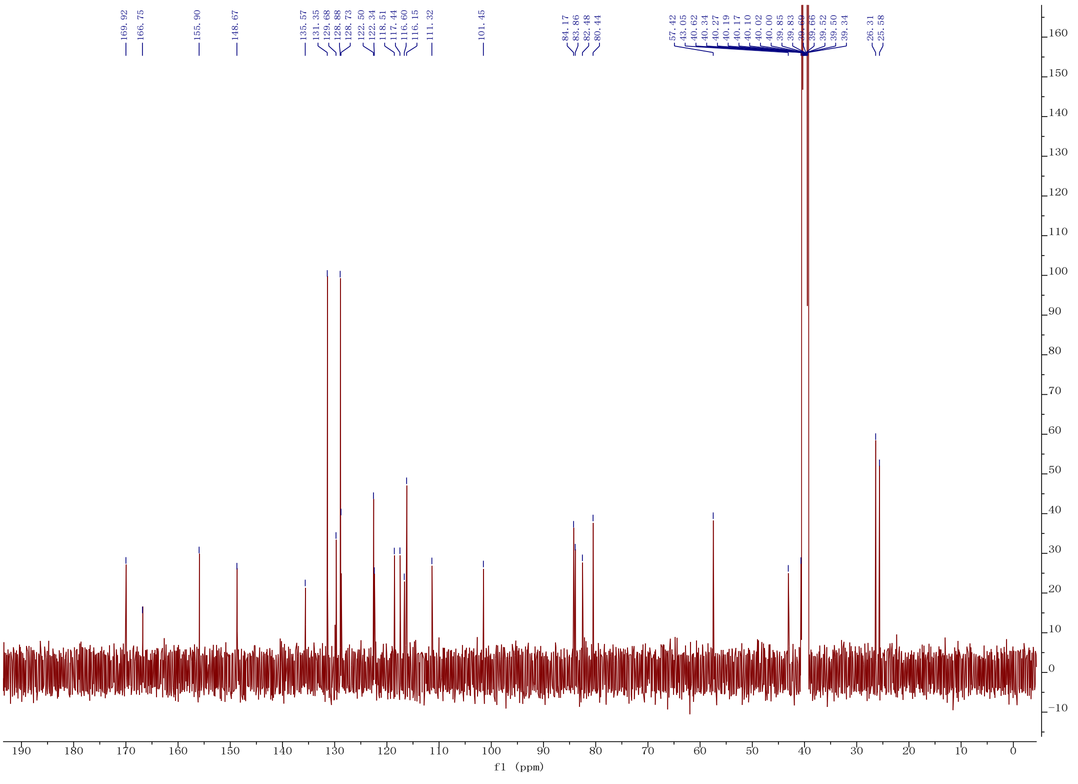

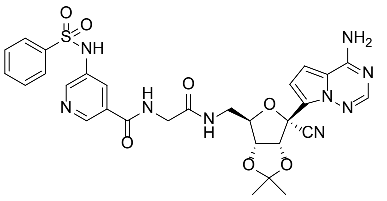
NH2
N
N
N
O
CN
O
O
N
H
O
H
N
N
O
NH
S
O
O

12l

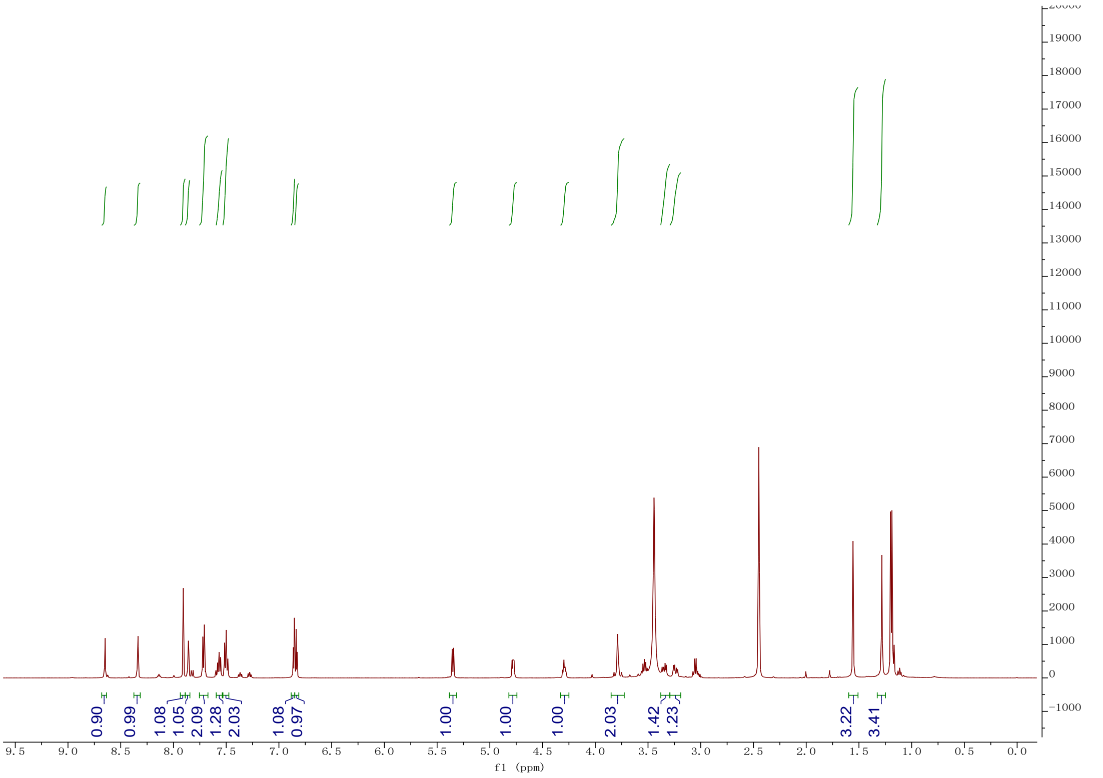
0.90
0.99
1.08
1.05
2.09
1.28
2.03
1.08
0.97
1.00
1.00
1.00
2.03
1.42
1.23
3.22
3.41
f1 (ppm)

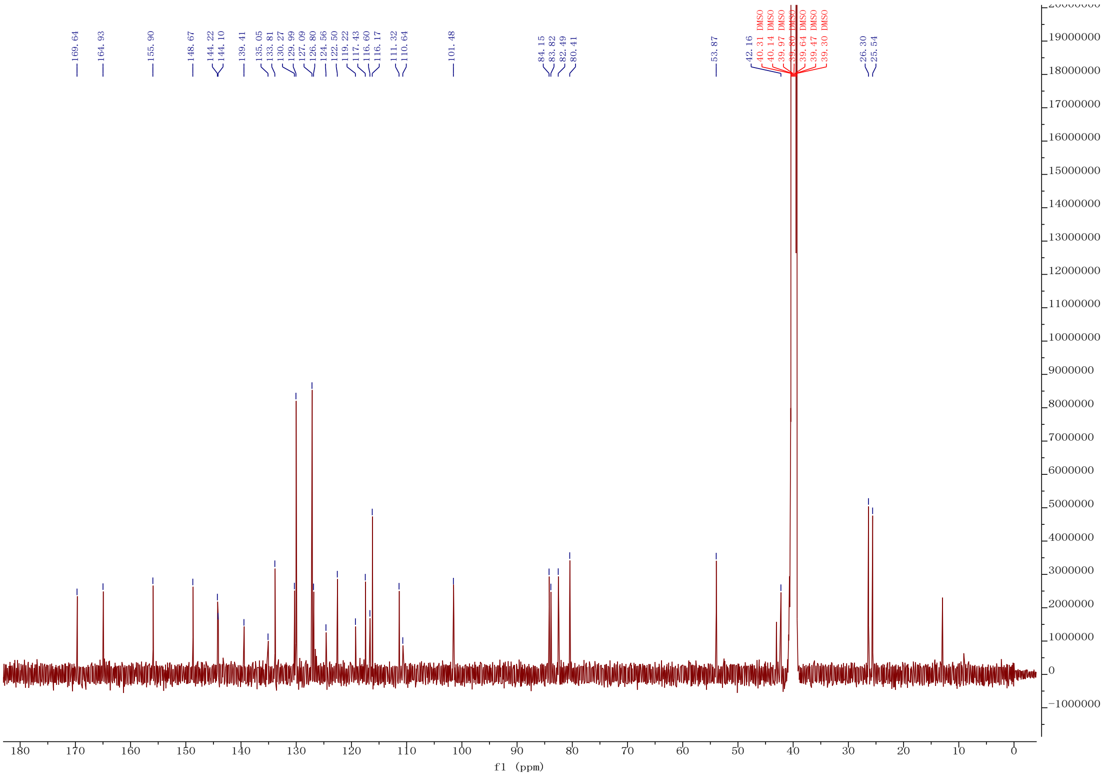
169.64
164.93
155.90
148.67
144.22
144.10
139.41
135.05
133.81
130.27
129.99
127.09
126.80
124.56
122.50
119.22
117.43
116.60
116.17
111.32
110.64
101.48
84.15
83.82
82.49
80.41
53.87
42.16
40.31 DMSO
40.14 DMSO
39.97 DMSO
[illegible] DMSO
[illegible] DMSO
39.47 DMSO
39.30 DMSO
26.30
25.54
f1 (ppm)

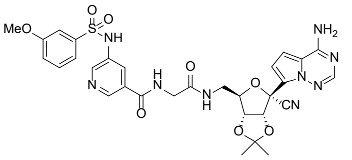

12m

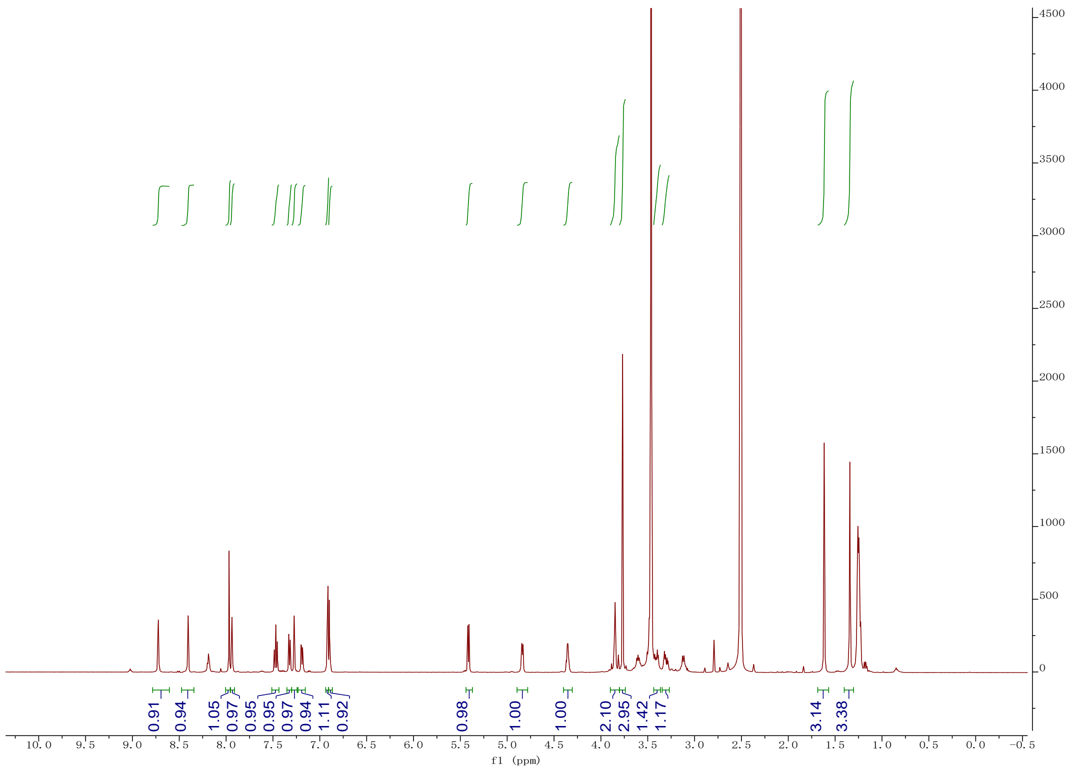

0.91
0.94
1.05
0.97
0.95
0.95
0.97
0.94
1.11
0.92
0.98
1.00
1.00
2.10
2.95
1.42
1.17
3.14
3.38
f1 (ppm)

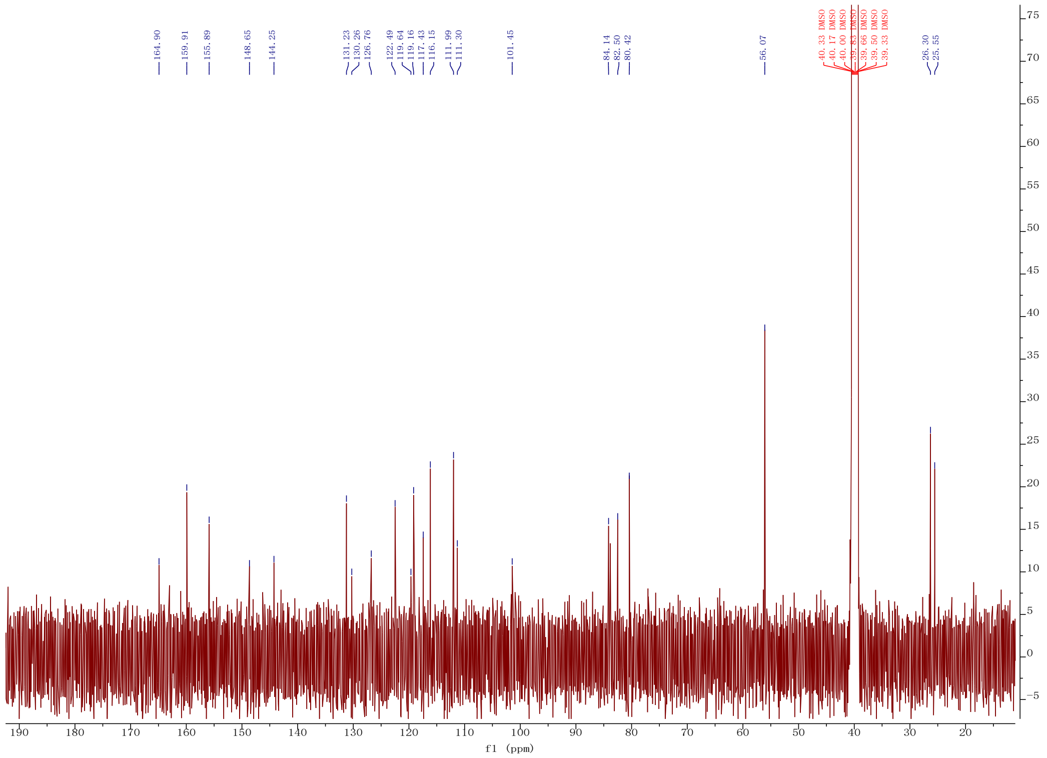

164.90
159.91
155.89
148.65
144.25
131.23
130.26
126.76
122.49
119.64
119.16
117.43
116.15
111.99
111.30
101.45
84.14
82.50
80.42
56.07
26.30
25.55
f1 (ppm)

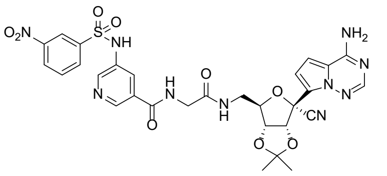

12n

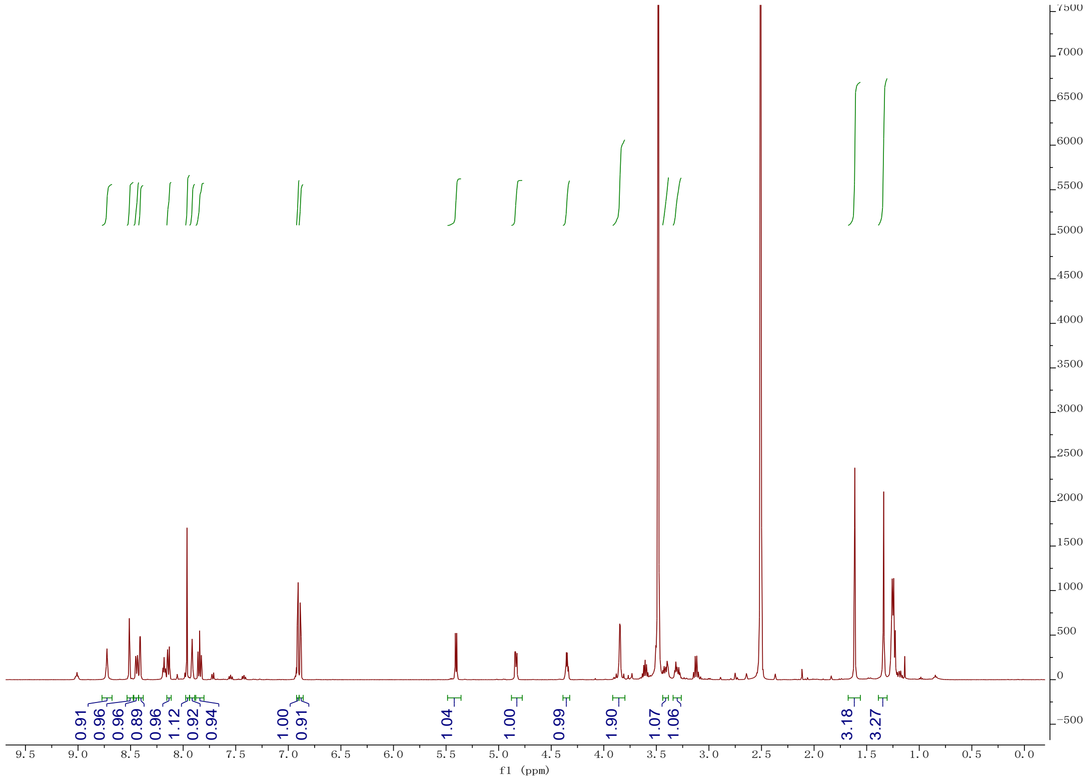

0.91
0.96
0.96
0.89
0.96
1.12
0.92
0.94
1.00
0.91
1.04
1.00
0.99
1.90
1.07
1.06
3.18
3.27
f1 (ppm)

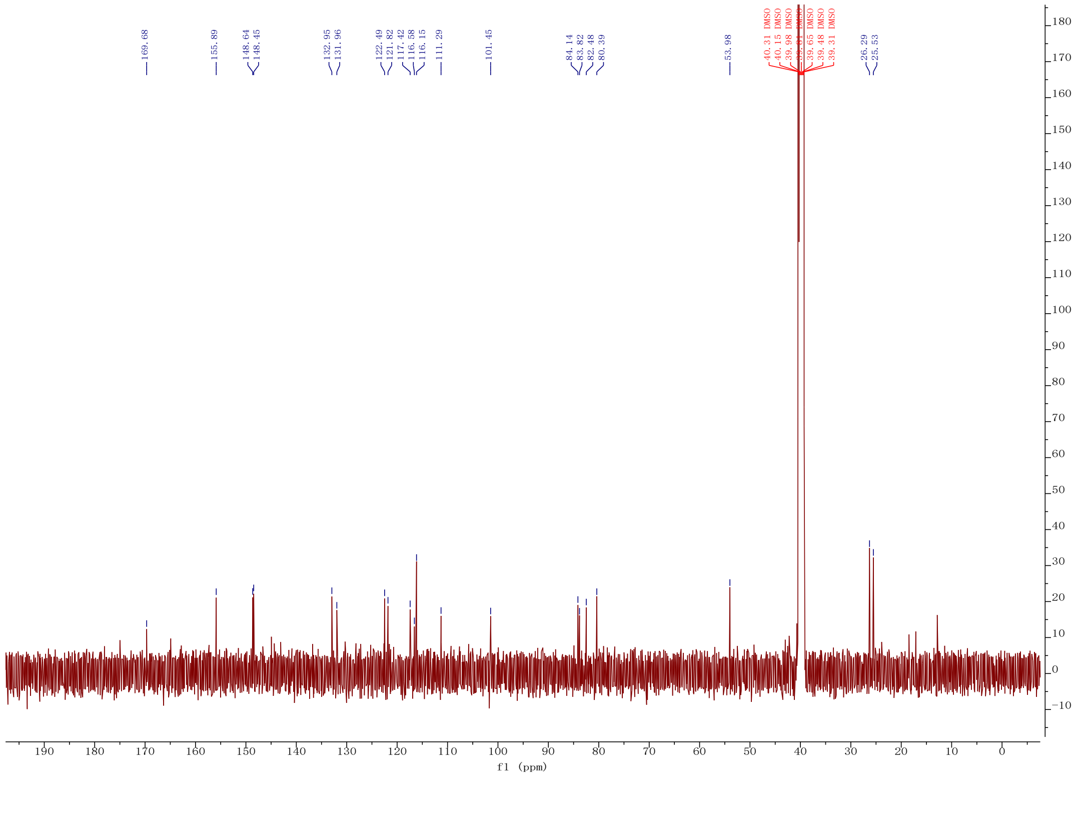

169.68
155.89
148.64
148.45
132.95
131.96
122.49
121.82
117.42
116.58
116.15
111.29
101.45
84.14
83.82
82.48
80.39
53.98
26.29
25.53
f1 (ppm)

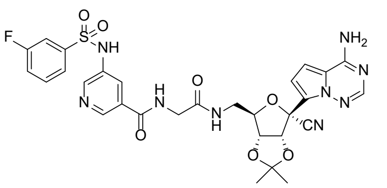
F
O
S
O
NH
N
H
N
O
N
H
O
NH2
N
N
N
CN
O
O

12o

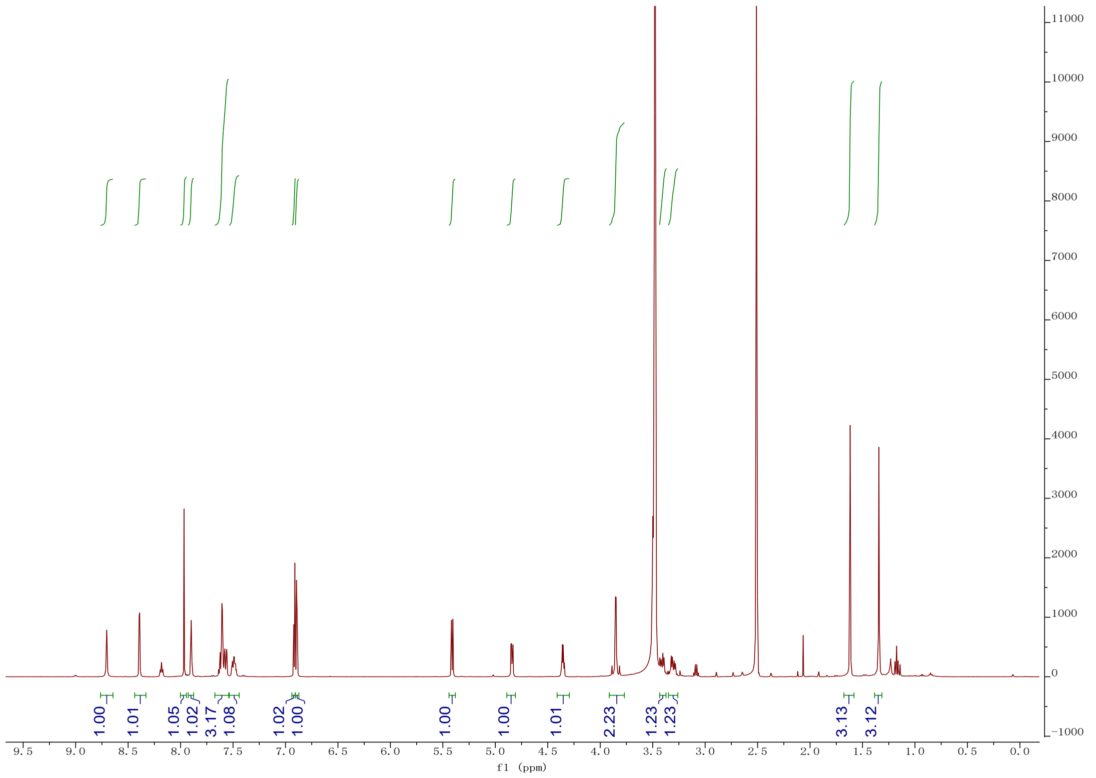
1.00
1.01
1.05
1.02
3.17
1.08
1.02
1.00
1.00
1.00
1.01
2.23
1.23
1.23
3.13
3.12
f1 (ppm)

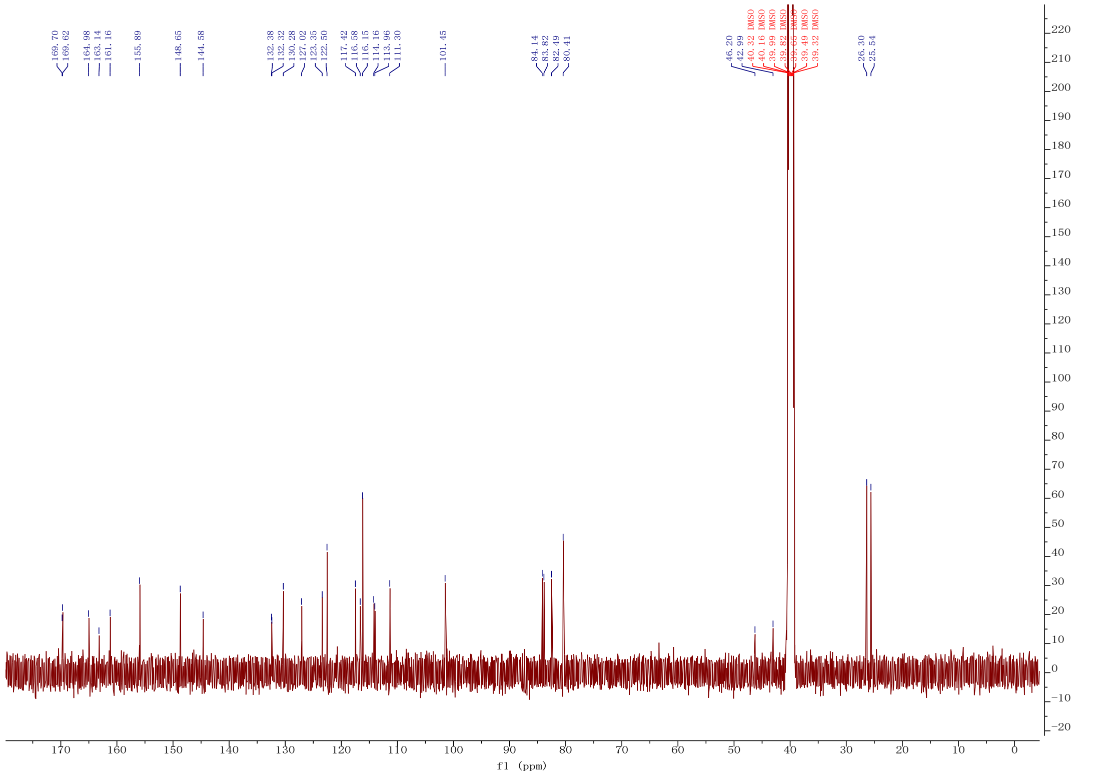
169.70
169.62
164.98
163.14
161.16
155.89
148.65
144.58
132.38
132.32
130.28
127.02
123.55
122.50
117.42
116.58
116.15
114.16
113.96
111.30
101.45
84.14
83.82
82.49
80.41
46.20
42.99
40.32 DMSO
40.16 DMSO
39.99 DMSO
39.82 DMSO
39.65 DMSO
39.49 DMSO
39.32 DMSO
26.30
25.54
f1 (ppm)

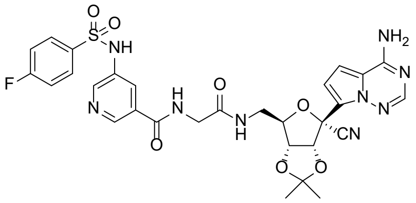

O
O
S
NH
F
N
H
N
O
O
N
H
O
NH2
N
N
N
CN
O
O

12p

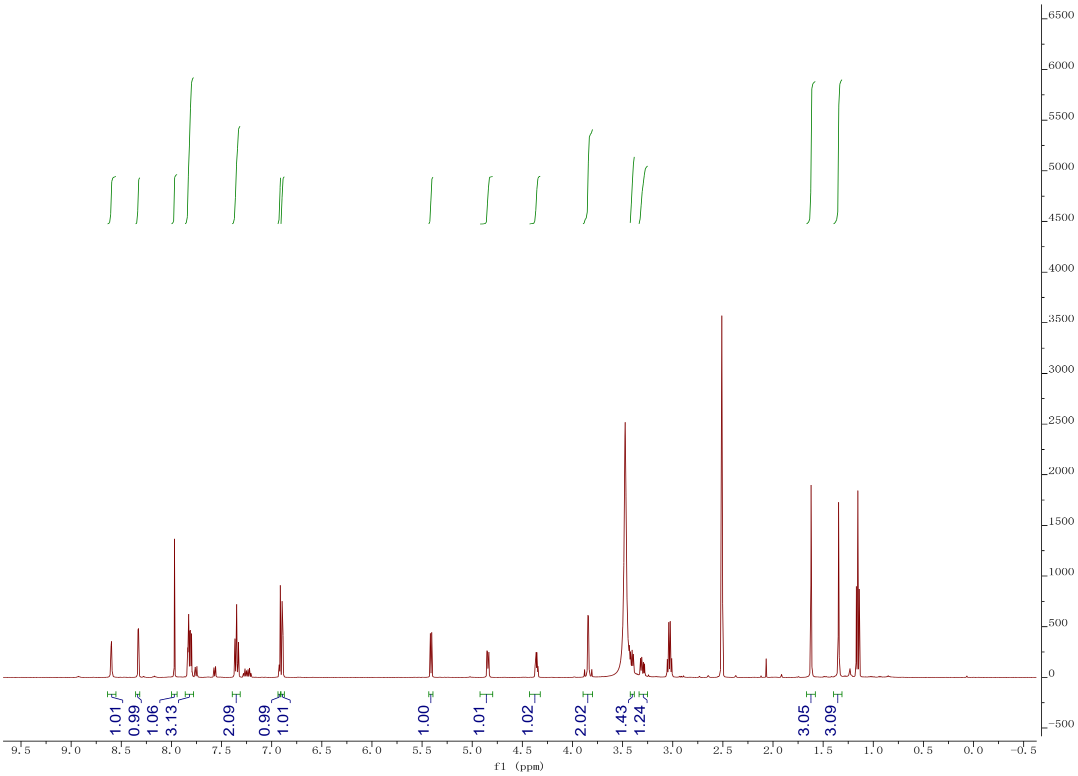

1.01
0.99
1.06
3.13
2.09
0.99
1.01
1.00
1.01
1.02
2.02
1.43
1.24
3.05
3.09
f1 (ppm)

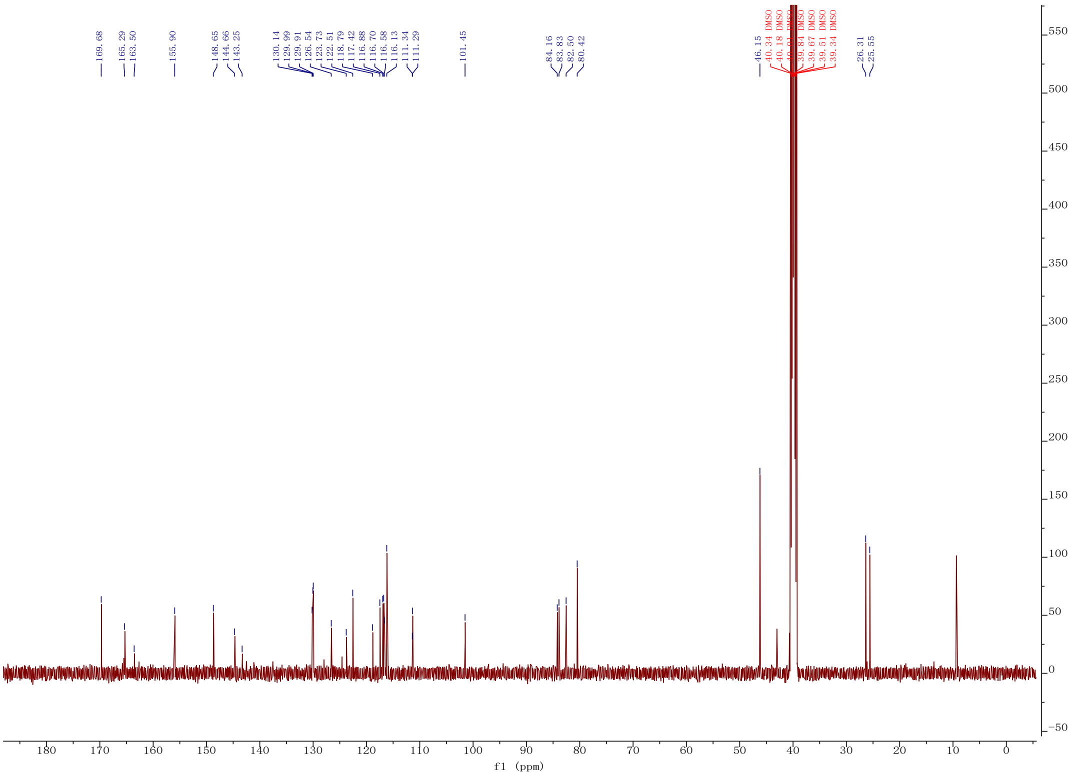

169.68
165.29
163.50
155.90
148.65
144.66
143.25
130.14
129.99
129.91
126.54
123.73
122.51
118.79
117.42
116.88
116.70
116.58
116.13
111.34
111.29
101.45
84.16
83.83
82.50
80.42
46.15
40.34 DMSO
40.18 DMSO
39.67 DMSO
39.51 DMSO
39.34 DMSO
26.31
25.55
f1 (ppm)

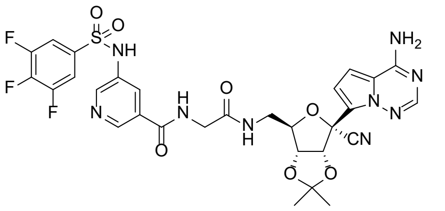
F
F
F
O
O
S
NH
N
H
N
O
O
N
H
O
NH2
N
N
N
CN
O
O

12q

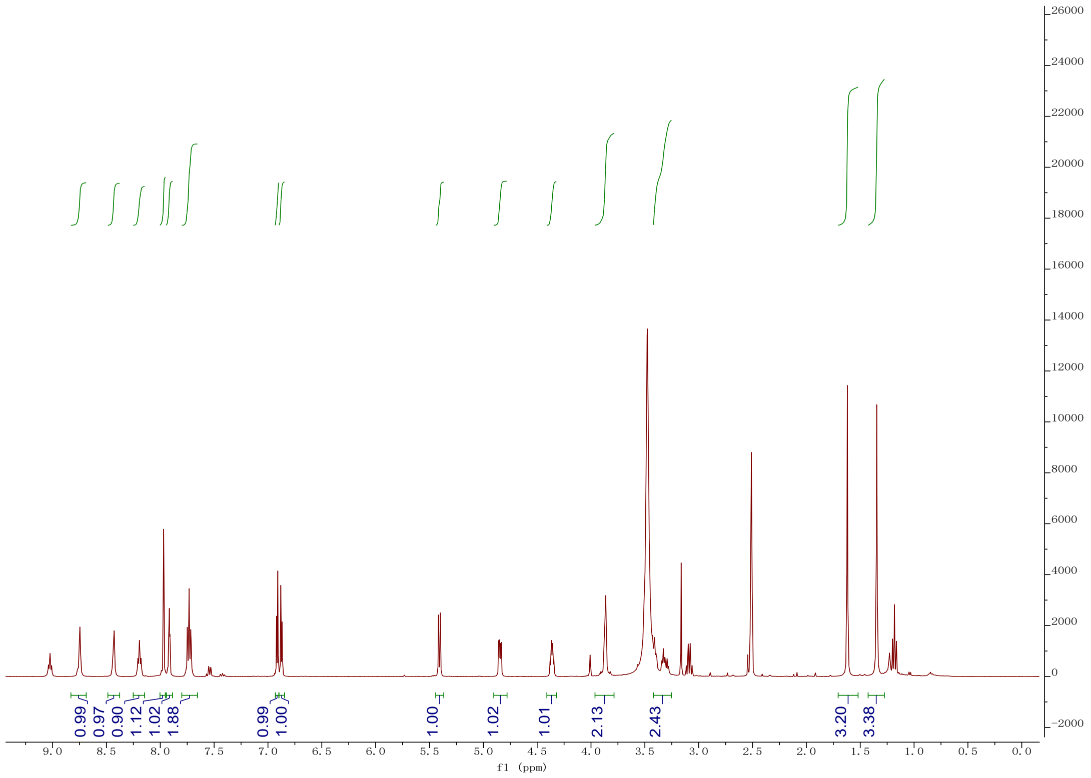
0.99
0.97
0.90
1.12
1.02
1.88
0.99
1.00
1.00
1.02
1.01
2.13
2.43
3.20
3.38
f1 (ppm)

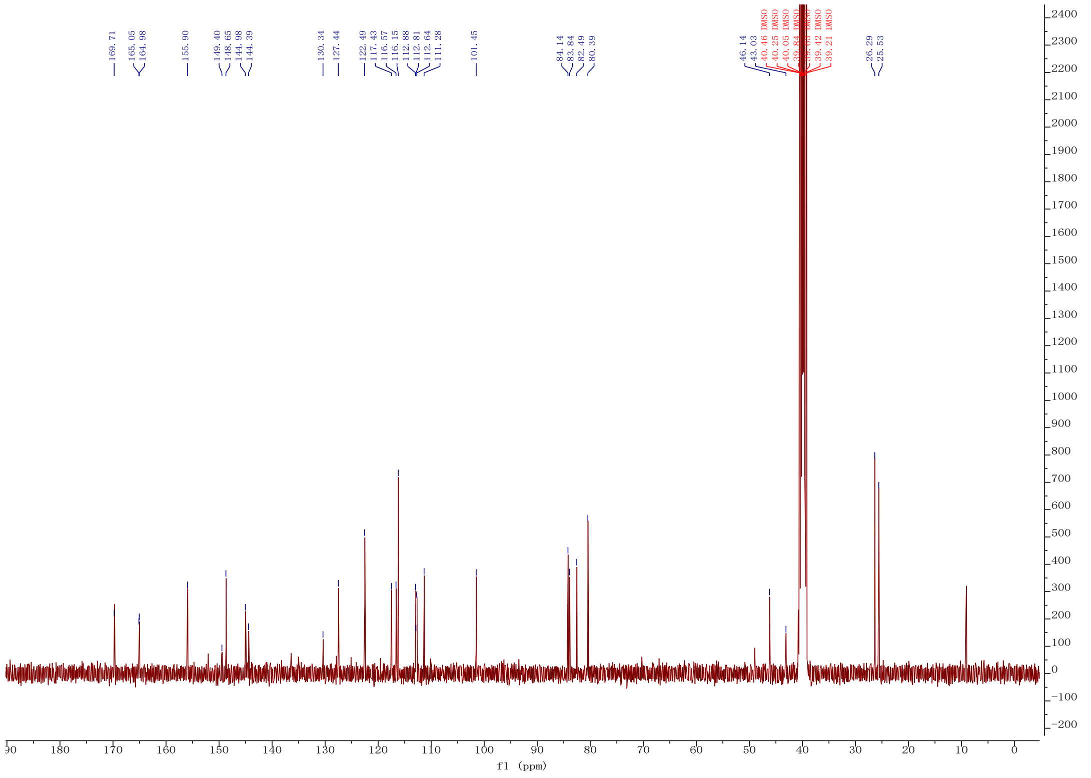
169.71
165.05
164.98
155.90
149.40
148.65
144.98
144.39
130.34
127.44
122.49
117.43
116.57
116.15
112.88
112.81
112.64
111.28
101.45
84.14
83.84
82.49
80.39
46.14
43.03
40.46 DMSO
40.25 DMSO
40.05 DMSO
39.42 DMSO
39.21 DMSO
26.29
25.53
f1 (ppm)

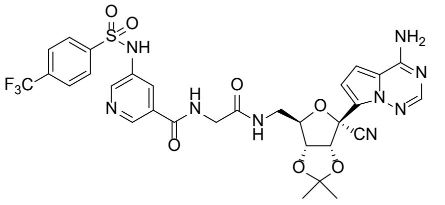

12r

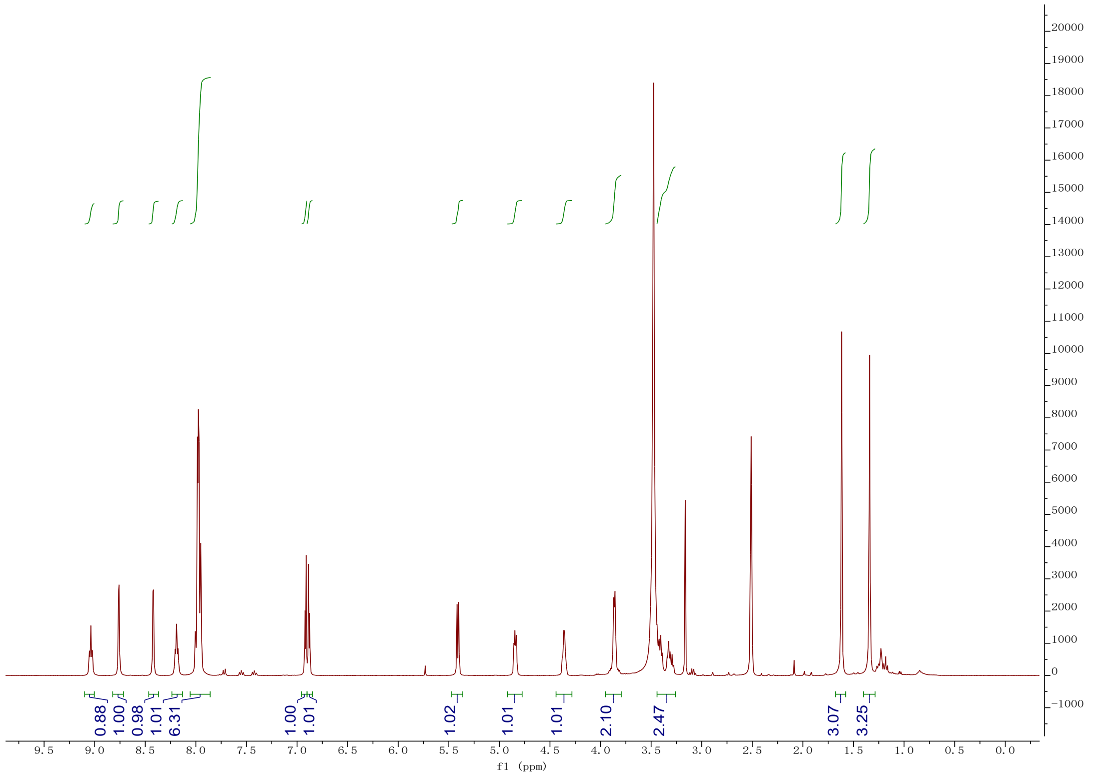

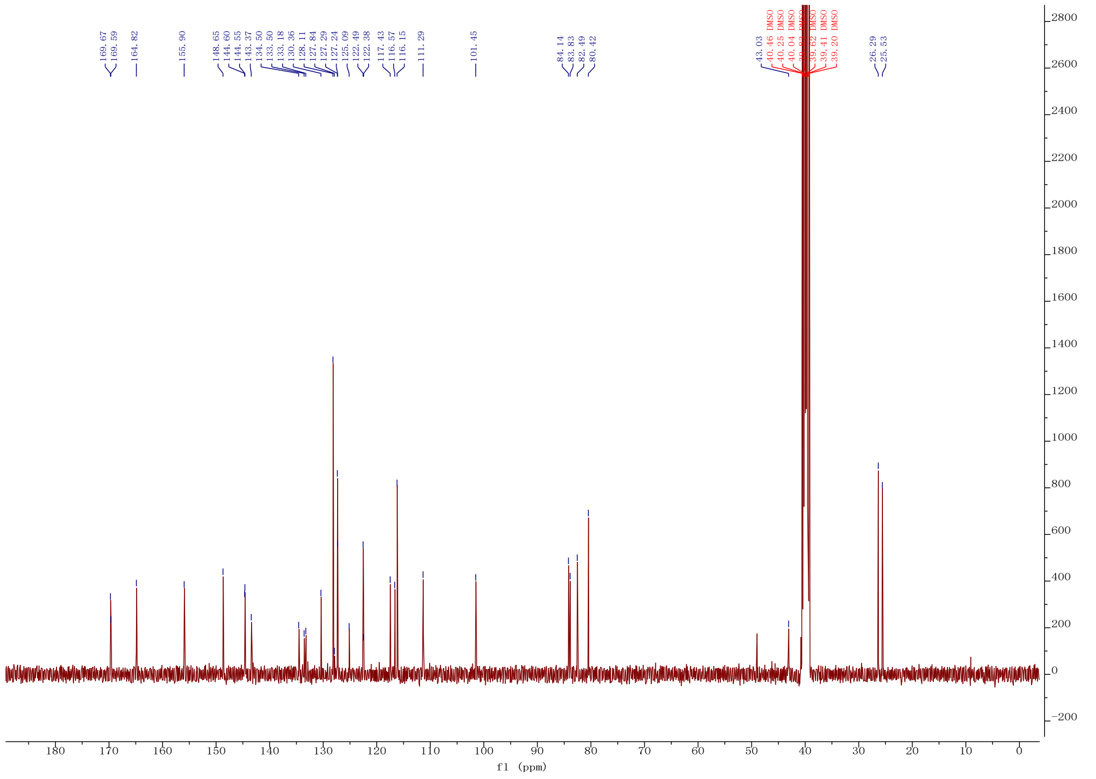

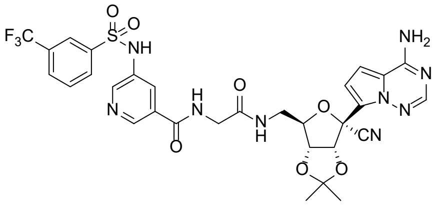
F3C
O
S
O
NH
N
H
N
O
O
N
H
O
NH2
N
N
N
CN
O
O

12s

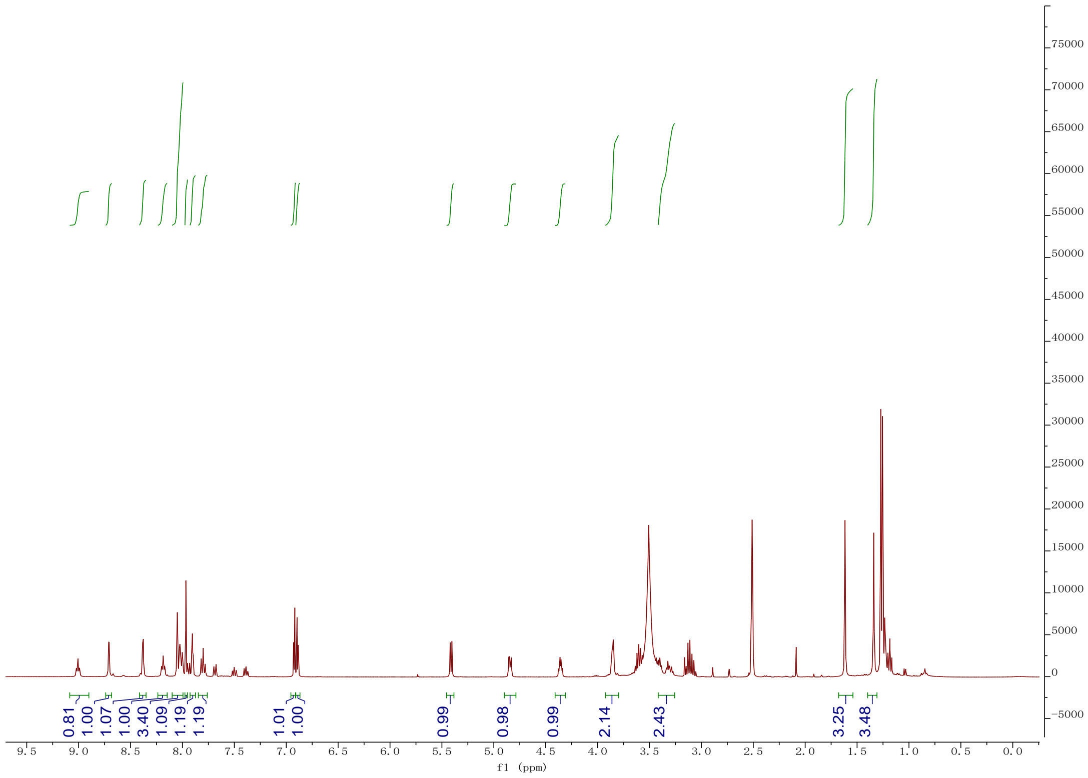
0.81
1.00
1.07
1.00
3.40
1.09
1.19
1.19
1.01
1.00
0.99
0.98
0.99
2.14
2.43
3.25
3.48
f1 (ppm)

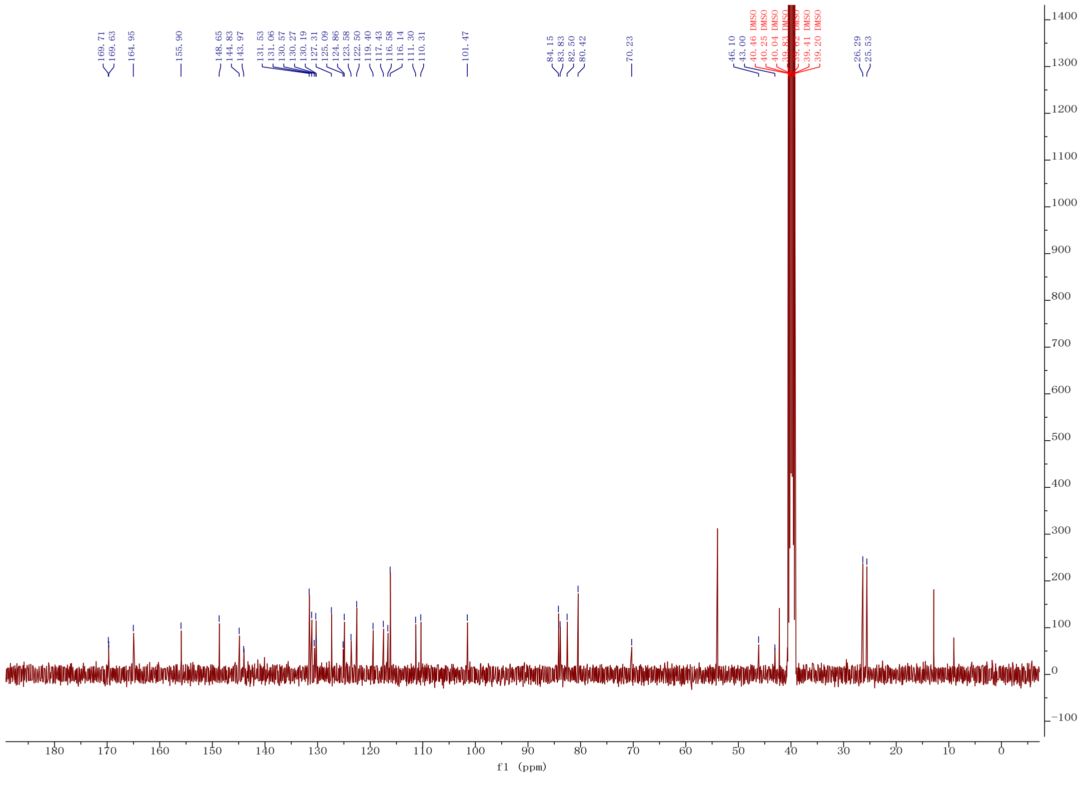
169.71
169.63
164.95
155.90
148.65
144.83
143.97
131.53
131.06
130.57
130.27
130.19
127.31
125.09
124.86
123.58
122.50
119.40
117.43
116.58
116.14
111.30
110.31
101.47
84.15
83.83
82.50
80.42
70.23
46.10
43.00
40.46 DMSO
40.25 DMSO
40.04 DMSO
39.83 DMSO
39.62 DMSO
39.41 DMSO
39.20 DMSO
26.29
25.53
f1 (ppm)

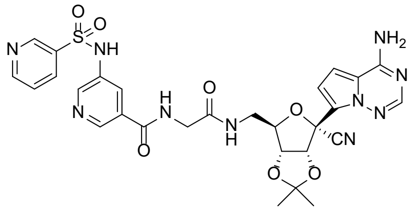
NH2
N
N
O
NH
O
S
N
N
H
O
N
H
O
N
N
O
CN
O
O

12t

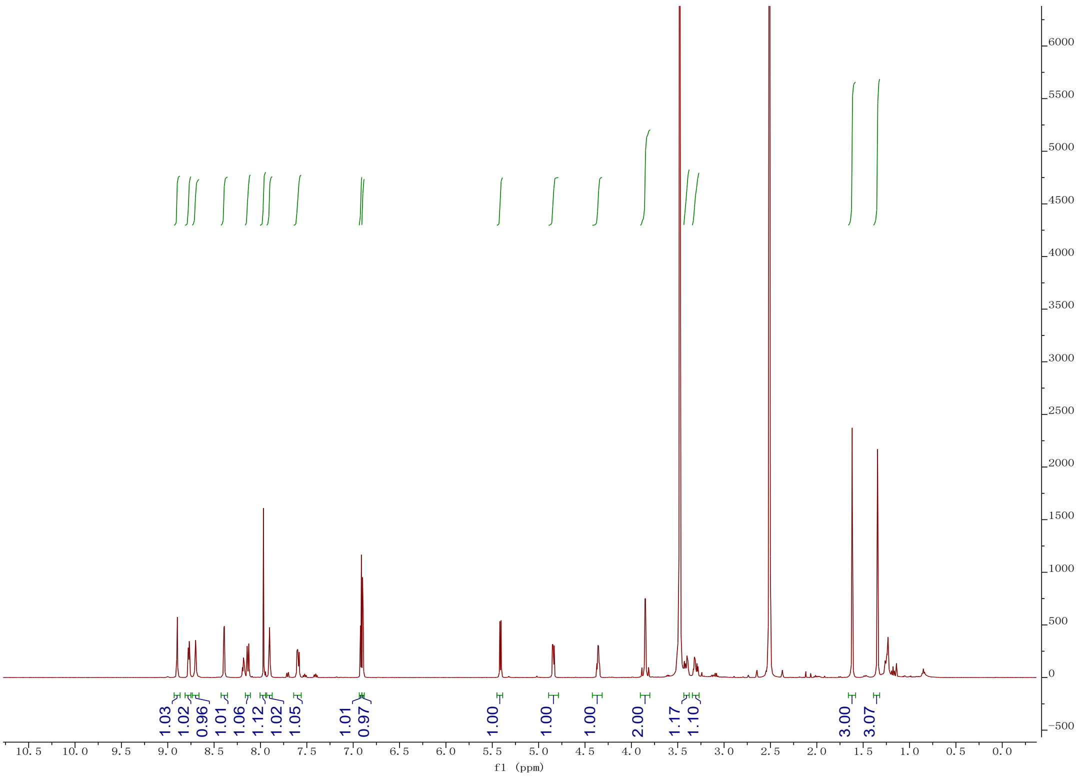
1.03
1.02
0.96
1.01
1.06
1.12
1.02
1.05
1.01
0.97
1.00
1.00
1.00
2.00
1.17
1.10
3.00
3.07
f1 (ppm)

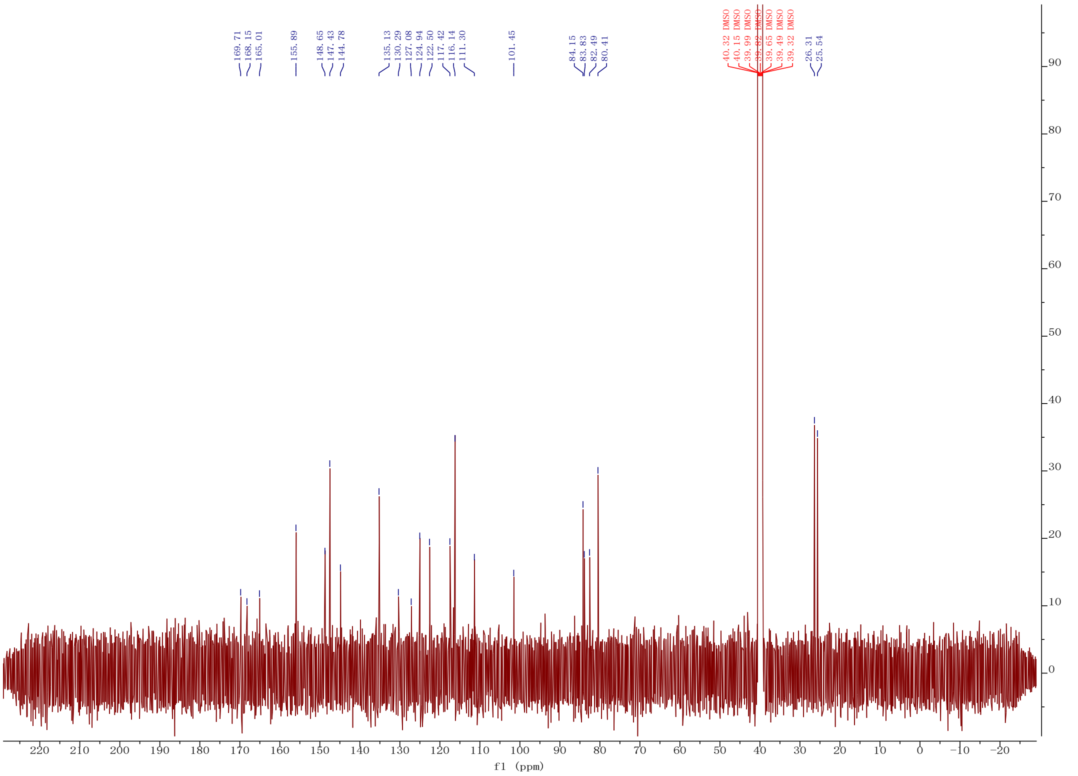
169.71
168.15
165.01
155.89
148.65
147.43
144.78
135.13
130.29
127.08
124.94
122.50
117.42
116.14
111.30
101.45
84.15
83.83
82.49
80.41
40.32 DMSO
40.15 DMSO
39.99 DMSO
39.65 DMSO
39.49 DMSO
39.32 DMSO
26.31
25.54
f1 (ppm)

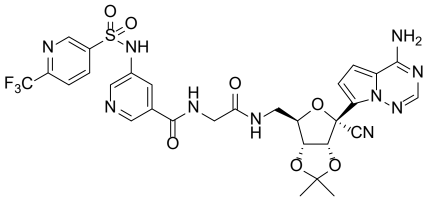

12u

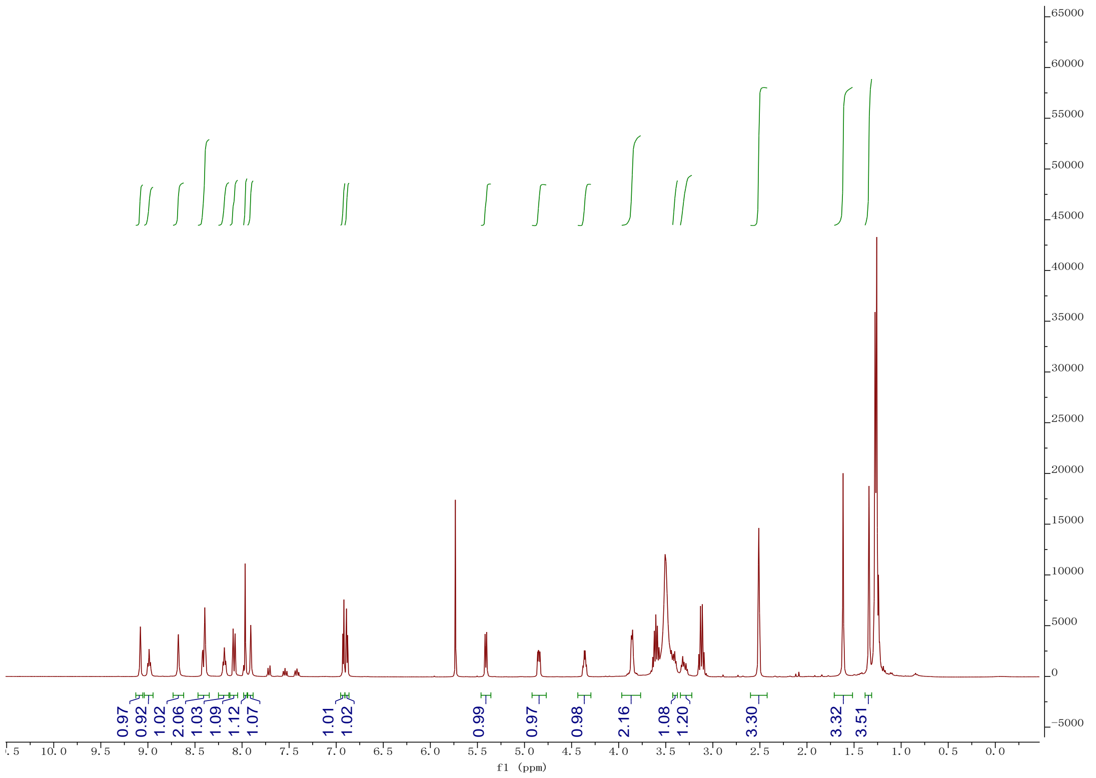

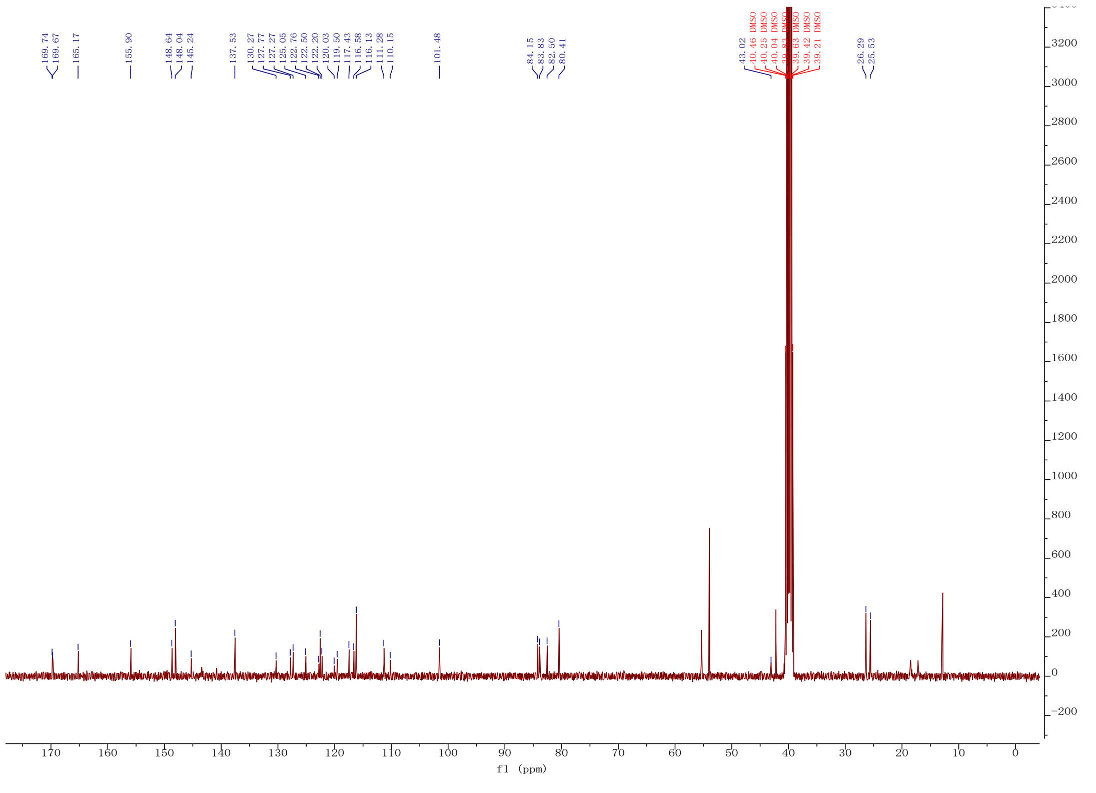

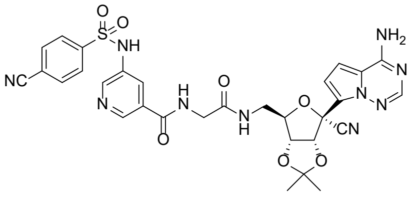

12v

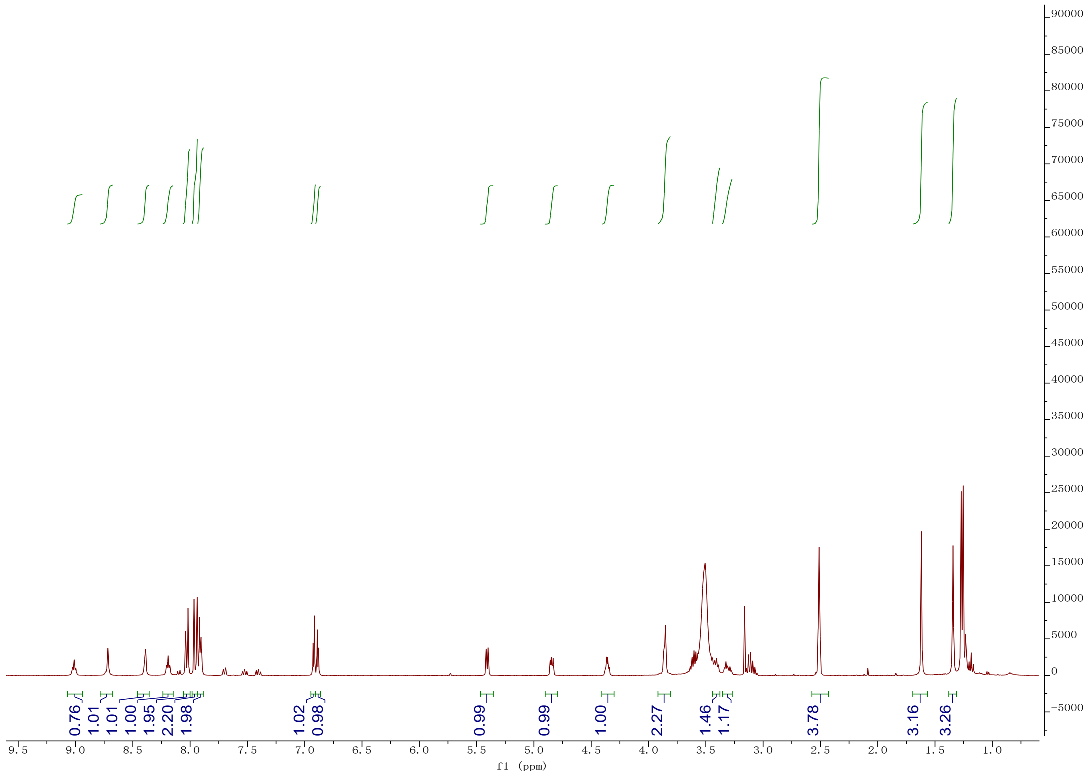
0.76
1.01
1.00
1.95
2.20
1.98
1.02
0.98
0.99
0.99
1.00
2.27
1.46
1.17
3.78
3.16
3.26
f1 (ppm)

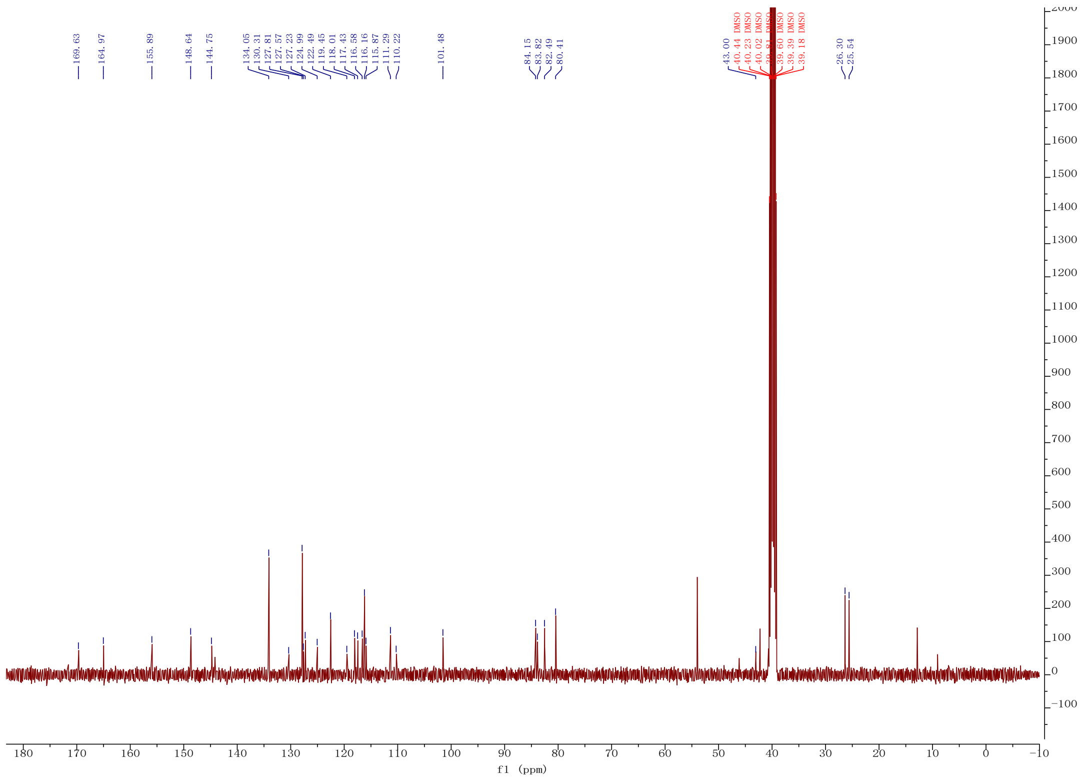
169.63
164.97
155.89
148.64
144.75
134.05
130.31
127.81
127.57
127.23
124.99
122.49
119.45
118.01
117.43
116.58
116.16
115.87
111.29
110.22
101.48
84.15
83.82
82.49
80.41
43.00
40.44 DMSO
40.23 DMSO
40.02 DMSO
39.81 DMSO
39.60 DMSO
39.39 DMSO
39.18 DMSO
26.30
25.54
f1 (ppm)

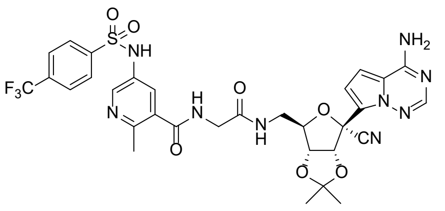

12w

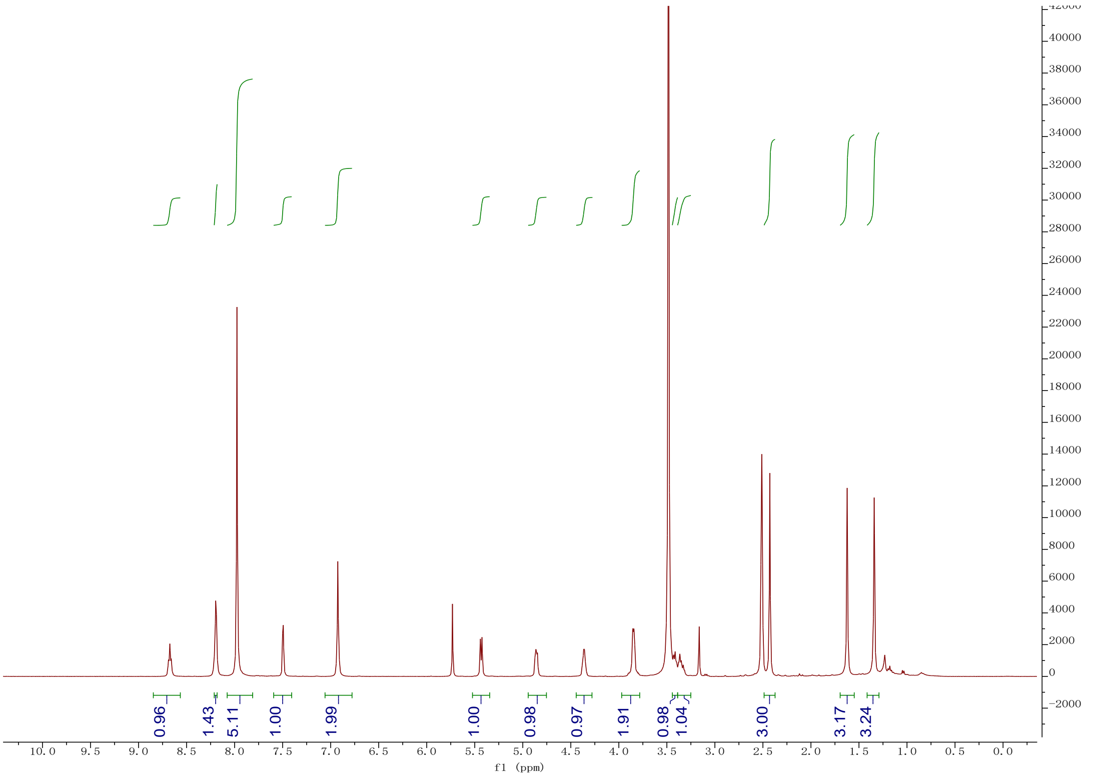
0.96
1.43
5.11
1.00
1.99
1.00
0.98
0.97
1.91
0.98
1.04
3.00
3.17
3.24
f1 (ppm)

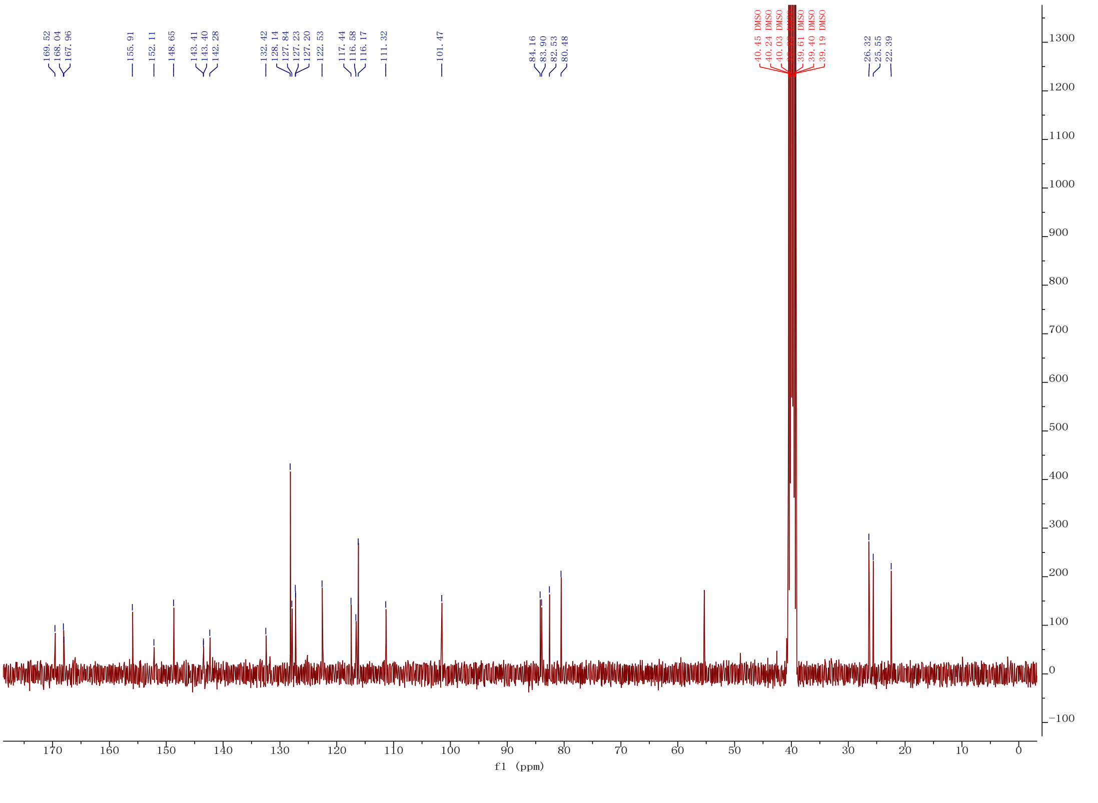
169.52
168.04
167.96
155.91
152.11
148.65
143.41
143.40
142.28
132.42
128.14
127.84
127.23
127.20
122.53
117.44
116.58
116.17
111.32
101.47
84.16
83.90
82.53
80.48
40.45 DMSO
40.24 DMSO
40.03 DMSO
39.61 DMSO
39.40 DMSO
39.19 DMSO
26.32
25.55
22.39
f1 (ppm)

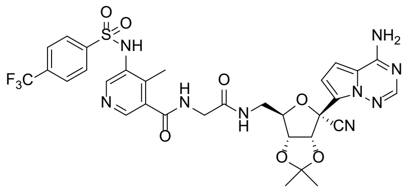
NH
F3C
N
H
O
O
N
H
O
NH2
N
N
N
CN
O
O

12x

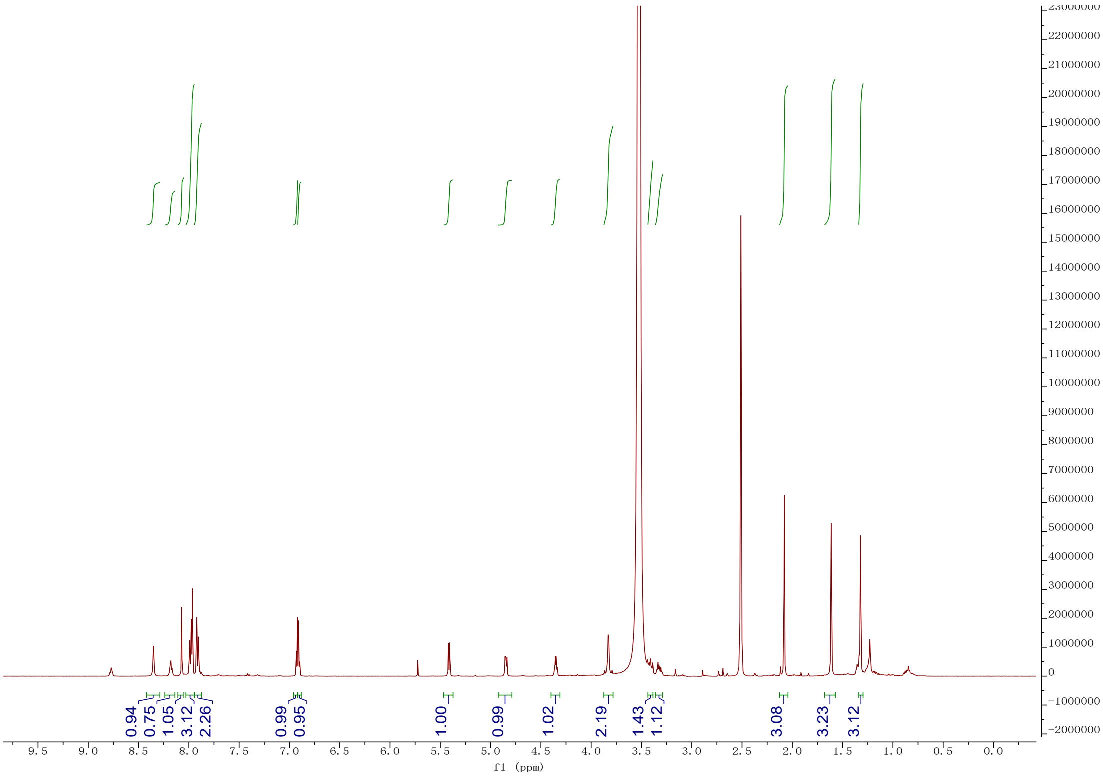
0.94
0.75
1.05
3.12
2.26
0.99
0.95
1.00
0.99
1.02
2.19
1.43
1.12
3.08
3.23
3.12
f1 (ppm)

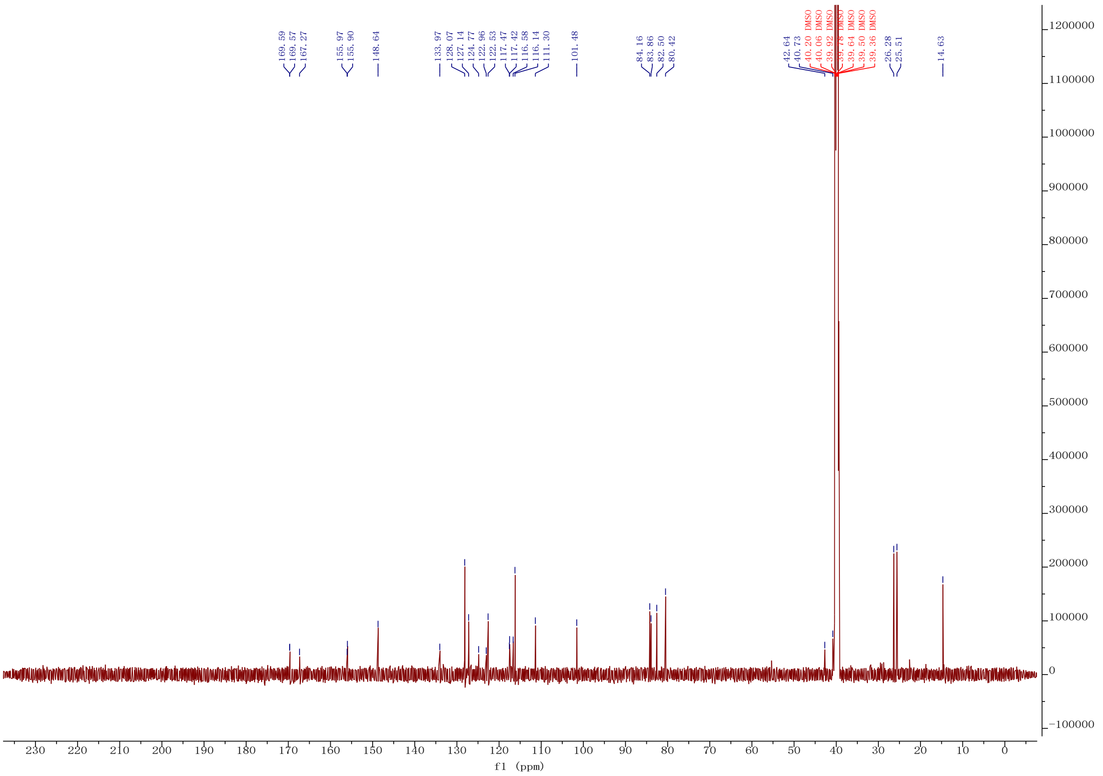
169.59
169.57
167.27
155.97
155.90
148.64
133.97
128.07
127.14
124.77
122.96
122.53
117.47
117.42
116.58
116.14
111.30
101.48
84.16
83.86
82.50
80.42
42.64
40.73
40.20 DMSO
40.06 DMSO
39.92 DMSO
39.78 DMSO
39.64 DMSO
39.50 DMSO
39.36 DMSO
26.28
25.51
14.63
f1 (ppm)

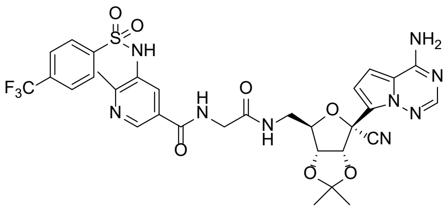
NH2
N
N
N
O
S
NH
F3C
N
H
N
O
O
N
H
O
CN
O
O

12y

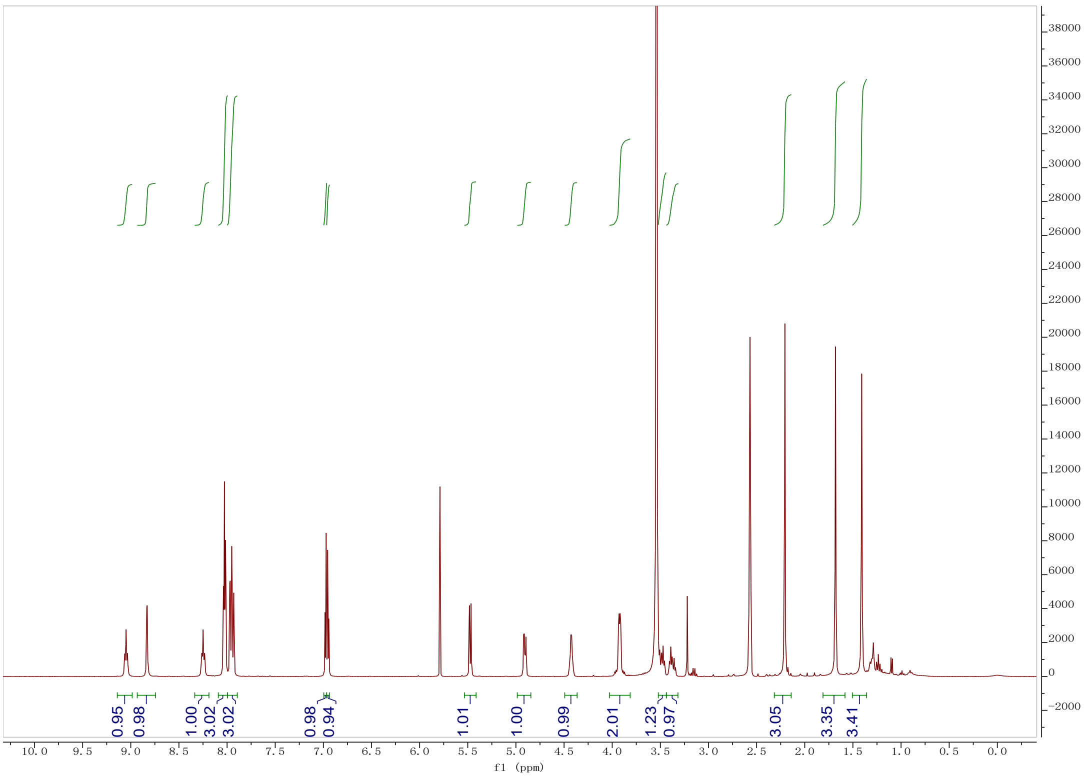
0.95
0.98
1.00
3.02
3.02
0.98
0.94
1.01
1.00
0.99
2.01
1.23
0.97
3.05
3.35
3.41
f1 (ppm)

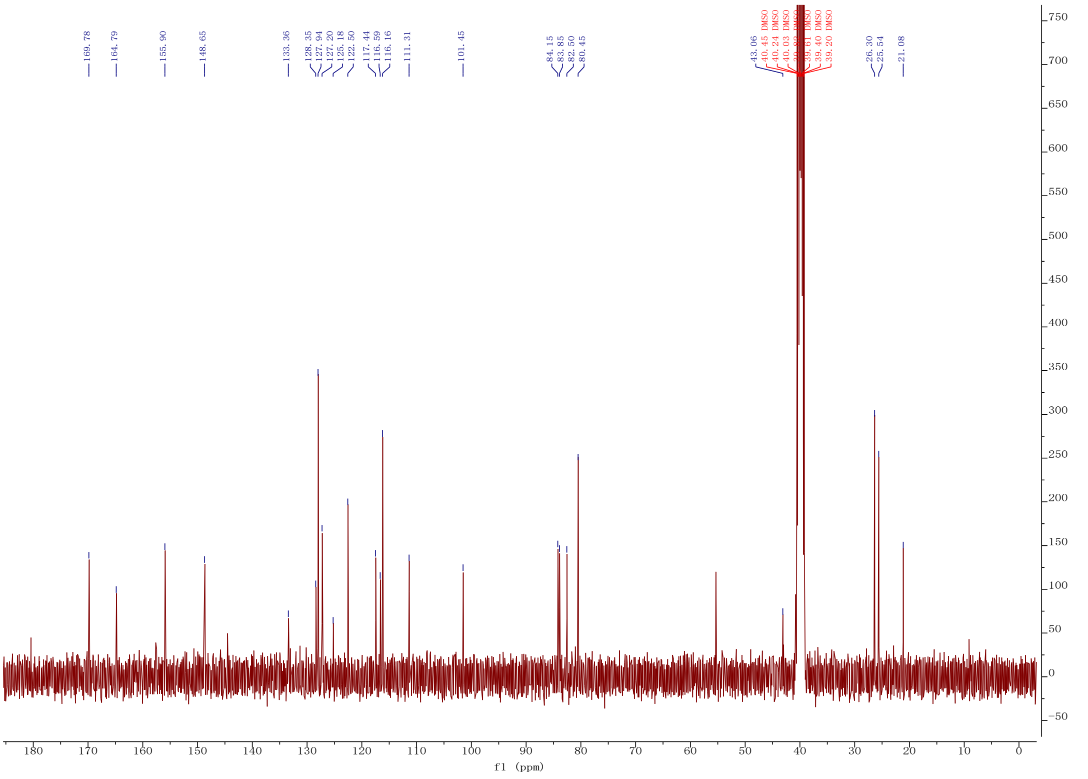
169.78
164.79
155.90
148.65
133.36
128.35
127.94
127.20
125.18
122.50
117.44
116.59
116.16
111.31
101.45
84.15
83.85
82.50
80.43
43.06
40.45 DMSO
40.24 DMSO
40.03 DMSO
39.61 DMSO
39.40 DMSO
39.20 DMSO
26.30
25.54
21.08
f1 (ppm)

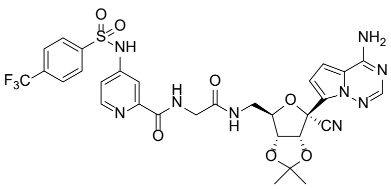

12z

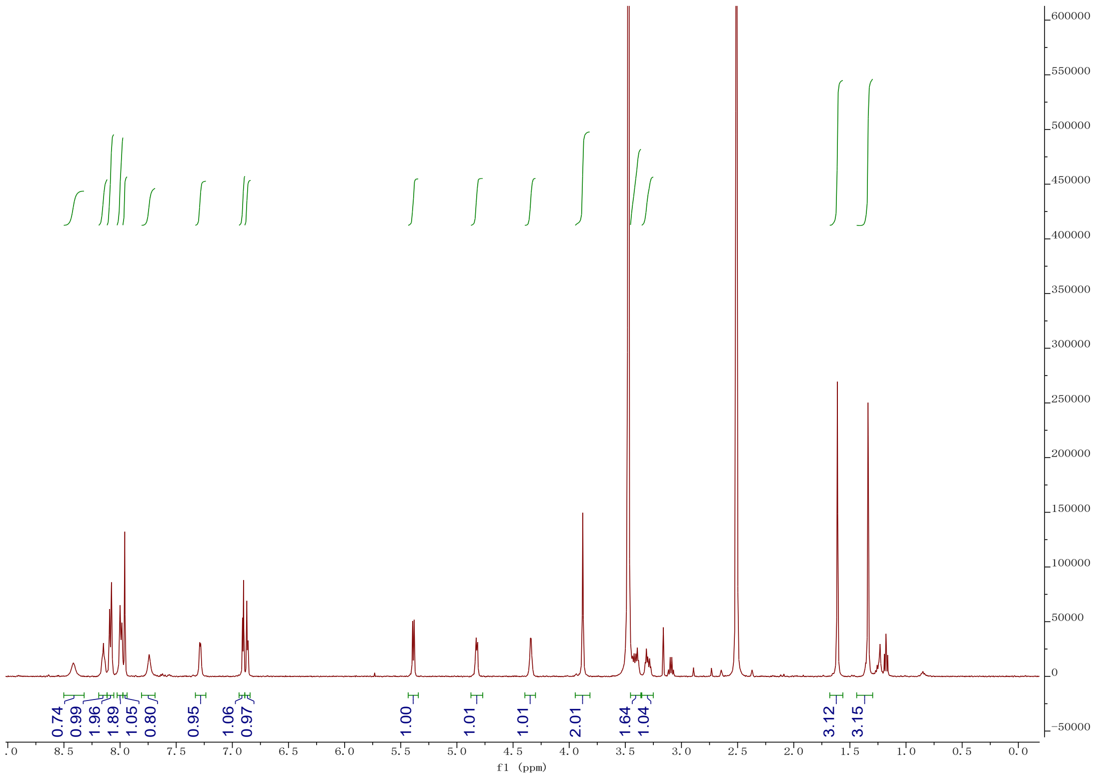

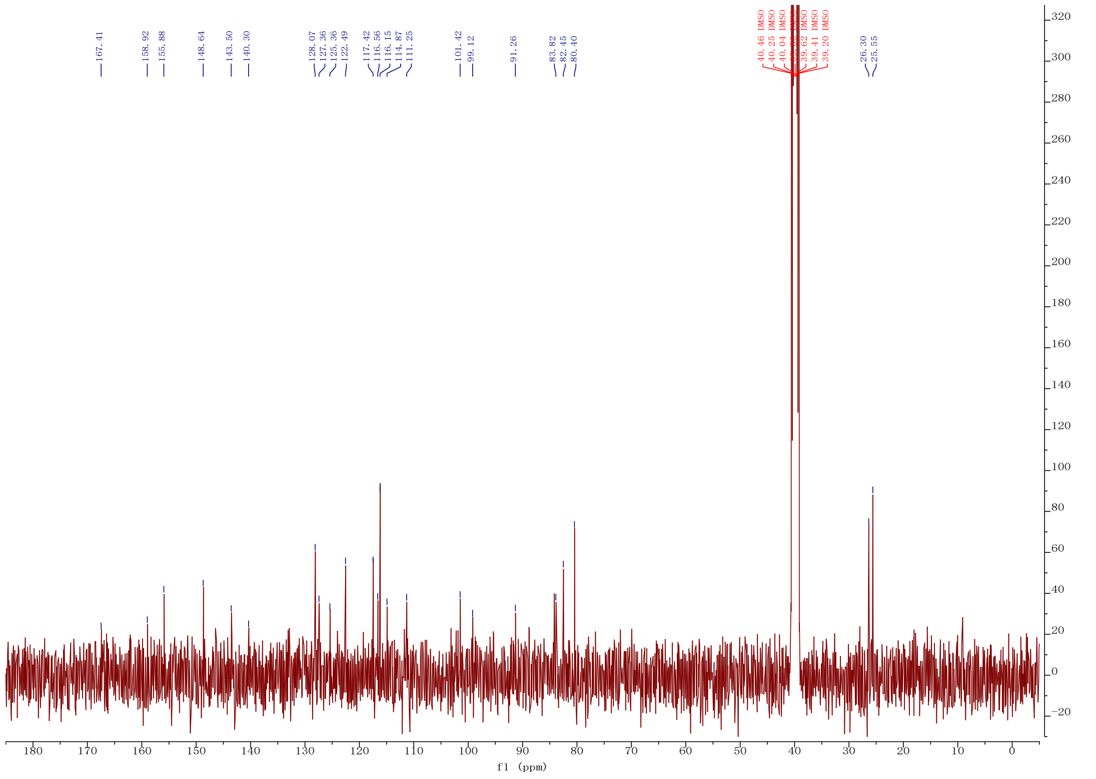

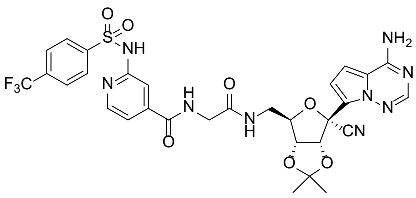

12aa

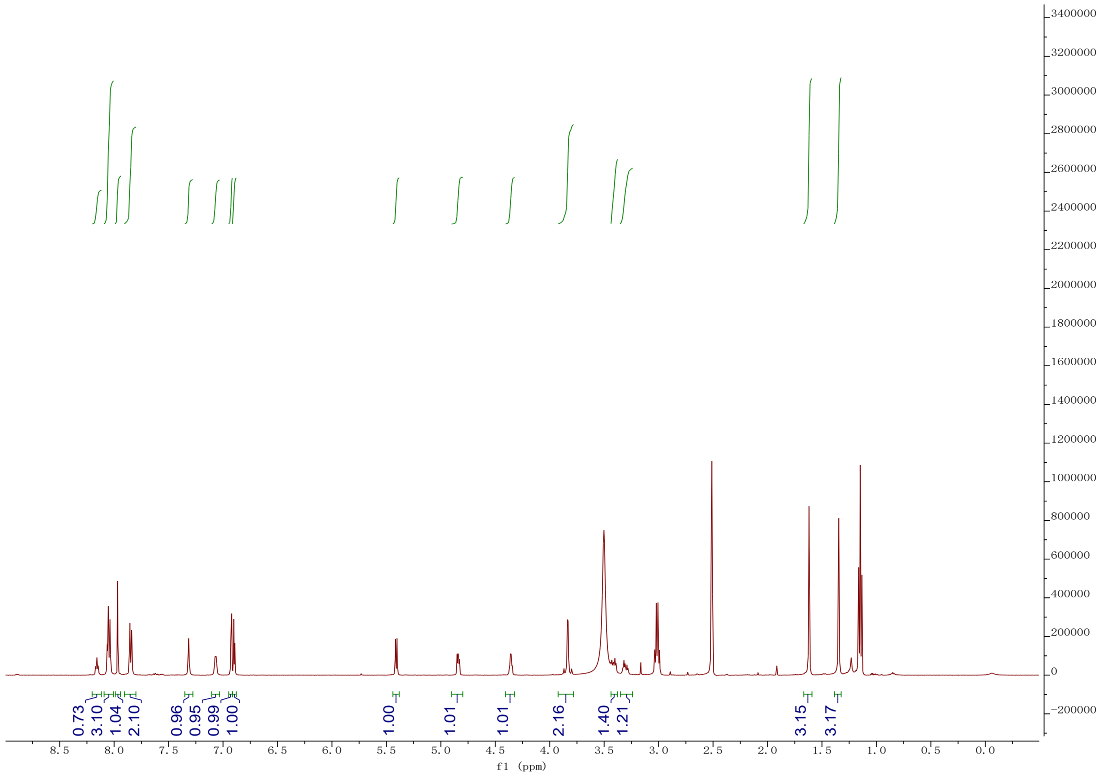

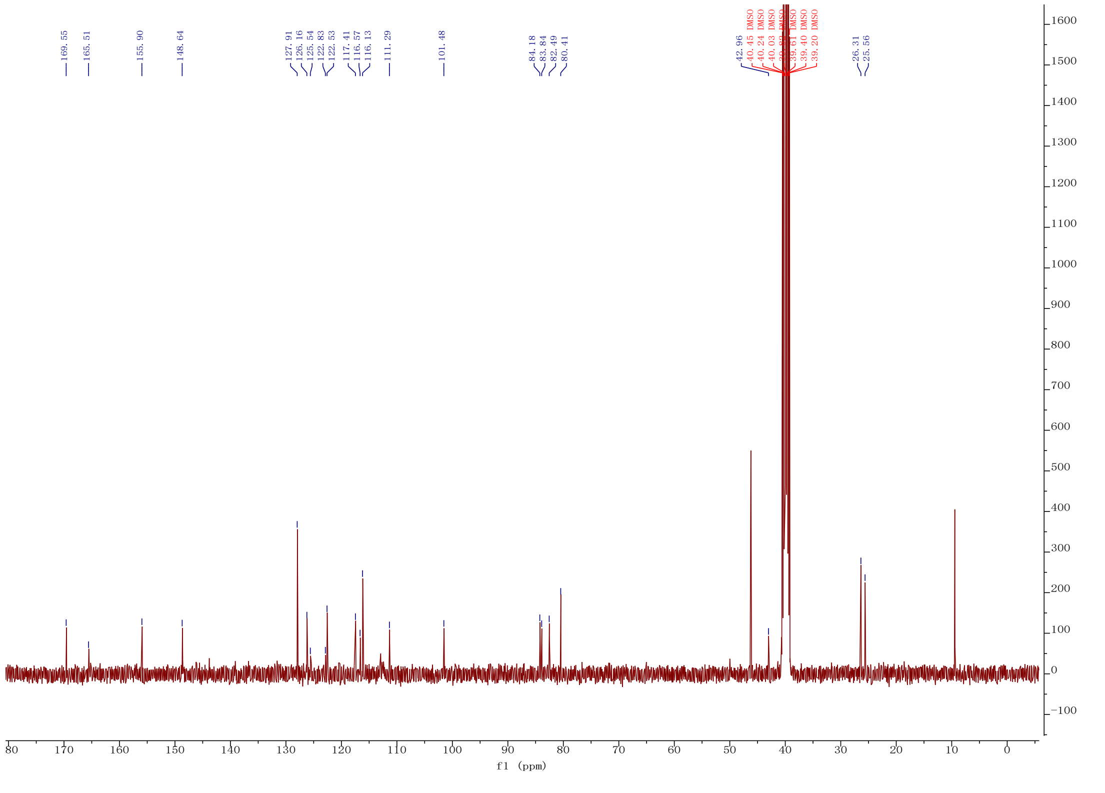

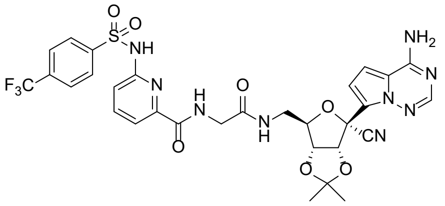
NH
F3C
N
H
N
O
O
N
H
NH2
N
N
N
CN
O
O
O
S
O
O

12ab

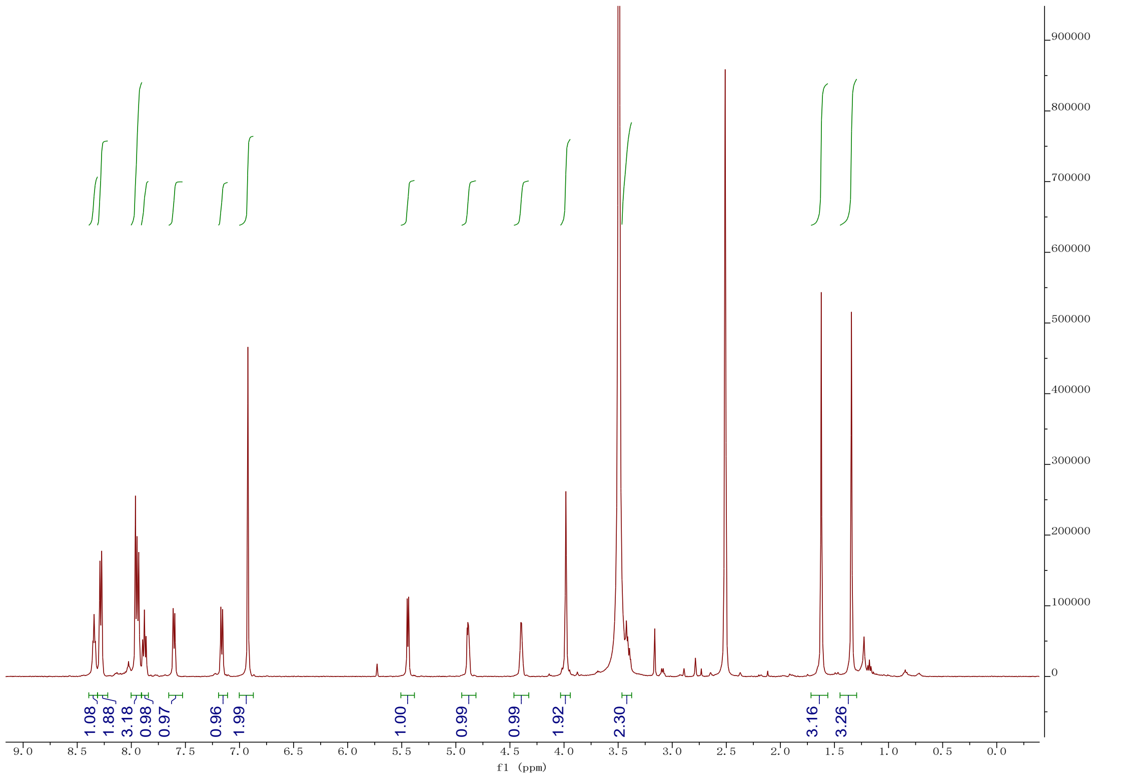
1.08
1.88
3.18
0.98
0.97
0.96
1.99
1.00
0.99
0.99
1.92
2.30
3.16
3.26
900000
800000
700000
600000
500000
400000
300000
200000
100000
0
9.0 8.5 8.0 7.5 7.0 6.5 6.0 5.5 5.0 4.5 4.0 3.5 3.0 2.5 2.0 1.5 1.0 0.5 0.0
f1 (ppm)

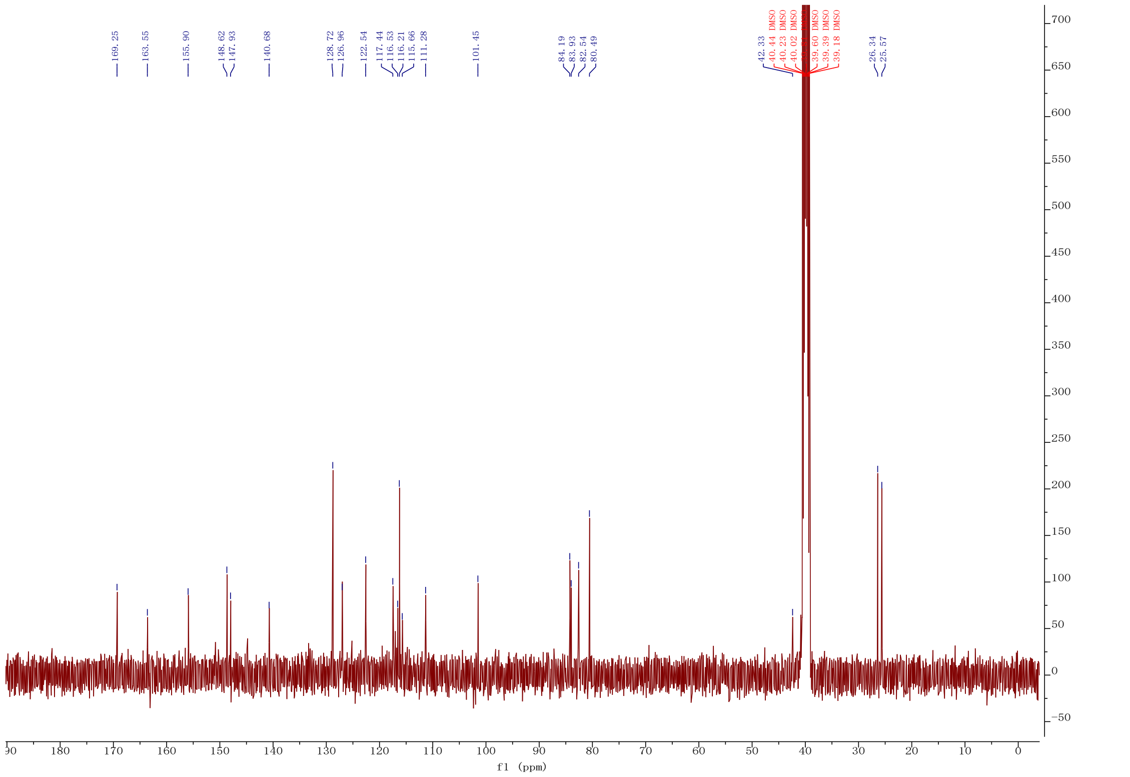
169.25
163.55
155.90
148.62
147.93
140.68
128.72
126.96
122.54
117.44
116.53
116.21
115.66
111.28
101.45
84.19
83.93
82.54
80.49
42.33
40.44 DMSO
40.23 DMSO
40.02 DMSO
39.60 DMSO
39.39 DMSO
39.18 DMSO
26.34
25.57
700
650
600
550
500
450
400
350
300
250
200
150
100
50
0
-50
180 170 160 150 140 130 120 110 100 90 80 70 60 50 40 30 20 10 0
f1 (ppm)

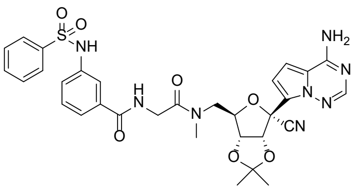

O
S
O
NH
H
N
O
N
O
O
O
CN
NH2
N
N
N
N
O

18a

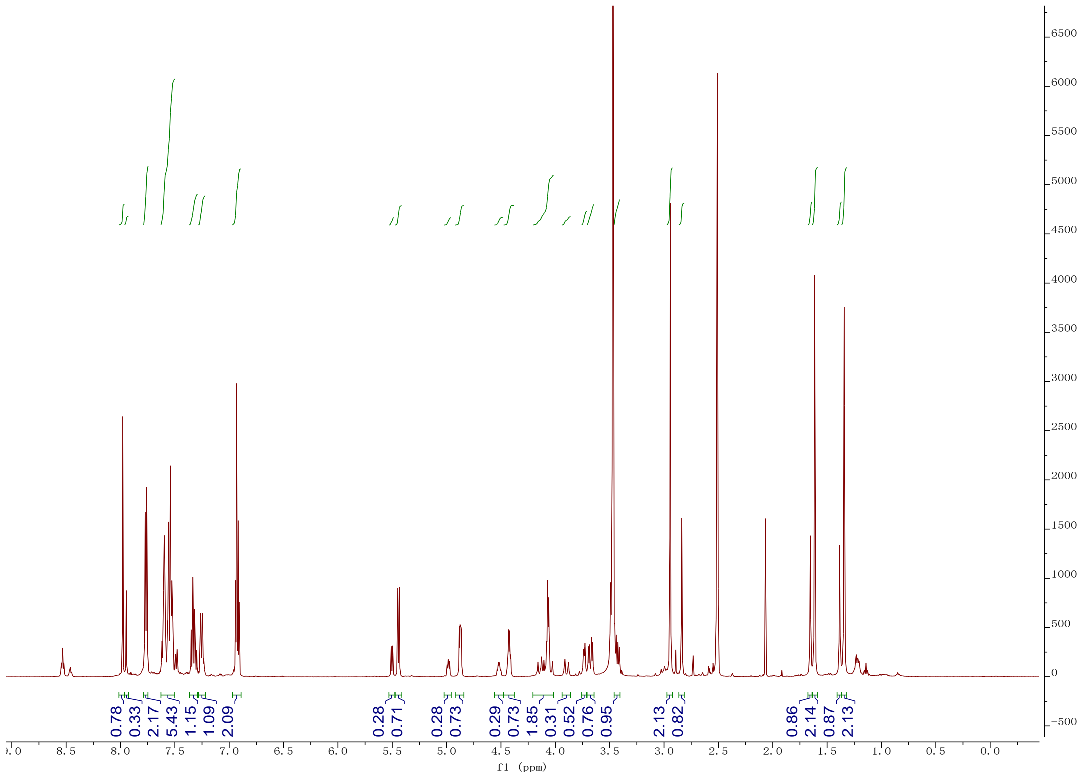

0.78
0.33
2.17
5.43
1.15
1.09
2.09
0.28
0.71
0.28
0.73
0.29
0.73
1.85
0.31
0.52
0.76
0.95
2.13
0.82
0.86
2.14
0.87
2.13
f1 (ppm)

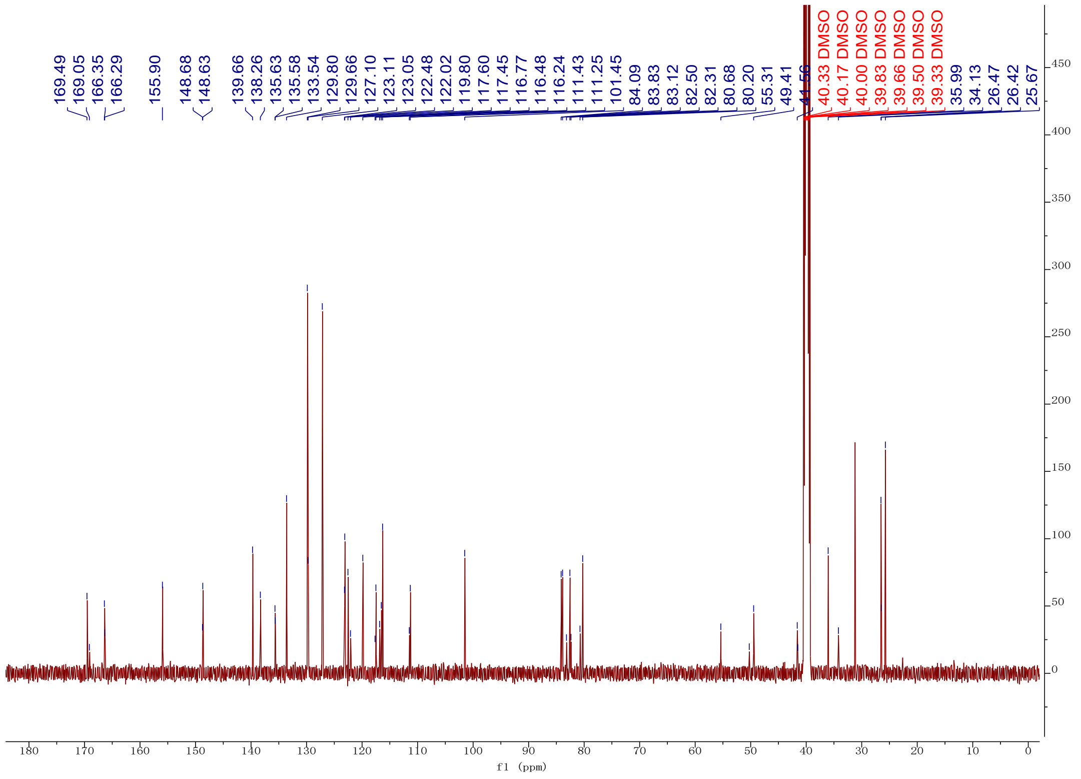

169.49
169.05
166.35
166.29
155.90
148.68
148.63
139.66
138.26
135.63
135.58
133.54
129.80
129.66
127.10
123.11
123.05
122.48
122.02
119.80
117.60
117.45
116.77
116.48
116.24
111.43
111.25
101.45
84.09
83.83
83.12
82.50
82.31
80.68
80.20
55.31
49.41
40.33 DMSO
40.17 DMSO
40.00 DMSO
39.83 DMSO
39.66 DMSO
39.50 DMSO
39.33 DMSO
35.99
34.13
26.47
26.42
25.67
f1 (ppm)

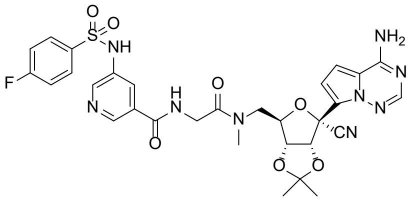

18b

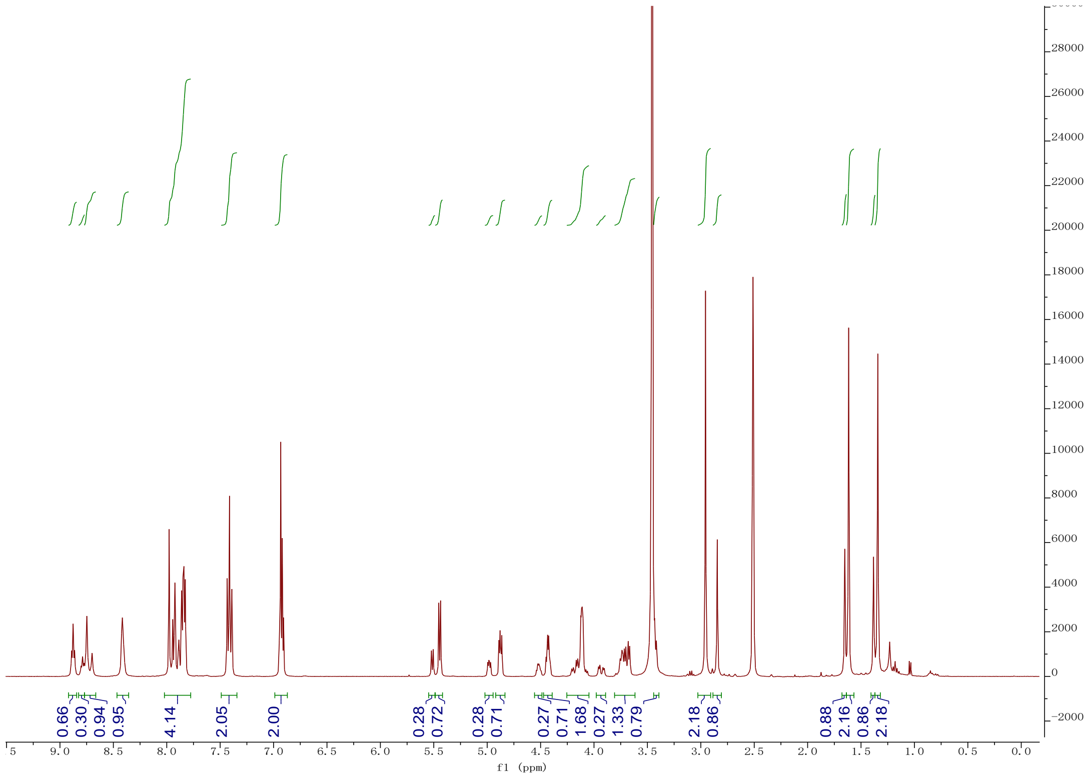
0.66 0.30 0.94 0.95 4.14 2.05 2.00 0.28 0.72 0.28 0.71 0.27 0.71 1.68 0.27 1.33 0.79 2.18 0.86 0.88 2.16 0.86 2.18
f1 (ppm)

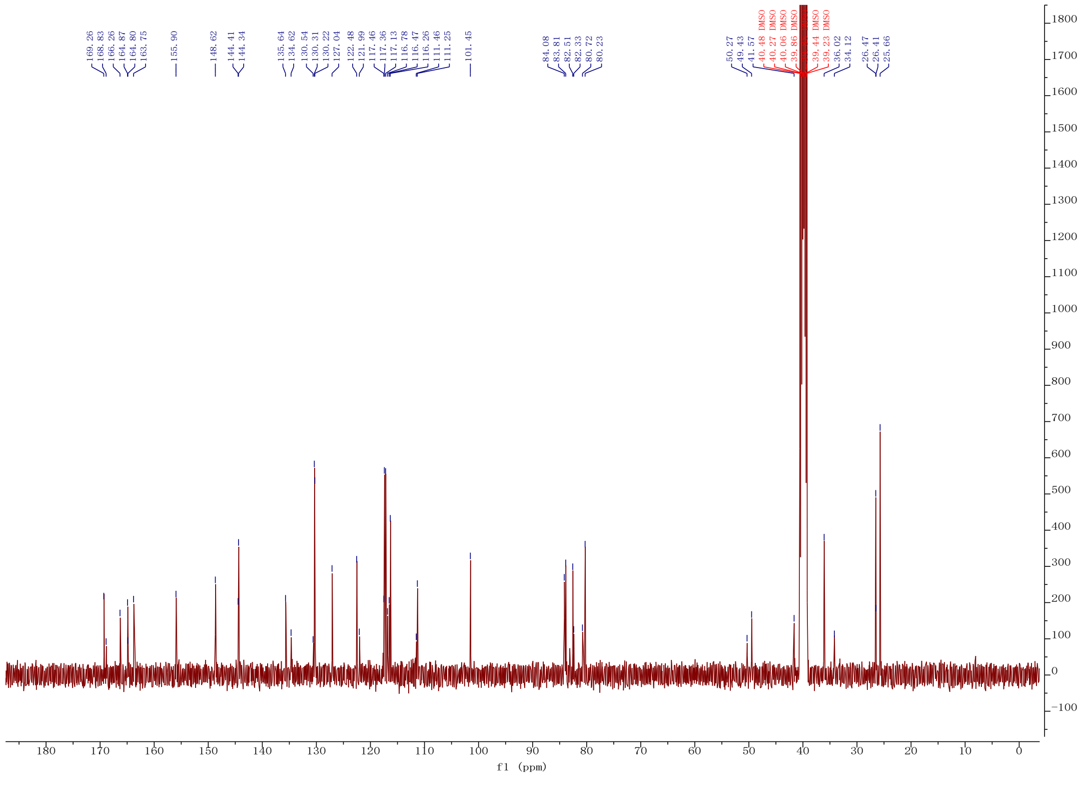
169.26 168.83 166.26 164.87 164.80 163.75 155.90 148.62 144.41 144.34 135.64 134.62 130.54 130.31 130.22 127.04 122.48 121.99 117.46 117.36 117.13 116.78 116.47 116.26 111.46 111.25 101.45 84.08 83.81 82.51 82.33 80.72 80.23 50.27 49.43 41.57 40.48 DMSO 40.27 DMSO 40.06 DMSO 39.86 DMSO 39.44 DMSO 39.23 DMSO 36.02 34.12 26.47 26.41 25.66
f1 (ppm)

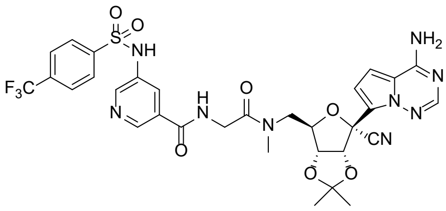

18c

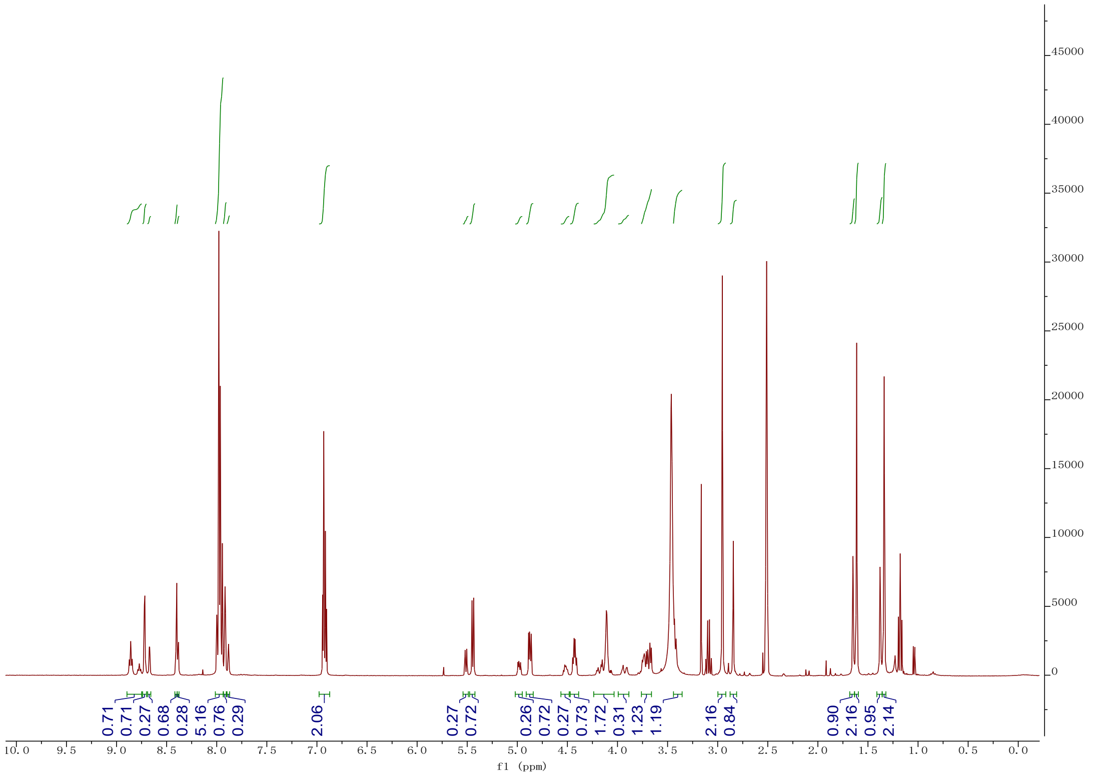
0.71
0.71
0.27
0.68
0.28
5.16
0.76
0.29
2.06
0.27
0.72
0.26
0.72
0.27
0.73
1.72
0.31
1.23
1.19
2.16
0.84
0.90
2.16
0.95
2.14
f1 (ppm)

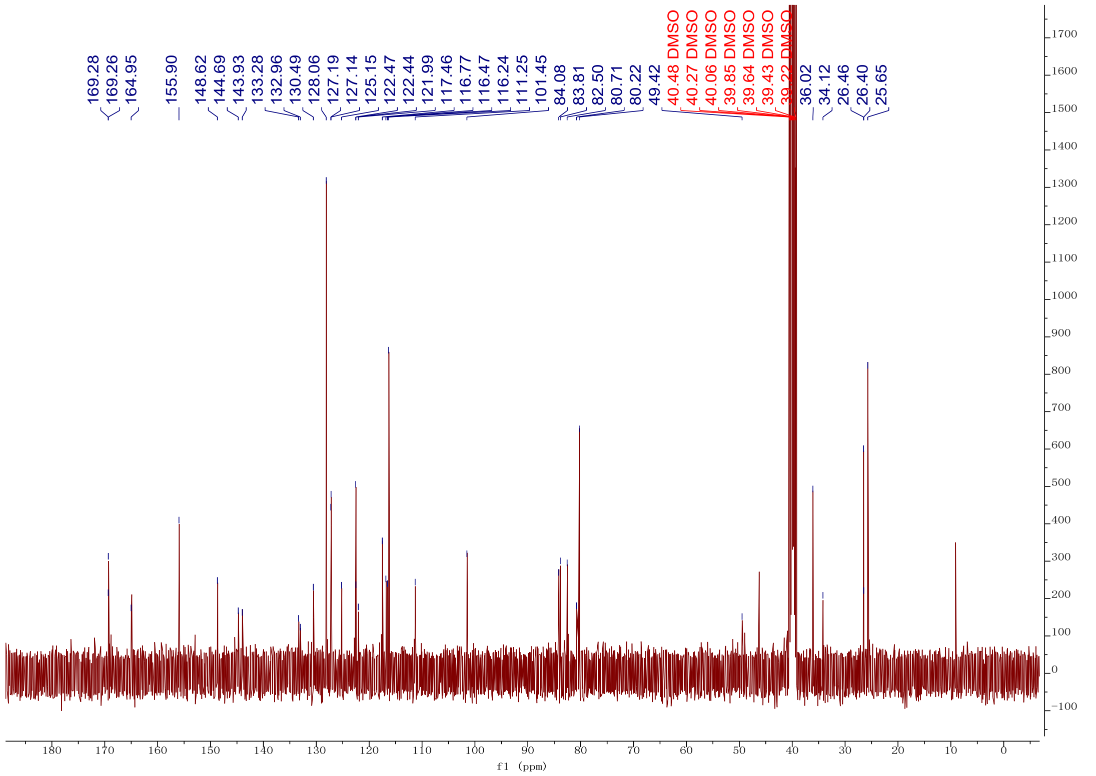
169.28
169.26
164.95
155.90
148.62
144.69
143.93
133.28
132.96
130.49
128.06
127.19
127.14
125.15
122.47
122.44
121.99
117.46
116.77
116.47
116.24
111.25
101.45
84.08
83.81
82.50
80.71
80.22
49.42
40.48 DMSO
40.27 DMSO
40.06 DMSO
39.85 DMSO
39.64 DMSO
39.43 DMSO
39.22 DMSO
36.02
34.12
26.46
26.40
25.65
f1 (ppm)

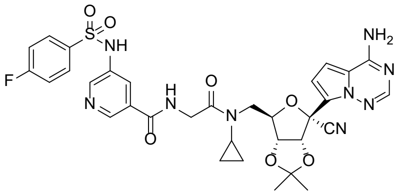

18d

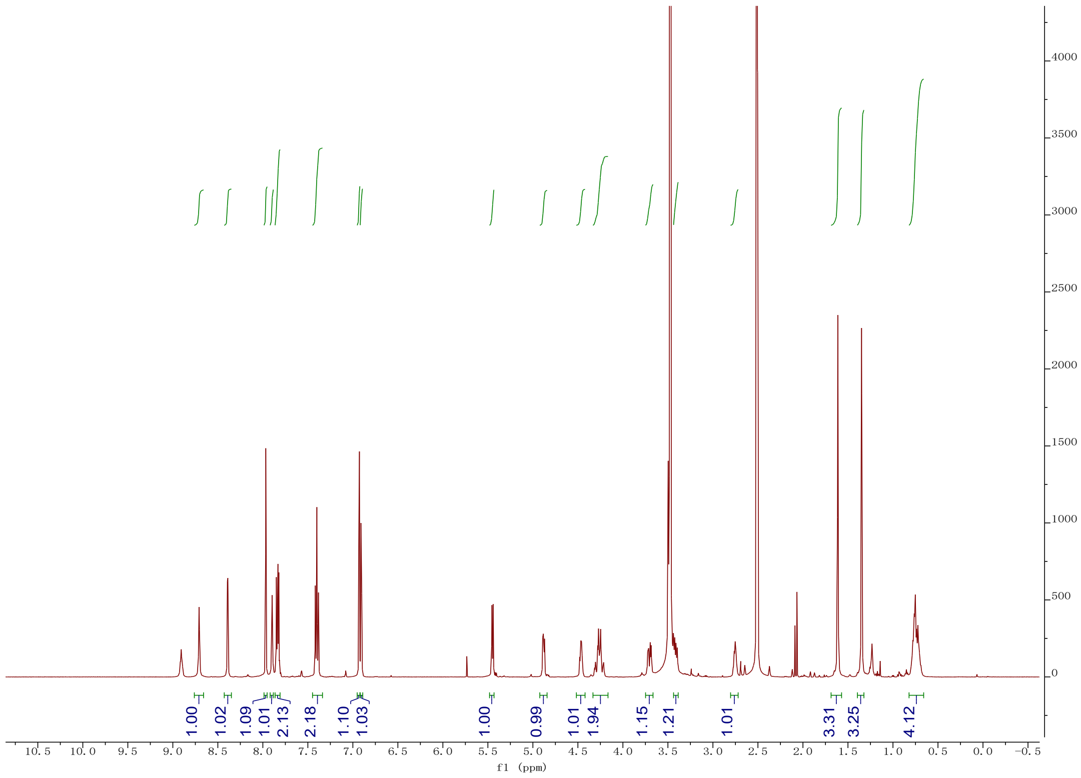
1.00
1.02
1.09
1.01
2.13
2.18
1.10
1.03
1.00
0.99
1.01
1.94
1.15
1.21
1.01
3.31
3.25
4.12
f1 (ppm)

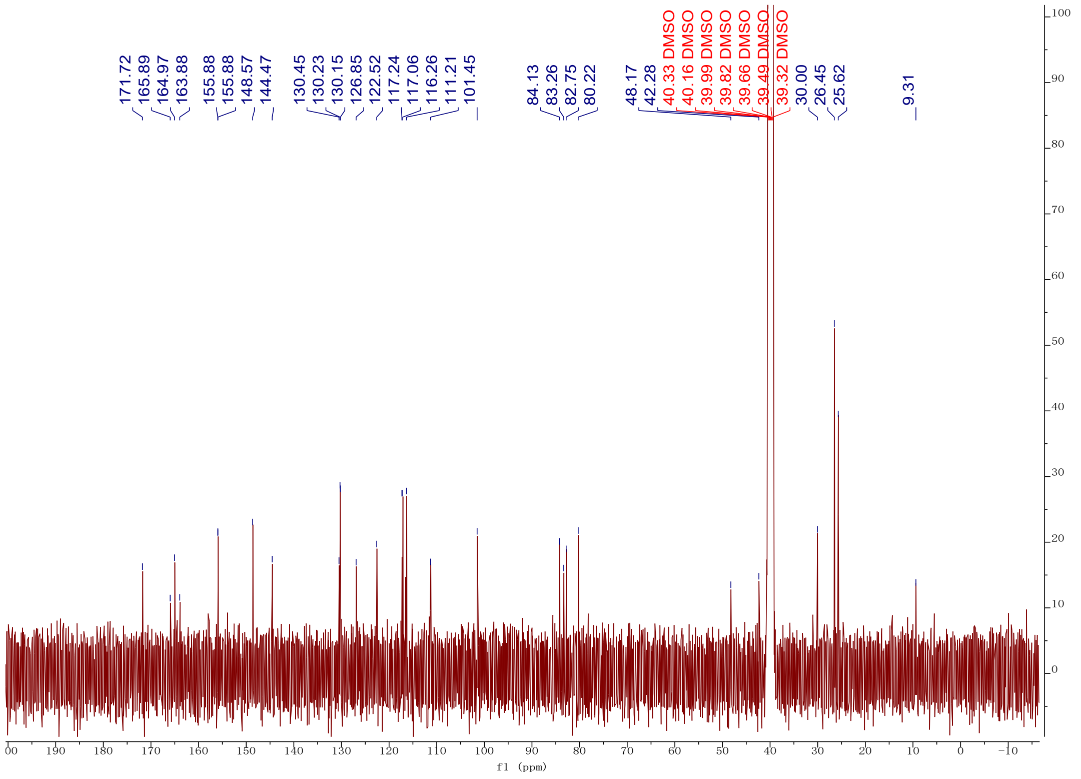
171.72
165.89
164.97
163.88
155.88
155.88
148.57
144.47
130.45
130.23
130.15
126.85
122.52
117.24
117.06
116.26
111.21
101.45
84.13
83.26
82.75
80.22
48.17
42.28
40.33 DMSO
40.16 DMSO
39.99 DMSO
39.82 DMSO
39.66 DMSO
39.49 DMSO
39.32 DMSO
30.00
26.45
25.62
9.31
f1 (ppm)

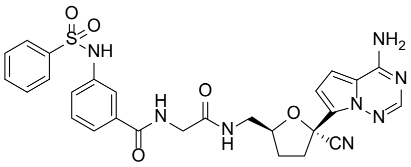

29a

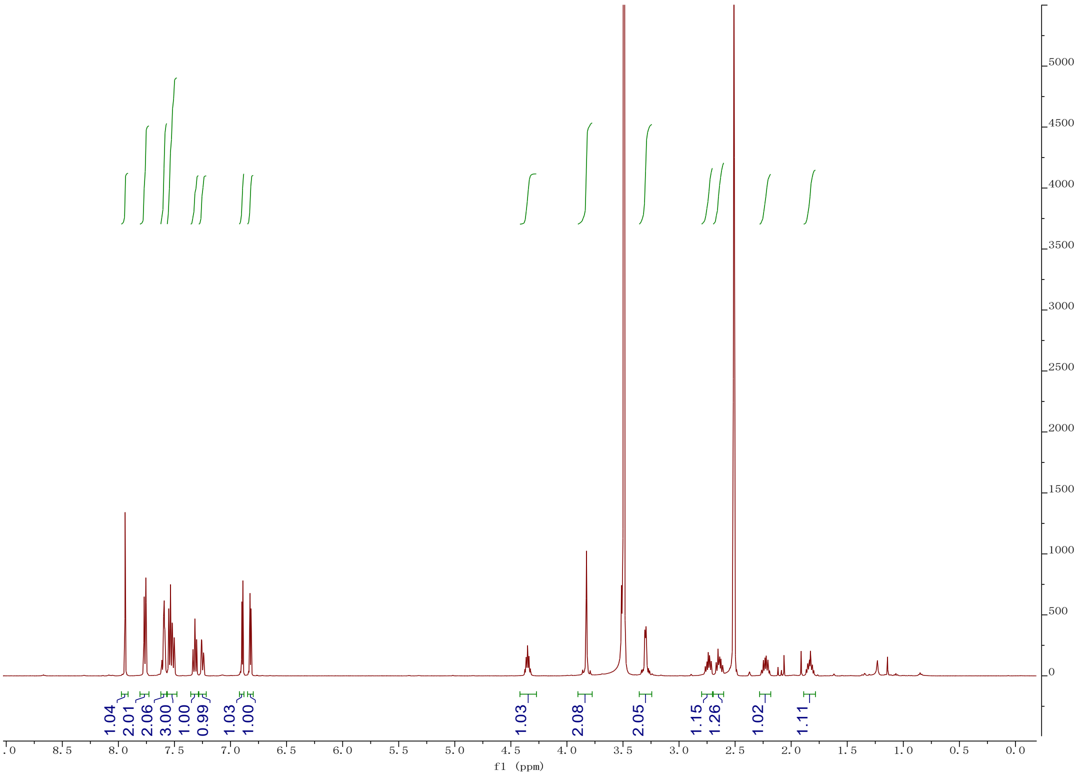
1.04
2.01
2.06
3.00
1.00
0.99
1.03
1.00
1.03
2.08
2.05
1.15
1.26
1.02
1.11
f1 (ppm)

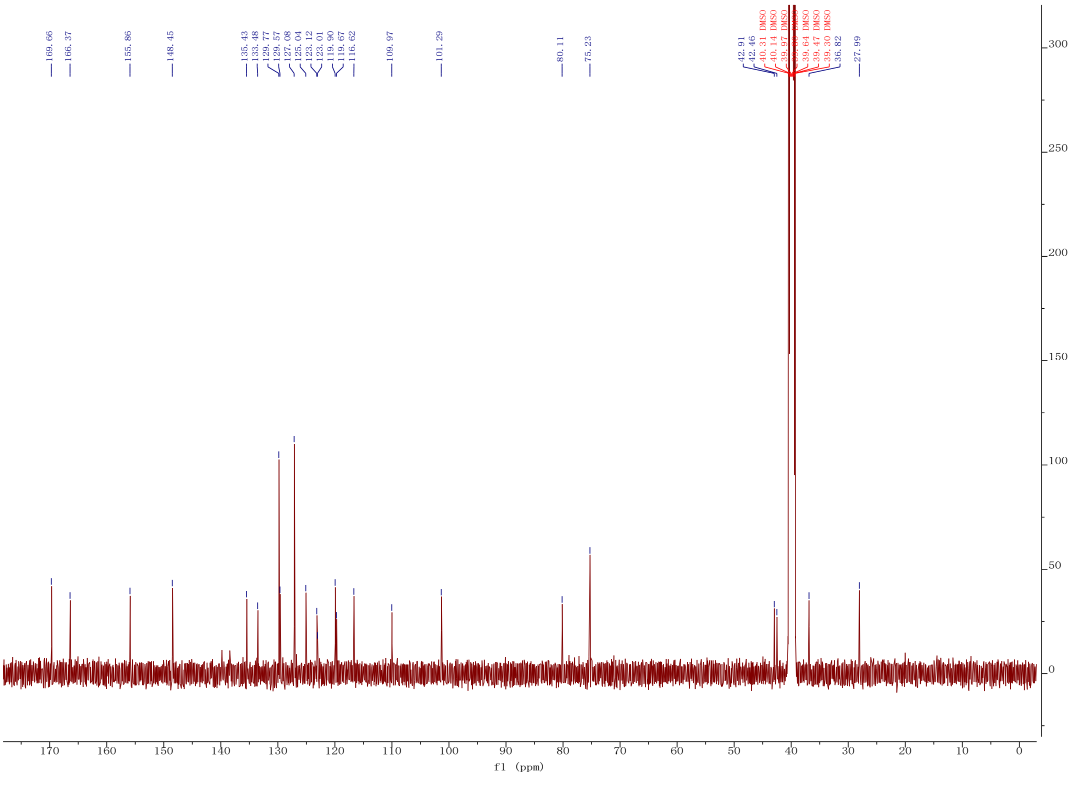
169.66
166.37
155.86
148.45
135.43
133.48
129.77
129.57
127.08
125.04
123.12
123.01
119.90
119.67
116.62
109.97
101.29
80.11
75.23
42.91
42.46
40.31 DMSO
40.14 DMSO
39.97 DMSO
39.64 DMSO
39.47 DMSO
39.30 DMSO
36.82
27.99
f1 (ppm)

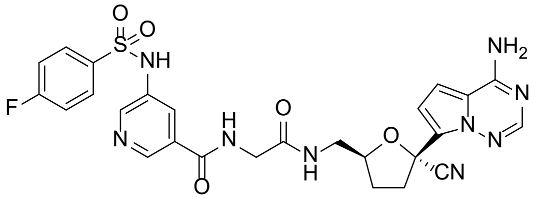

29b

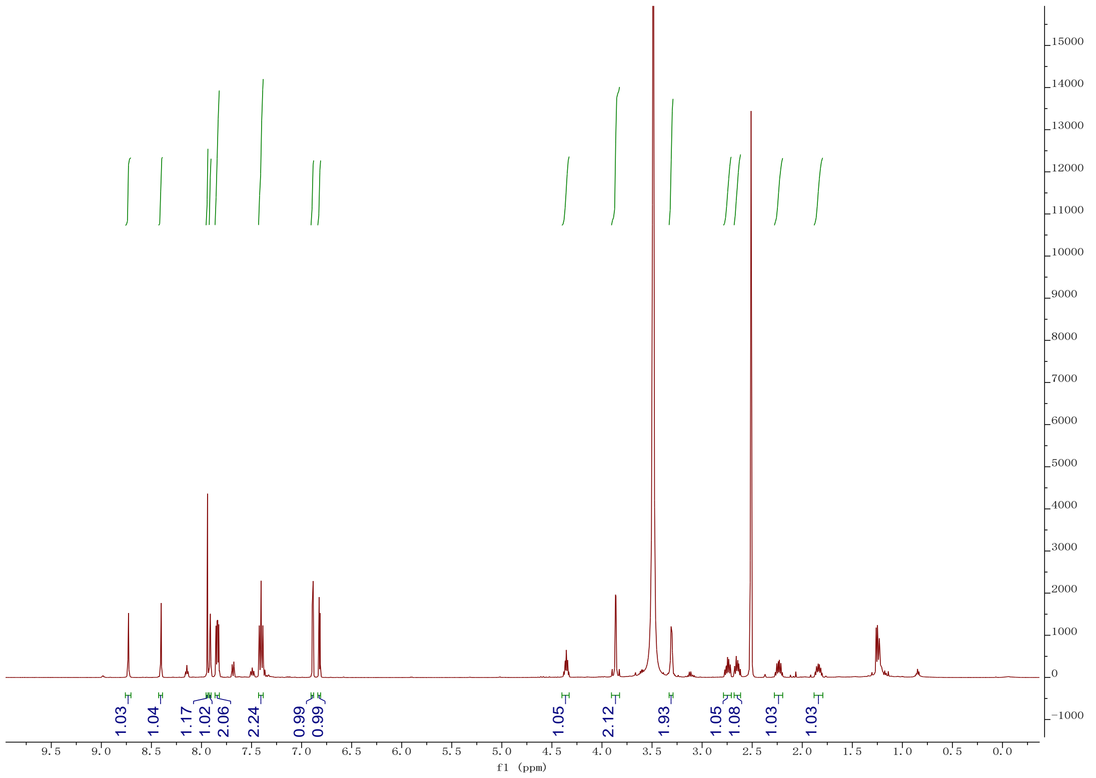

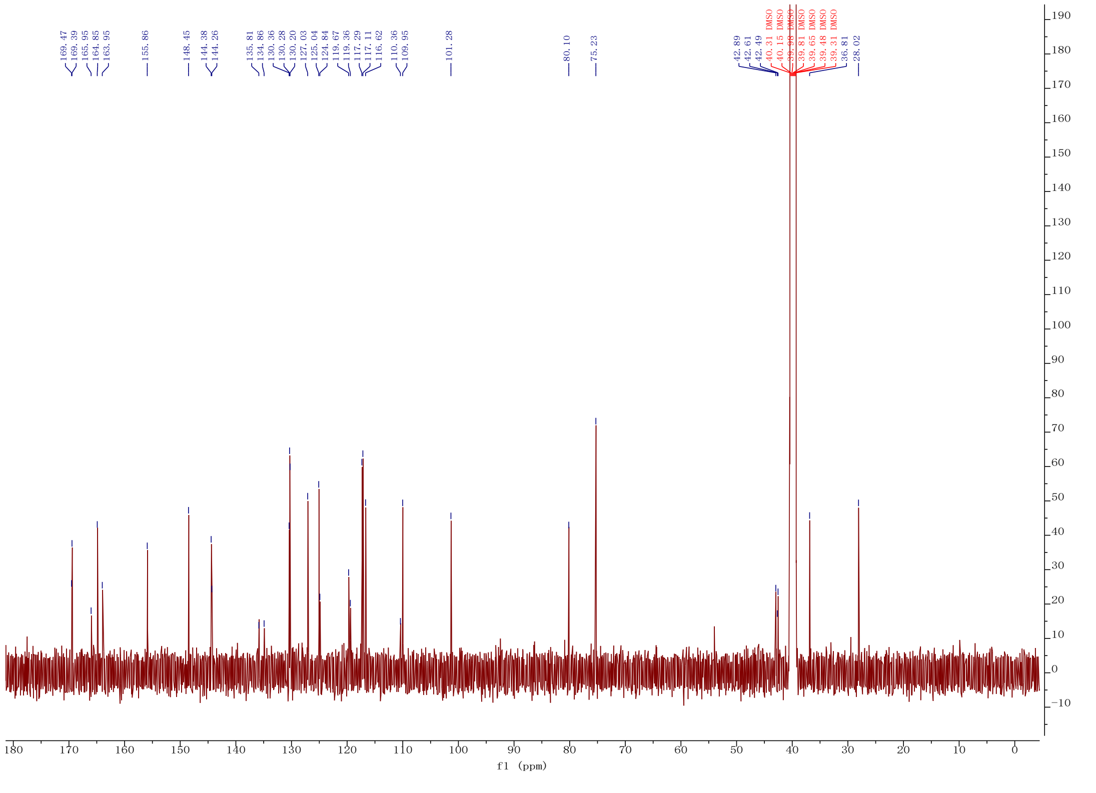

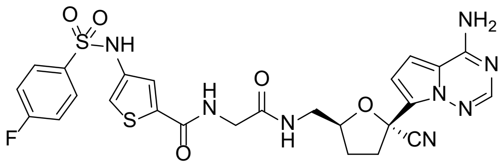

29c

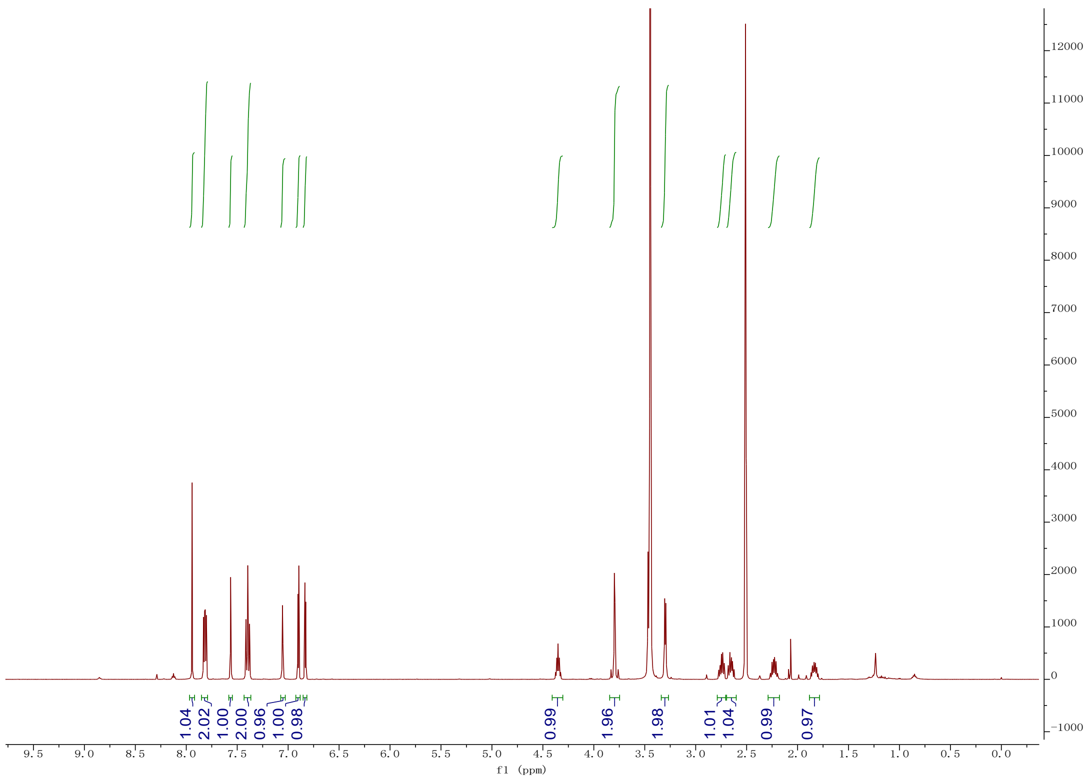

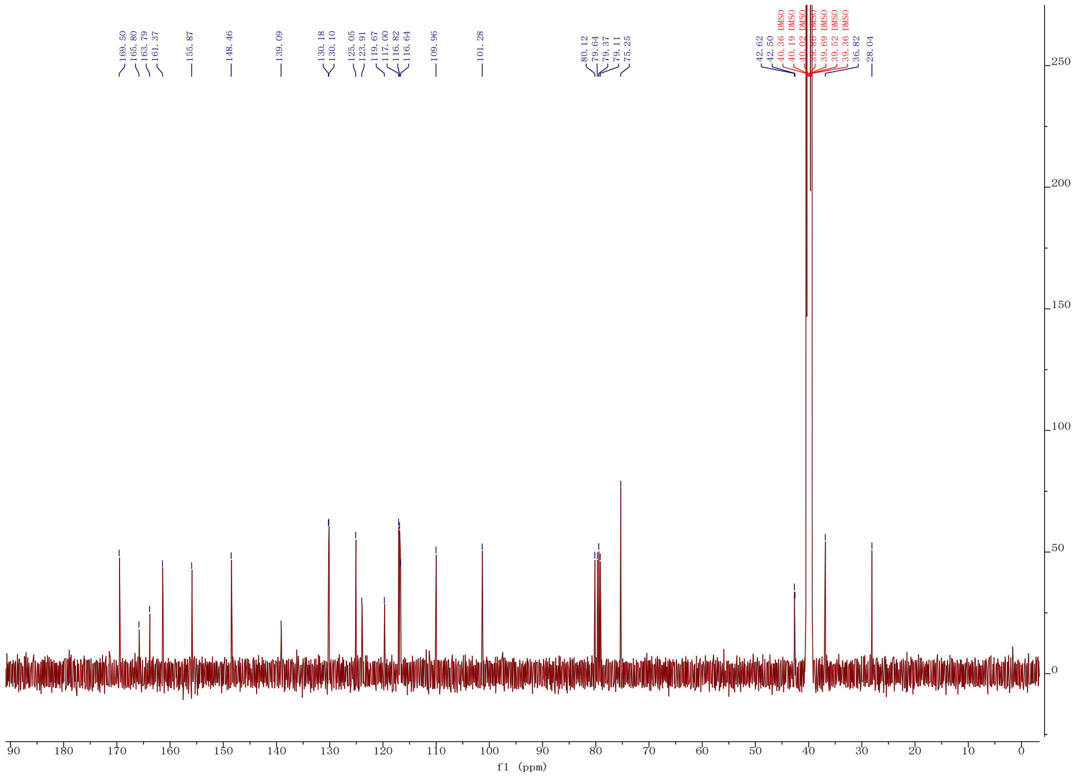

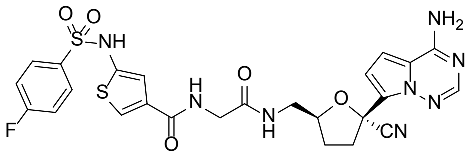
NH2
N
N
N
O
CN
N
H
O
H
N
O
S
NH
O
S
O
F

29d

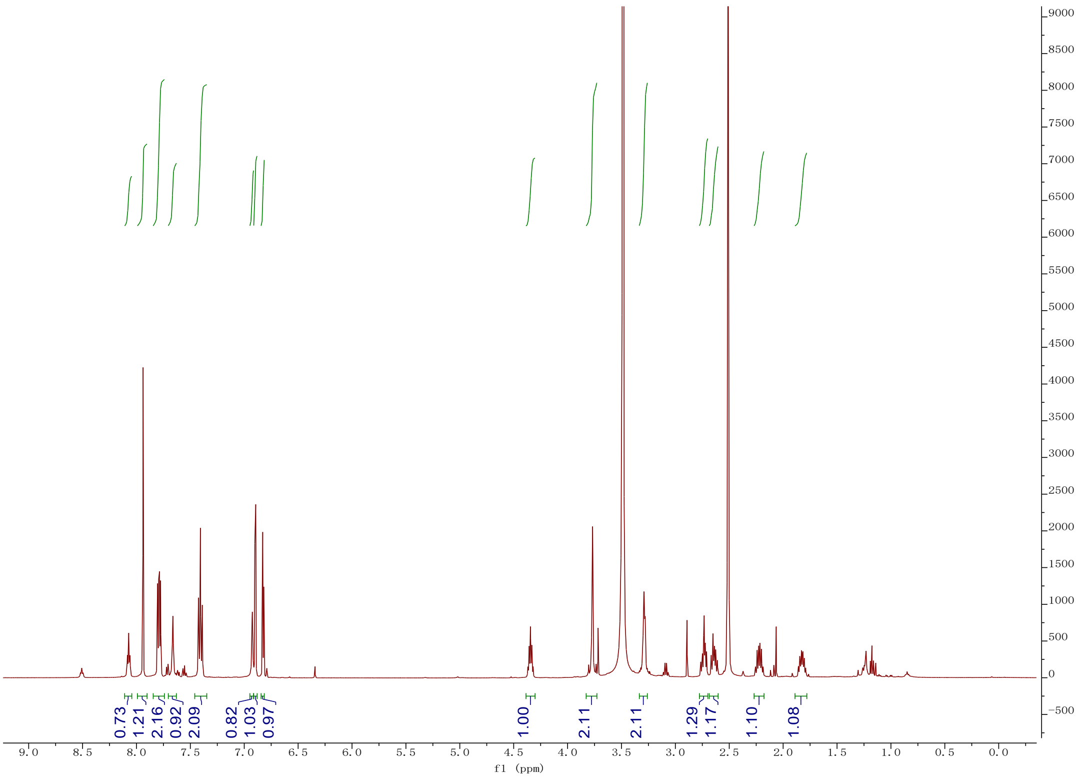
0.73
1.21
2.16
0.92
2.09
0.82
1.03
0.97
1.00
2.11
2.11
1.29
1.17
1.10
1.08
f1 (ppm)

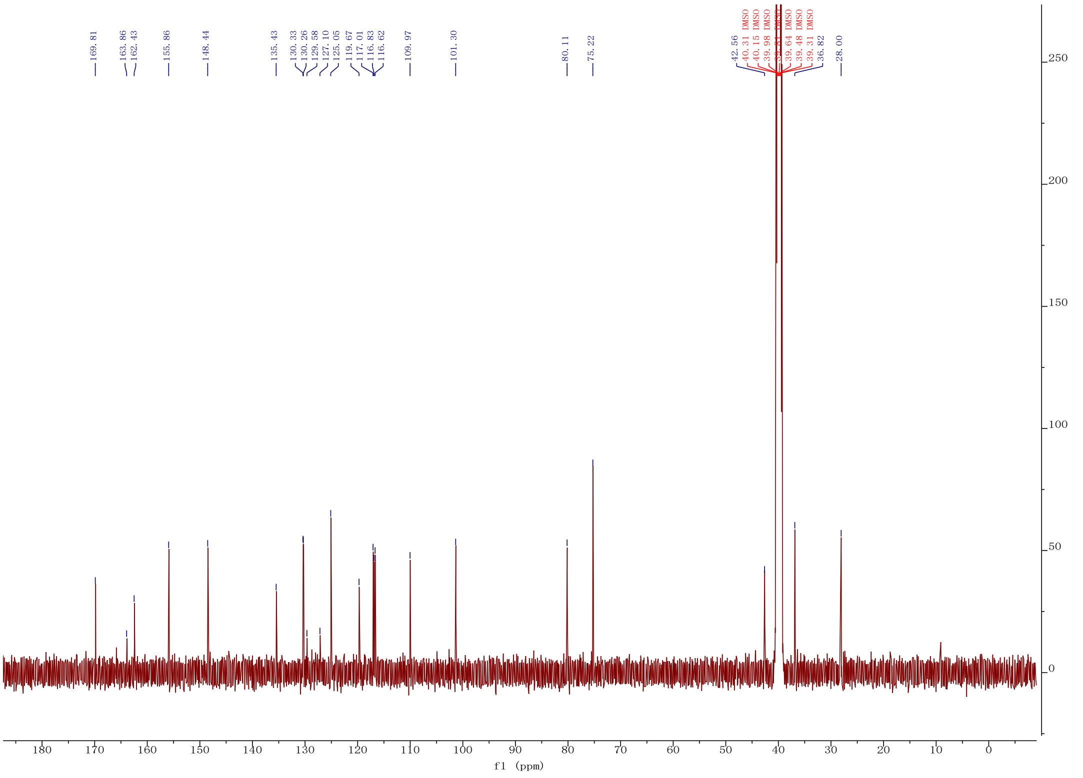
169.81
163.86
162.43
155.86
148.44
135.43
130.33
130.26
129.58
127.10
125.05
119.67
117.01
116.83
116.62
109.97
101.30
80.11
75.22
42.56
40.31 DMSO
40.15 DMSO
39.98 DMSO
39.64 DMSO
39.48 DMSO
39.31 DMSO
36.82
28.00
f1 (ppm)

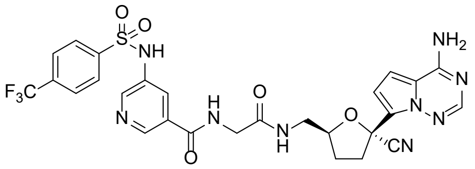
NH2
N
N
N
N
O
CN
O
H
N
O
N
H
O
N
NH
S
O
O
F3C

29e

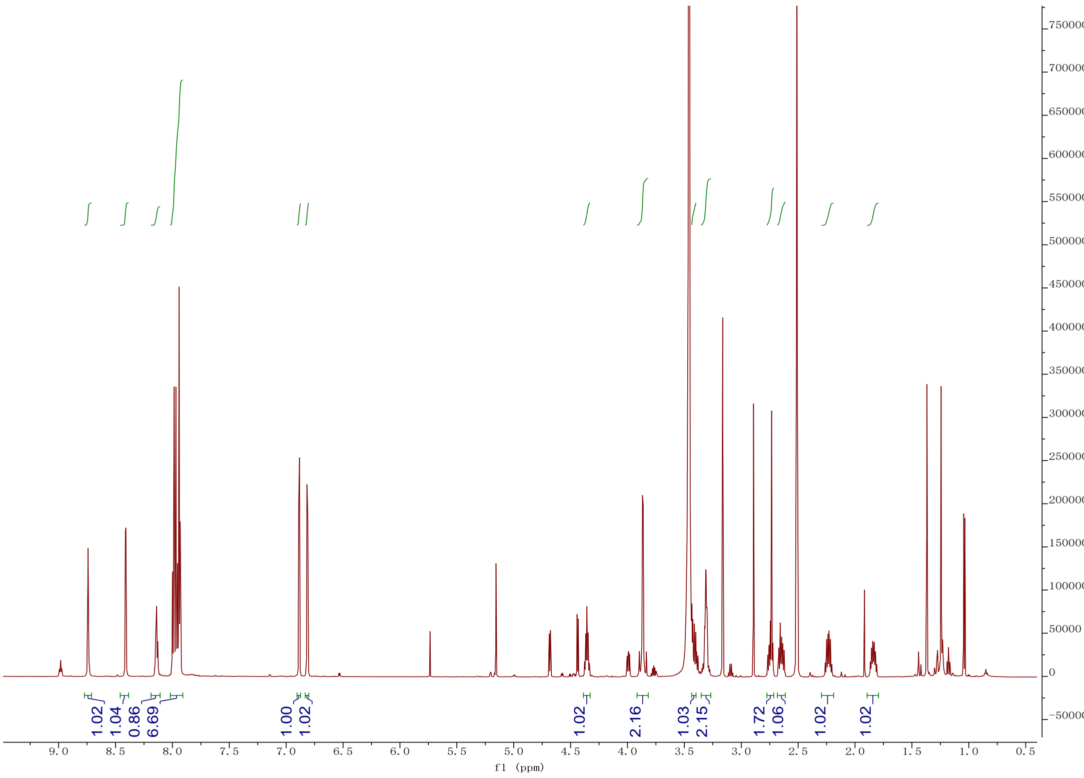
1.02
1.04
0.86
6.69
1.00
1.02
1.02
2.16
1.03
2.15
1.72
1.06
1.02
1.02
f1 (ppm)

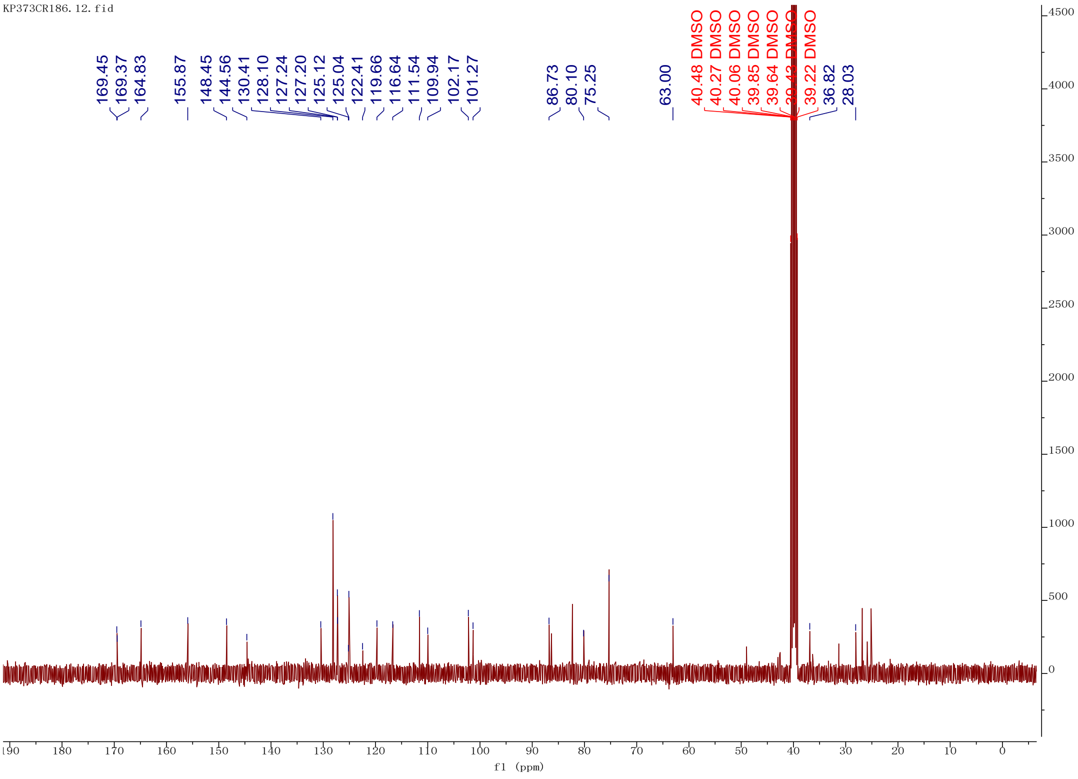
KP373CR186.12.fid
169.45
169.37
164.83
155.87
148.45
144.56
130.41
128.10
127.24
127.20
125.12
125.04
122.41
119.66
116.64
111.54
109.94
102.17
101.27
86.73
80.10
75.25
63.00
40.48 DMSO
40.27 DMSO
40.06 DMSO
39.85 DMSO
39.64 DMSO
39.22 DMSO
36.82
28.03
f1 (ppm)

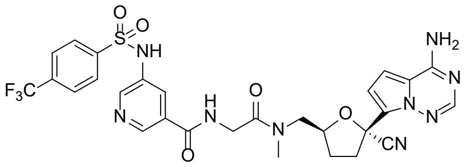

33

KP456CR186.10.fid

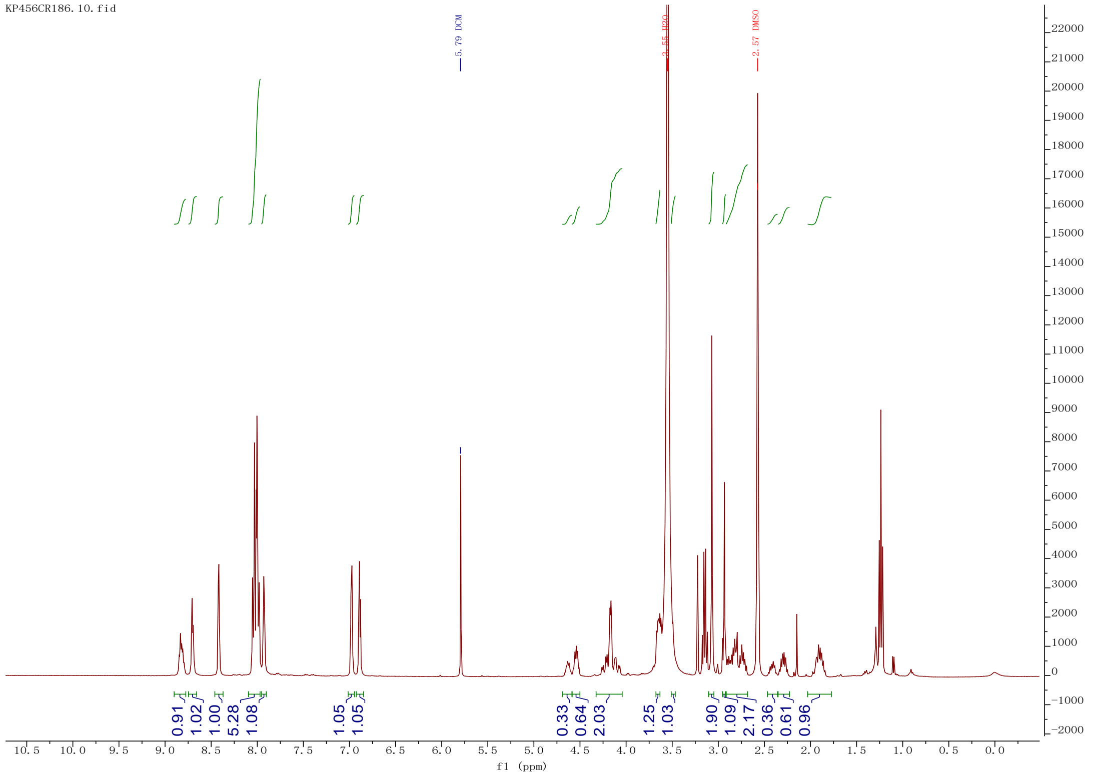

KP456CR186.12.fid

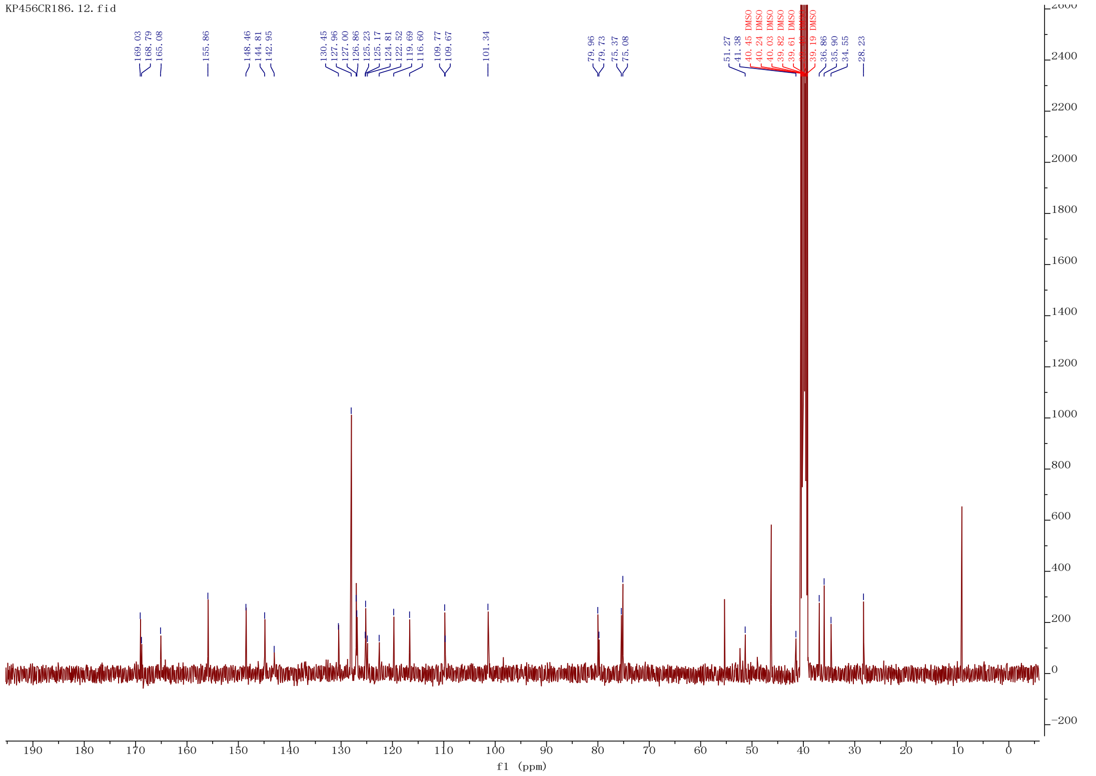

Supplement: Supplement 1 [file media-1.pdf]
